# Cross-species conserved miRNA as biomarker of radiation injury over a wide dose range using nonhuman primate model

**DOI:** 10.1371/journal.pone.0311379

**Published:** 2024-11-21

**Authors:** Nabarun Chakraborty, George Dimitrov, Swapna Kanan, Alexander Lawrence, Candance Moyler, Aarti Gautam, Oluseyi O. Fatanmi, Stephen Y. Wise, Alana D. Carpenter, Rasha Hammamieh, Vijay K. Singh

**Affiliations:** 1 Medical Readiness Systems Biology, CMPN, Walter Reed Army Institute of Research, Silver Spring, MD, United States of America; 2 Vysnova, Inc., Landover, MD, United States of America; 3 Oak Ridge Institute for Science and Education (ORISE), MD, United States of America; 4 Division of Radioprotectants, Department of Pharmacology and Molecular Therapeutics, F. Edward Hébert School of Medicine, Uniformed Services University of the Health Sciences, Bethesda, MD, United States of America; 5 Armed Forces Radiobiology Research Institute, Uniformed Services University of the Health Sciences, Bethesda, MD, United States of America; Huashan Hospital Fudan University, CHINA

## Abstract

Multiple accidents in nuclear power plants and the growing concerns about the misuse of radiation exposure in warfare have called for the rapid determination of absorbed radiation doses (RDs). The latest findings about circulating microRNA (miRNAs) using several animal models revealed considerable promises, although translating this knowledge to clinics remains a major challenge. To address this issue, we randomly divided 36 nonhuman primates (NHPs) into six groups and exposed these groups to six different radiation doses ranging from 6.0–8.5 Gy in increments of 0.5 Gy. Serum samples were collected pre-irradiation as well as three post-irradiation timepoints, namely 1, 2 and 6 days post-total body irradiation (TBI). Generated from a deep sequencing platform, the miRNA reads were multi-variate analyzed to find the differentially expressed putative biomarkers that were linked to RDs, time since irradiation (TSI) and sex. To increase these biomarkers’ translational potential, we aligned the NHP-miRNAs’ sequences and their functional responses to humans following an *in-silico* routine. Those miRNAs, which were sequentially and functionally conserved between NHPs and humans, were down selected for further analysis. A linear regression model identified miRNA markers that were consistently regulated with increasing RD but independent TSI. Likewise, a set of potential TSI-markers were identified that consistently shifted with increasing TSI, but independent of RD. Additional molecular analysis found a considerable gender bias in the low-ranges of doses when the risk to radiation-induced fatality was low. Bionetworks linked to cell quantity and cell invasion were significantly altered between the survivors and decedents. Using these biomarkers, an assay could be developed to retrospectively determine the RD and TSI with high translational potential. Ultimately, this knowledge can lead to precise and personalized medicine.

## Introduction

Radiological or nuclear events caused by the deliberate or inadvertent release of radiation can have life-threatening consequences. Identifying solutions for nuclear or radiological threats due to terrorist action, accident or military conflict remain a priority for governments around the globe, since such wide radiation exposure can have mass-scale catastrophic consequences to the civilian and military communities [1, 2]. Retrospective physical and biological dosimetry techniques that are currently available include electron paramagnetic resonance, DNA damage profiling, thermoluminescence, dicentrics, chromosomal translocations, premature chromosomal condensation, hematology as well as metabolite and protein biomarkers [3–9]. However, these assays do not provide the required high throughput capability needed to screen a large number of individuals during any mass casualty scenario.

In this context, the molecular phenotyping technologies may be highly useful for the development of minimally invasive biomarker assays for rapid assessment of radiation exposure victims. Biomarkers could be instrumental to inform the impact of specific doses of radiation on biological systems. Such strategy may lead to precise and effective treatments for exposed individuals at risk. Hence, there is a significant need for biomarker identification, validation, and qualification that could assist in the rapid evaluation of absorbed radiation doses and the related health risks [6, 10]. Additionally, radiation biomarkers are also helpful for investigating countermeasure efficacy which can be used to rescale the drug doses from animal models to humans [11]. Using various omics platforms, several metabolites, lipids, proteins, and miRNA molecules have been studied as biomarkers for radiation exposure during the last two decades. These biomarkers have been mostly studied in murine models and have used a small range of radiation doses (RDs) [6, 12, 13], and these findings have been validated using large animal models, albeit to a limited extent [5, 14–16].

MicroRNAs (miRNAs), which are 18–30 nucleotides-long noncoding moieties, control gene regulations via degenerating mRNA and suppression of protein translation [17]. By current estimation, miRNA regulates 30% of the entire human genome [18] and thereby controls a vast spectrum of biofunctions. Recent discoveries threw light on miRNAs’ role in epigenetic activities [19] that could have chronic implications, even heritable consequences. There is potentially a feedback loop between miRNA and other epigenetic mechanisms, including DNA methylation and histone modifications [19, 20]. Such widespread regulatory activities of miRNA underlines its value as diagnostic, therapeutic, and prognostic biomarkers [21]. Measuring the expression levels of cell free circulating miRNA from plasma or serum has several logistic advantages [22]. The miRNA detection process could be rapid, namely a few hours at maximum; thus, miRNA assays could provide a significant time advantage in comparison to current technologies to detect radiation exposure [3–9, 23]. Indeed, promoting miRNAs as potential biomarkers of radiation injury has been gaining significant deliberations in recent years [24–29]. Focusing on animal model-based miRNA studies, a meta-analysis concluded that miRNA could be a promising species-independent candidate to triage irradiated subjects based on the extent of injury [30].

Recent advancements in the high throughput omics approach [31] coupled with increased application of Artificial Intelligence (AI) and Machine Learning (ML)-empowered algorithm has given an unprecedented boost towards biomarker discovery [32]. Using a similar approach, a clinical study probed 16 patients and presented a panel of three serum miRNAs to determine radiation exposure [33]. Similarly, a panel of two serum miRNAs was constructed to diagnose radiotoxicity during the course of radiotherapy [34]; in addition, a fourteen-serum miRNA panel was suggested by an independent study to aid in determining RDs for cancer patients [35]. By fitting the miRNA expression values into a logistic regression curve, the variable health risks of radiation exposure from three different sources was estimated in a mouse model [36]. Clearly, the cell free circulating miRNAs have emerged as great candidates for dose assessment. However, a general concern remains about the translational potential of *in vivo* knowledge [37].

To address this concern, the current study has been built upon two of our previous publications. In the first of the two previous publications [38], we developed an *in silico* pipeline to identify phylogenetically conserved miRNAs linked to radiation exposure [38] and this pipeline was reused in the current work. The delivered miRNAs are not only sequentially conserved between NHPs and humans, but these miRNAs would likely to display similar responses to irradiation in both NHPs and humans. The second publication of interest reported a combination of mouse [27] and NHP [39] models and a cross-species interrogation identified seven evolutionary conserved miRNAs, of which a five-miRNA panel was selected as the best candidate to predict radiation-induced fatality [28]. This result essentially justified the current approach, where we used a wide range of radiation doses and a novel in silico approach to find conserved miRNA markers.

Taking advantages from these two studies, we explored a wide range of radiation doses to determine the miRNA markers linked to total-body irradiation (TBI). We present putative markers linked to radiation dose, time since irradiation (TSI), and a panel of miRNAs that could potentially predict the risk to radiation-induced fatality (RRiF), respectively. These biomarkers are expected to be species independent since the seeding pool of miRNAs was sequentially and functionally conserved between NHPs and humans.

## Materials and methods

### Experimental design

**Fig 1:**
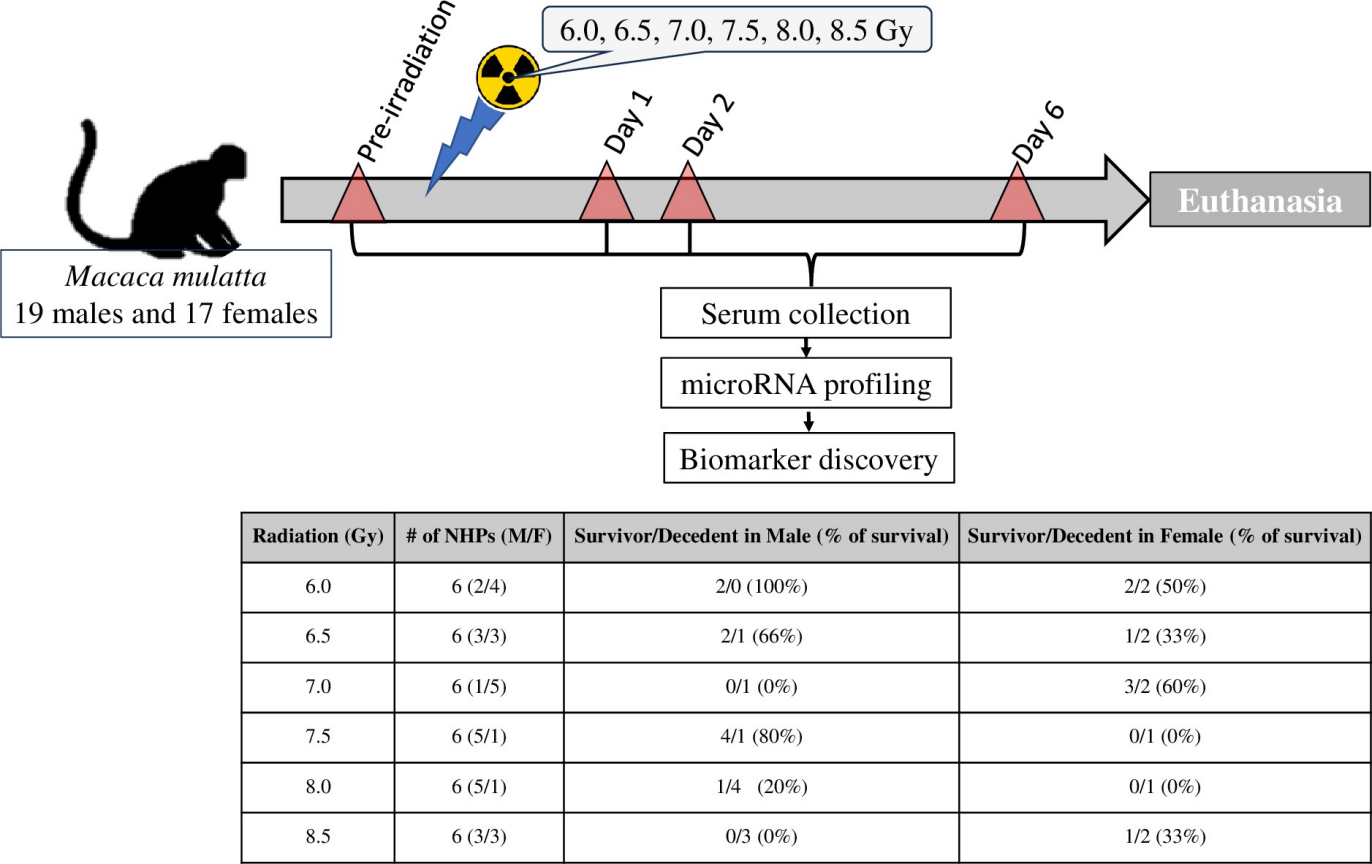
Study design. The flow diagram shows the longitudinal plan for collecting blood samples for circulating miRNA pre- and post-irradiation from NHPs. The table displays the sample size (M/F) and the survival outcome.

For this study, a total of 36 animals of both sexes were evenly distributed in six different groups. Animals in each group were exposed to one of six different ^60^Co γ-radiation doses (6.0, 6.5, 7.0, 7.5, 8.0 or 8.5 Gy) [40]. Though these animals were observed for 60 days post-irradiation to score survivors, blood samples for microRNA analysis were collected pre-irradiation and on days 1, 2 and 6 post-irradiation (Fig 1). All 21 animals that moribundity criteria for early euthanasia displayed radiation-exposure related injury.

### Animals

Naïve rhesus macaques (*Macaca mulatta*, Chinese sub-strain, 19 males and 17 females) 2.9–7.3 years of age weighing 3.6–7.4 kg, were acquired from the National Institutes of Health Animal Center (NIHAC, Poolesville, MD, USA) and maintained in a facility accredited by the Association for Assessment and Accreditation of Laboratory Animal Care (AAALAC)-International. Animals were quarantined for six weeks prior to the initiation of the study. Animal quarantine, exclusion criteria, housing, health monitoring, care, and enrichment during the experimental period have been described in detail earlier [39, 41]. In brief, all NHPs were individually housed in stainless steel cages in environmentally controlled rooms maintained at 22°C ± 2°C, 30–70% relative humidity, 10–15 air change cycles per h, and 12 h light:12 h dark cycle. Animals were fed primate diet (Harlan Teklad T.2050 diet, Madison, WI, USA) twice daily and they received drinking water *ad libitum*. All the animals received enrichment food once a day Monday–Friday. They received mirrors, toys, and challenge balls for enrichment. TVs were used for sensory enrichment for 4–5 h at least 3 times a week. All animals were able to see, hear and/or touch the conspecifics through the cages. All animals were randomly assigned to one of six different irradiation groups. All procedures involving animals were approved by the Institutional Animal Care and Use Committee (IACUC) of the Armed Forces Radiobiology Research Institute (AFRRI) Protocol #2015-12-011 and University of Maryland Baltimore Protocol #0581005, as well as the Department of Defense Animal Care and Use Review Office (ACURO). This study was carried out in strict accordance with the recommendations in the *Guide for the Care and Use of Laboratory Animals* [42]. This study used full supportive care including whole blood transfusion, although all samples for this study were collected prior to any blood transfusion to animals [16]. This study was carried out in compliance with the ARRIVE guidelines.

### Irradiation

The irradiation procedure and dosimetry have been previously described [43]. In brief, two NHPs were placed on the irradiation platform facing away from each other and were exposed to a specific midline dose of ^60^Co gamma-radiation at a dose rate of 0.6 Gy/min (bilateral, simultaneous exposure). The radiation field in the area of the NHP location was uniform within ± 1.5%. The dosimetry for photons was based on the alanine/EPR (electron paramagnetic resonance) dosimetry system [44]. Dose measurements and calibrations (EMXmicro spectrometer, Bruker Corp., Billerica, MA, USA) were based on the alanine/electron paramagnetic resonance system [44, 45]. The alanine dosimeters obtained from the National Institute of Standards and Technology (NIST, Gaithersburg, MD, USA) were calibrated using methods previously discussed [41]. Animals were mildly sedated for the procedure. To deliver the precise radiation dose, NHPs’ abdominal widths were measured with digital calipers. Animals were observed throughout the irradiation procedure via in-room cameras [46]. Following irradiation, animals were returned to the transport cart and brought to their cages in the housing area and were closely monitored until they were able to perch within their cage.

### Cage-side animal observations

All NHPs were observed pre-irradiation and for 60 d post-irradiation with survival as the primary endpoint. Daily observations for signs of pain and distress were made no less than twice a day by trained staff for executing NHP procedures and observation. During the critical period (approximately 10 to 20 d post-irradiation), animals were observed three times a day for signs of radiation sickness or need for further medical intervention. The animals were evaluated for the following parameters at least three times a week: weight, body temperature, fecal consistency, respiratory rate, heart rate, and overall health assessment [39].

### Euthanasia

Euthanasia was conducted in accordance with the most recently approved versions of the IACUC protocol, the *Guide*, and American Veterinary Medical Association (AVMA) guidelines [42, 47]. When an animal reached a state of moribundity, the animal was immediately euthanized. Moribundity was used as a surrogate for mortality, and animals were euthanized in order to minimize pain and distress [39]. Various parameters were used as guidelines for moribundity, which have been previously listed [39]. Moribundity status of each animal was determined by a joint effort between the institutional veterinarian, principal investigator, research staff, veterinary technicians, and husbandry staff based on the combination of criteria described above. To initiate euthanasia, the animals were sedated using ketamine hydrochloride injection (Mylan Institutional LLC, Rockford, IL) (5–15 mg/kg, *im*), and then administered pentobarbital sodium *iv* (Virbac AH Inc., Fort Worth, TX) using either saphenous or cephalic veins, needle size 20–25 gauge (100 mg/kg, 1–5 ml). Intra-cardiac administration was performed if unable to administer pentobarbital sodium through peripheral veins. The animals were deeply anesthetized by Isoflurane (Baxter Healthcare Corporation, Deerfield, IL) (1–5%) with oxygen at 1–4 liters per minute via mask before administering the intra-cardiac injection. The animals were euthanized only under the guidance of a staff veterinarian or a trained technician in consultation with the veterinarian. After pentobarbital sodium administration, the animals were examined by assessing the heart auscultation and pulse to confirm death. At the end of the study (60 d post-irradiation), all surviving animals were sent to another facility for studying late and delayed effects of radiation exposure.

### Blood collection and serum separation

On the day of a scheduled sample collection, animals were restrained using the pole-and-collar method and were guided to a restraint chair for blood collection. The blood draw was conducted between 08:00 AM and 10:00 AM, 1–3 h after animals were fed. Blood samples were collected from the saphenous vein of the lower leg after the site was cleaned using a 70% isopropyl alcohol wipe and dried with sterile gauze [48]. A 3 ml disposable luer-lock syringe with a 25-gauge needle was used to collect the desired volume of blood. For serum collection, blood samples were transferred to vacutainer serum separator tubes (Becton, Dickinson Corp, Franklin Lakes, NJ, USA), allowed to clot for 30 min, and then centrifuged for 10 minutes at 400 x g. Serum samples were then stored at -70°C for analysis at a later date [49]. As stated above, for miRNA analysis, samples were collected on days -4 pre-irradiation and days 1, 2, and 6 post-irradiation.

### miRNA assay

The assay protocol that we have reported elsewhere [50] was adapted herein with necessary modifications. We used TruSeq small RNA sample preparation kit to construct the sequencing library from 5 μL of NHP serum samples, as the 3′ and 5′ adapters were ligated to small RNA molecules, and the ligated products were reverse transcribed and amplified. The sample library was gel purified and size selected to curate small RNAs that were less than 30 bases; subsequently, the library was quantified by TapeStation (QIAGEN, Inc.). Based on the sample concentrations, equimolar amounts of small RNA-derived libraries were processed in Illumina NextSeq 500 platform to generate 5 million reads for miRNA profiling [29]. Illumina-generated Base Call (BCL) files were converted to FastQ files and electronically demultiplexed using bcl2fastq2 v2.20 software (Illumina Inc., San Diego, CA, USA). Genomics Workbench 20.0.4 software (QIAGEN, Germany) was used for analysis of the samples and to enumerate the miRNA expression values. FastQ file reads were first filtered to reject the poor reads then the screened reads from different lanes were conjoined. The trimming was performed using a score of 0.05 with the maximum number of ambiguities equaled to 2. We used a custom generated trim adaptor list containing an Illumina small adapter, as the allowed length of sequences were between 19 and 25 with a maximum mismatch of 2. Then, miRNA expression counts were determined using miRbase Release 22.1 with reference for NHP gene assembly. Read counts of mature miRNA were then normalized to CPM values using the trimmed mean of the M values (TMM) normalization method. Mature miRNA expression values were then subjected to differential analysis.

The TMM normalized data was first prefiltered to remove miRNAs with < 40% non-zero reads across all samples. Differential expression (DE) analysis was then performed by calculating the logfold2 change values using the pre-irradiation samples as the baseline. Preliminary analysis used four major co-factors of this study: (i) RDs (6.0 Gy, 6.5 Gy, 7.0 Gy, 7.5 Gy, 8.0 Gy and 8.5 Gy); (ii) TSI, namely pre-irradiation, day 1, day 2 and day 6 post-irradiation, respectively; (iii) male and female sex; and (iv) RRiF, or animals that survived until 45 days post-irradiation were reported as *survived*.

Four-way ANOVA was computed to find differentially expressed miRNAs linked to 15 dependent and independent variables at the cut-off of *p*<0.05. The list included four independent variables, namely RD, TSI, Sex and RRiF, and the corresponding 11 dependent variables, where multiple variables were connected via *; the list was the following: RD*TSI, RD*Sex, RD*RRiF, TSI*Sex, TSI*RRiF, Sex* RRiF, RD*TSI* RRiF, RD*Sex* RRiF, RD*TSI* RRiF, TSI*Sex* RRiF and RD*TSI*Sex* RRiF.

We used the RNASeqPower Bioconductor package [51] to determine whether the sample size of 36 was sufficient to detect the differences corresponding to all of the multiple variables listed above. The current assay is expected to generate 400 million reads (length 50 bases per read) on a NextEeq500 platform. Given that NHP have nearly 3,100 million bps in its whole reference genome, we assumed to meet a 6.45x coverage in our assay. To measure the statistical power for the ANOVA analysis, the effect sizes (η^2^) of all combinations of variables were computed, and the mean effect size and its coefficient of variation (CV) was determined as 0.21, and 1.7, respectively. With an alpha value at 0.05, the power of the study was expected to be greater than 80%, which was sufficient to proceed with further analysis.

**Fig 2:**
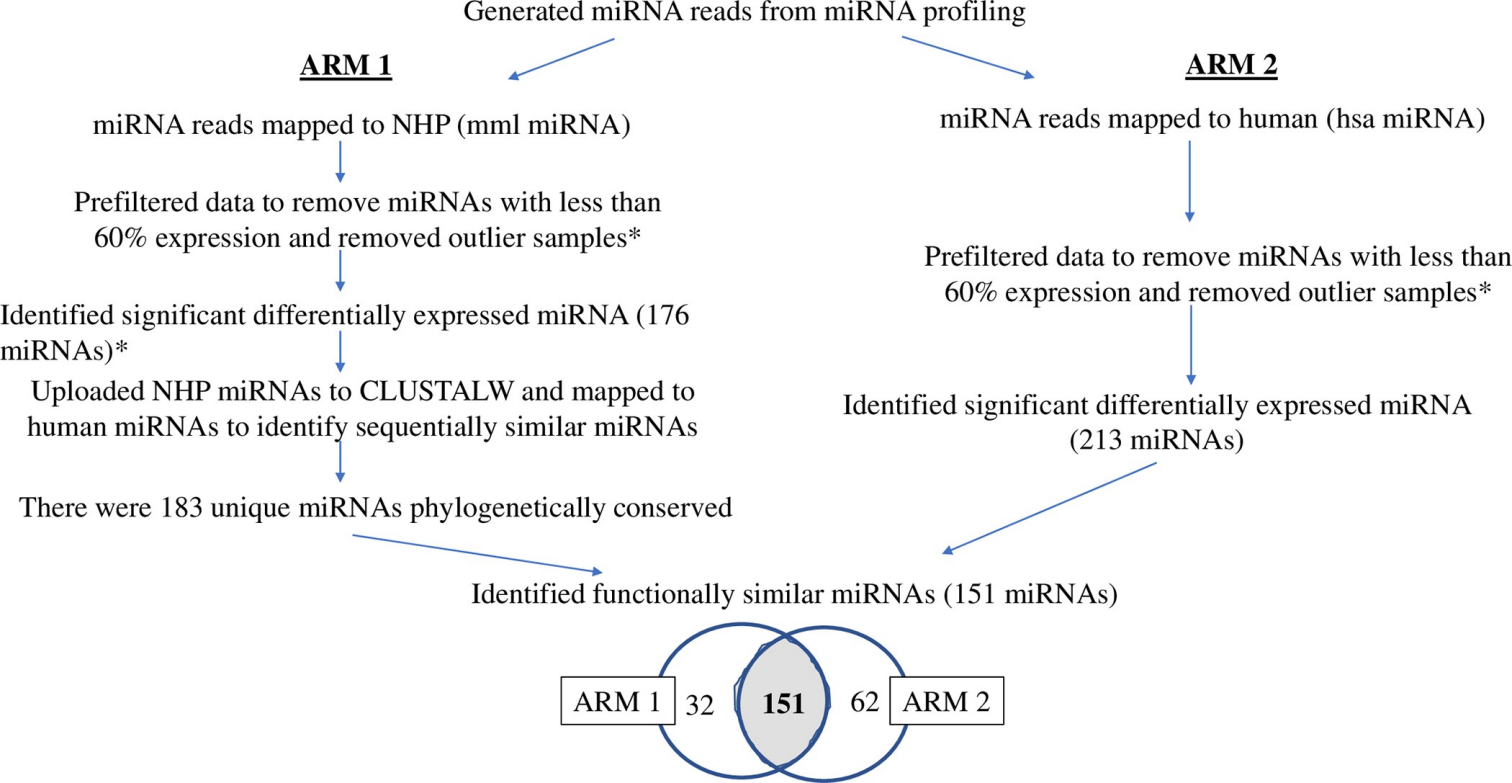
A flow chart to depict the process to deliver miRNAs that were not only sequential homologues between humans and NHPs, but were also likely to respond similarly to irradiation across these two species.

Subsequent analysis was focused on curating those DE mml-miRNAs, which would be sequentially conserved and functionally similar between humans and NHPs exposed to TBI (Fig 2). We described this process elsewhere in the context of a minipig model [38]. Briefly, we pooled the DE mml-miRNAs that were selected by 4-way ANOVA, namely 176 miRNAs after removing the duplicates. These miRNAs were seeded into the multiple-sequence alignment tool, ClustlW [52], to curate sequentially conserved miRNAs between humans and NHPs. FASTA sequences of all mature miRNA sequences were retrieved from miRBase. A pairwise sequence alignment between DE mml-miRNAs and the retrieved human miRNAs was conducted with specified loci positions to meet the following parameters: the k-tuple word size 1 and window size 5 with top diagonals of 5 with percent method. Gap penalty of 10 and penalty of extension 0.1 with a BLOSUM scoring weight matrix were applied. Scores were calculated using the absolute and percent method and the top match(es) for each NHP miRNA was determined. As a result, we identified a set of mml-miRNAs, which were sequential homologues to the known human miRNAs. We named this set of miRNAs as sequentially conserved-miRNAor *sc*-miRNA.

In parallel, we employed miRBase to align the quality filtered and adaptor-trimmed reads to the human genome assembly, hg38. Thereby, we followed the previously described analysis pipeline with one important difference, namely the miRDeep2 core algorithm in miRBase curated known hsa-miRNAs, not mml-miRNA. The resultant hsa-miRNA matrix was quality filtered and the counts were normalized to generate hsa-miRNA sets. Four-way ANOVA with the cut-off set at *p* < 0.05 found a set of miRNAs that met the cut-off in at least one of the 15 feature selection criteria as mentioned earlier. We named this set of hsa-miRNAs as functionally similar-miRNA or *fs*-miRNA. A subsequent Venn approach identified those miRNAs that were conserved between *sc*-miRNA and *fs*-miRNA. We named these overlapping features as conserved-miRNA (*cnvd*-miRNA). Henceforth, all statistical analysis was computed using this *cnvd*-miRNAs. The raw data is saved in GEO database, GSE247225.

### Statistical analysis

R Studio was used to generate principal component analysis (PCA) plots and carry out hierarchical clustering computed by Euclidian distance analysis using heatmap2 function from the gplots. The time and dose dependent miRNA discovery was computed using GraphPad v8 (Prism, Inc.). A linear regression plot was drawn to measure if the slope significantly deviates from zero and the cut-off was set at *p* < 0.05.

Ingenuity Pathway Analysis (IPA, QIAGEN, Inc., version 01–13) was used for functional analysis and bionetwork building. Differentially expressed miRNAs of the following five groups were of particular interest: (i) RD*TSI, (ii) Sex*RD*TSI, (iii) RRiF, (iv) RRiF*RD, and (v) Sex*RRiF*RD. DE miRNAs from these groups were transferred to the IPA analysis portal, and the canonical and non-canonical bionetworks that were significantly enriched at each of the five groups were curated (hypergeometric test, *p* <0.05). Henceforth, all the canonical and noncanonical bionetworks were together termed as bionetworks. Those bionetworks that displayed a Z score of greater than 1 (≥1) were considered activated bionetworks. In contrast, those bionetworks, which displayed Z scores of less than 1 (≤ -1) were considered inhibited bionetworks. In any given group, if a bionetwork’s Z score was greater than |1| in at least one instance, that bionetwork was curated for further interrogation. The correlation matrix was computed using the Pearson correlation formula.

**Fig 3:**
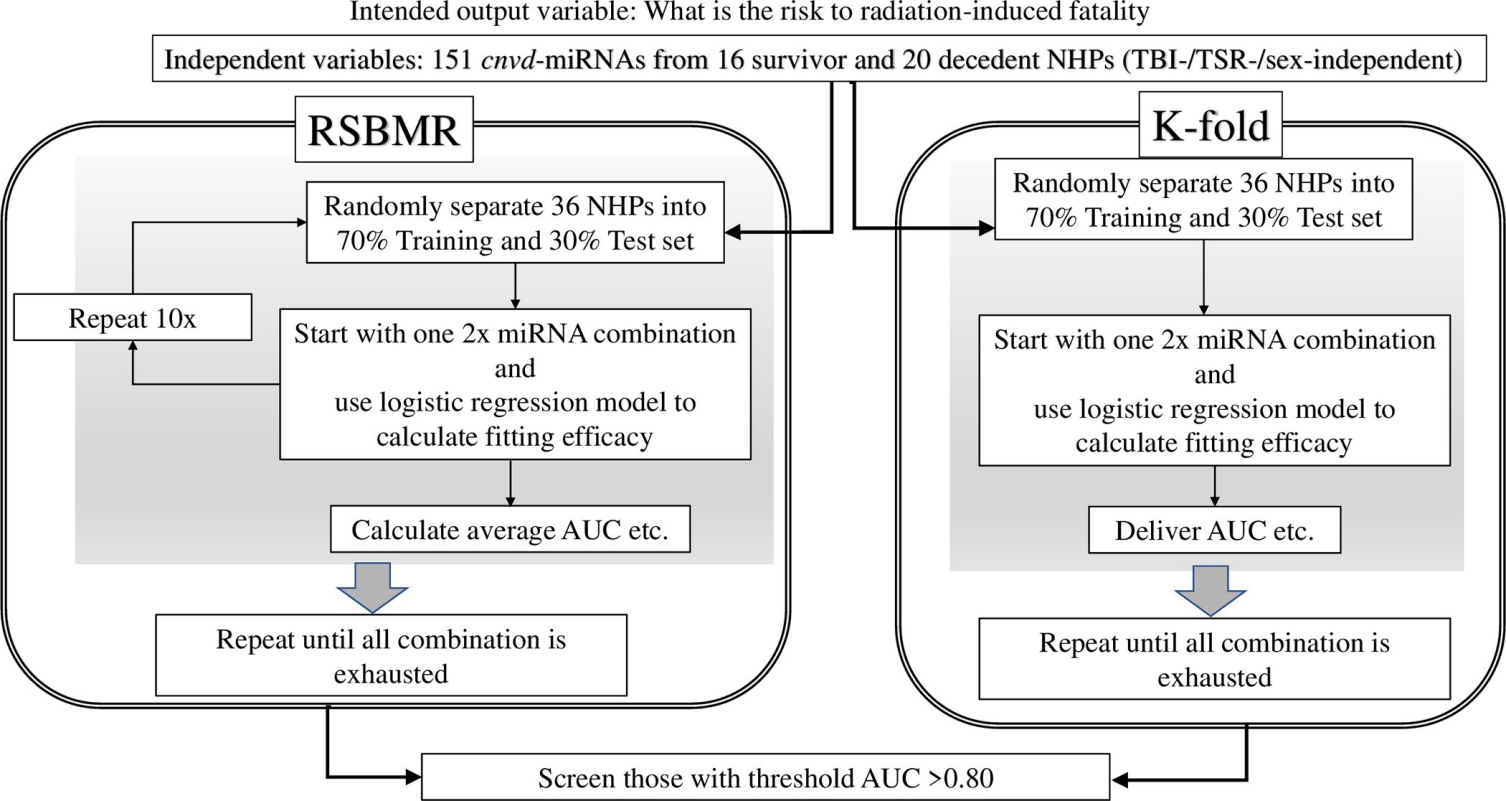
A flow diagram to explain the algorithm named 2BDP (Biomarker Discovery Process at Binomial Decision Point) that we used to find the best fitting model to predict RRiF.


To discover the panel of miRNAs that can potentially predict RRiF, we used our proprietary machine learning routine named the Biomarker Discovery Process at Binomial Decision Point (2BDP) [53], which is trained to systematically incorporate independent variables (e.g., multiple DE miRNAs) to explain an output variable (e.g., RRiF) that is binary in nature. As depicted in Fig 3, 2BDP operated on the *cnvd*-miRNAs via an exhaustive search to generate the best fitting logistic regression model as described in Eq 1.

$$logit(P)=a+bX_{1}+cX_{2}+\cdots +nX_{n}$$ (Eq 1)

Where logit() is the log odds function of a value, *P* is the probability of successful determination of the outcome variable, *a* is the intercept of the equation, *b* through *n* are coefficient estimates of the independent variables, and *X*_*1*_ through *X*_*n*_ are the expression values of the independent variables *1* to *n*, respectively. The fitting criteria of these independent variables were measured by multiple R^2^, adjusted R^2^, confidence intervals (CIs) and p values (Chi-square). For the present purpose, the fitting models allowed the independent variables to change from 2 to 10 (2≤ *n* ≤ 10) per panel.

Thereby, 2BDP algorithm measured the efficacy of the individual biomarker panel in determining the outcome variables or RRiF. The panel contained a group of independent features that were linearly associated via unique weight factors or coefficients. The efficacy of the individual panel was quantitively measured by the area under the curve (AUC) of the receiver operating characteristic (ROC) curve. We used two methods to measure the ROC curve.

Method 1. Random Single Bin Multiple Repeats (RSBMR): The independent features from *cnvd*-miRNAs were used in a random forest 2000 times, where the top 200 panels of most frequent genes were selected. For each panel, every combination of genes, from 2 to 10 feature sized panels, constructed a series of unique subpanels. Hence, the maximum count of features in one panel should not exceed 10. Next, the whole NHP cohort all-inclusive on sex, RD and TSI was randomly sorted into Training and Test set by the 70:30 ratio. A unique panel of independent features was fitted onto the Training set to construct a linear regression model as shown in Eq 1. Afterwards, this linear regression model was operated on the Test set to calculate the AUC and sensitivity/specificity. These deliverables were retained while the exhausted Training and Test sets were replaced by new Test and Training sets. This cycle was repeated 10 times for each subpanel and finally, the mean values of AUCs and sensitivity/specificity were calculated over these 10 iterations and were then reported. This iterative process continued until all features in *cnvd*-miRNAs were exhausted.

Method 2. k-fold method: The independent features from *cnvd*-miRNAs were used in a random forest 2000 times, where the top 200 panels of most frequent genes were selected. For each panel, every combination of genes, from 2 to 10 feature sized panels, constructed a series of unique subpanels. For any given unique panel, the entire cohort was segregated into 10 (k = 10) groups, and one randomly selected group was chosen as the Test set. The remaining groups were taken as Training sets, where the unique panel was fitted to construct a linear regression model as shown in Eq 1. Next, the AUC was calculated using the Test set and was reported. This iterative process continued until all features in *cnvd*-miRNAs were exhausted. Finally, all panels curated by RSBMR and k-fold methods were screened to find those which had a significantly high fitting score, *p* < 0.05 and (b) AUC > 0.80, respectively.

## Results

Thirty-six NHPs were randomly assigned to six groups and each group was exposed to different RDs starting from 6.0 Gy to 8.5 Gy in 0.5 Gy increments (Fig 1). Each group consisted of 6 animals with variable male to female ratios. A table attached to Fig 1 displays the number of animals that survived from each radiation dose.

### Cross-species conserved miRNA

Sequencing reads were mapped to 3,127 known miRNAs in the NHP genome assembly, and a total of 1,327 known miRNAs were detected in at least one of the 144 samples. On average, 364.6 ± 0.46 miRNAs were detected per sample, where the error was calculated by standard error of the mean (SEM). After filtering out the features with more than 60% samples, 1,040 miRNAs became eligible for multivariance analysis. S1 Fig depicts the PCA plots of these miRNAs. The plot explained 22.09% of total variance, but no apparent trend emerged in this plot. S1A Table lists the number of DE miRNAs curated by 15 dependent and independent variables. We combined all these miRNAs and removed the duplicates down to select 176 miRNAs (S1B Table), which were significantly altered in at least one of the 15 comparative analyses computed by the 4-way ANOVA model. These 176 miRNAs were further processed through multiple sequence alignments between human and NHP miRNA assembly to deliver miRNAs of high translational potential (Fig 2). Briefly, we first identified 183 miRNAs that are sequential homologues between humans and NHPs using a multiple sequence alignment routine [52] and named this miRNA panel as sequentially conserved-miRNA (*sc*-miRNA). In a parallel pipeline, the quality controlled NextSeq reads were *directly* blasted to human genome assembly, and differential expression analysis was performed to curate 213 significantly different hsa-miRNAs from the pre-TBI baseline. We labeled this second set as *fs*-miRNA. Ultimately, 183 *sc*-miRNAs and 213 *fs*-miRNAs were overlapped to find 151 miRNAs that were common between these two sets, which was named *cnvd*-miRNA (S1C Table). Subsequent analysis of this study used this *cnvd*-miRNA panel, since we posit that these miRNAs are not only sequentially conserved between humans and NHPs, but also that their functional response to TBI and TSI are expected to be similar. Hence, these *cnvd*-miRNAs had considerable translational potential.

### miRNAs markers showing linear response to dose and time

**Fig 4:**
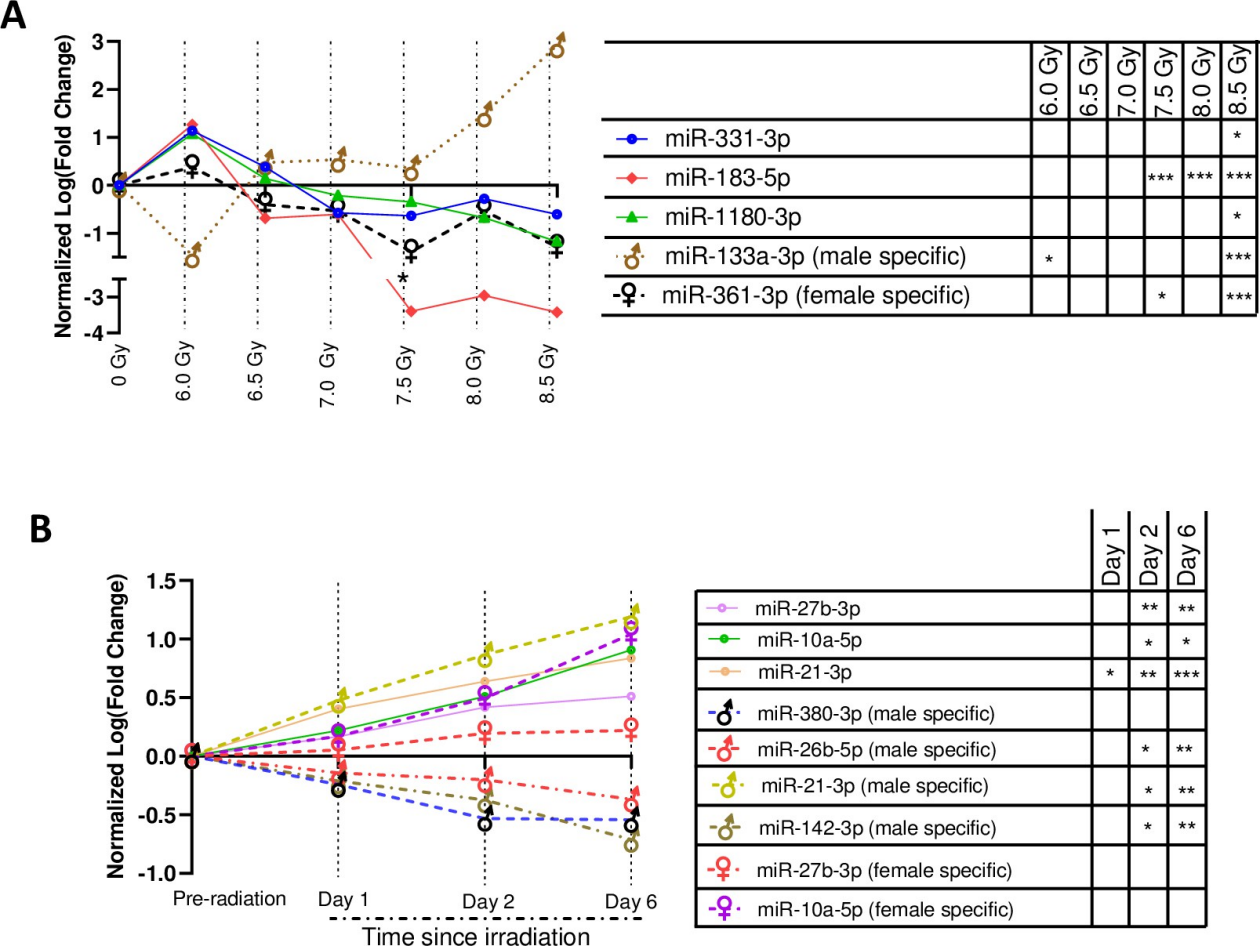
Putative miRNA markers of irradiation. All miRNAs sequentially and functionally conserved between humans and NHPs. **(A)** Time since irradiation (TSI)-independent, radiation dose (RD)-miRNA markers. Here, the x-axis and y-axis represented the radiation dose and normalized log2 (Fold change), respectively. The fold changes were computed on the pre-irradiation baseline controls. All these miRNAs displayed a consistent shift (*p* < 0.05 in linear regression curve) across dosimetry independent of TSI. * *p* < 0.05; *** *p* < 0.001. **(B)** RD-independent, TSI-miRNA markers. Here, the x-axis and y-axis represented the TSI and normalized log2 (Fold change), respectively. The fold changes were computed on the pre-irradiation baseline controls. All these miRNAs displayed consistent shift (*p* < 0.05 in linear regression curve) across TSI. * *p*<0.05; ** *p*<0.05; *** *p*<0.001.

A linear regression model-based screening of 151 *cnvd*-miRNAs curated five miRNAs showing consistent and significant shifts across doses, but independent of time (Fig 4A). The features of Fig 4A was curated from the sex*RD ANOVA analysis, where TSI was precluded as a dependent variable. Three of these 5 miRNAs, namely miR-331-3p (F = 7.26, *p* = 0.04), miR-183-5p (F = 22.13, *p* = 0.009) and miR-1180-3p (F = 52.7, *p* = 0.001) emerged as sex-independent markers, as they gradually decreased with increasing dose. Here, the F values and *p*-values characterized the deviations of their slopes across doses from baseline. One miRNA, specifically miR-183-5p, was significantly inhibited from the baseline at the three highest doses of radiation (namely 7.5 Gy, 8.0 Gy and 8.5 Gy), and displayed significant separation of the entire dose range with an AUC ROC curve of 0.95. Its 95% confidence interval (CI) ranged from 0.84 to 1.00. Another miRNA, miR-133a (F = 11.42, *p* = 0.002), was a male-specific marker that significantly increased with increasing RD; contrastingly, miR-361-3p (F = 9.52, *p* = 0.027) was a female-specific marker that significantly decreased with increasing RD. All 5 miRNAs were significantly altered from the pre-irradiation baseline at 8.5 Gy, irrespective of TSI. However, none of these miRNAs showed a significant shift from the baseline at the lowest dose, namely 6.0 Gy.

Fig 4B displays 11 miRNA that consistently shifted across time or TSI. These features of Fig 4B was curated from the sex*TSYI ANOVA analysis, where RD was precluded as a dependent variable. There were 3 TSI-markers, namely miR-27b-3p (F = 74.62, *p* = 0.013), miR-21-3p (F = 66.84, *p* = 0.015) and miR-10a-3p (F = 11.9, *p* = 0.009), that were significantly upregulated with increasing time. One miRNA, miR-21-3p, was significantly altered from the baseline across the entire TSI profile and displayed a separation of the group with an AUC of 0.72, and its 95% CI ranged from 0.33 to 1.00. In addition, there were 5 male specific and 3 female specific miRNAs that consistently shifted their regulations with increasing time. To note, miR-21-3p emerged as both a gender-inclusive and male-exclusive marker. Indeed, miR-21-3p (F = 281.6, *p* = 0.003) was the only candidate that was significantly upregulated starting from day 1 post-TBI. Three candidates including miR-26b-5p (F = 68.38, *p* = 0.014), miR-125b-2-3p (F = 69.21, *p* = 0.014) and miR-142-3p (F = 80.68, *p* = 0.122) were consistently downregulated with increasing time, while the remaining were increasingly upregulated with time.

### RD- and TSI-specific bionetworks

**Fig 5:**
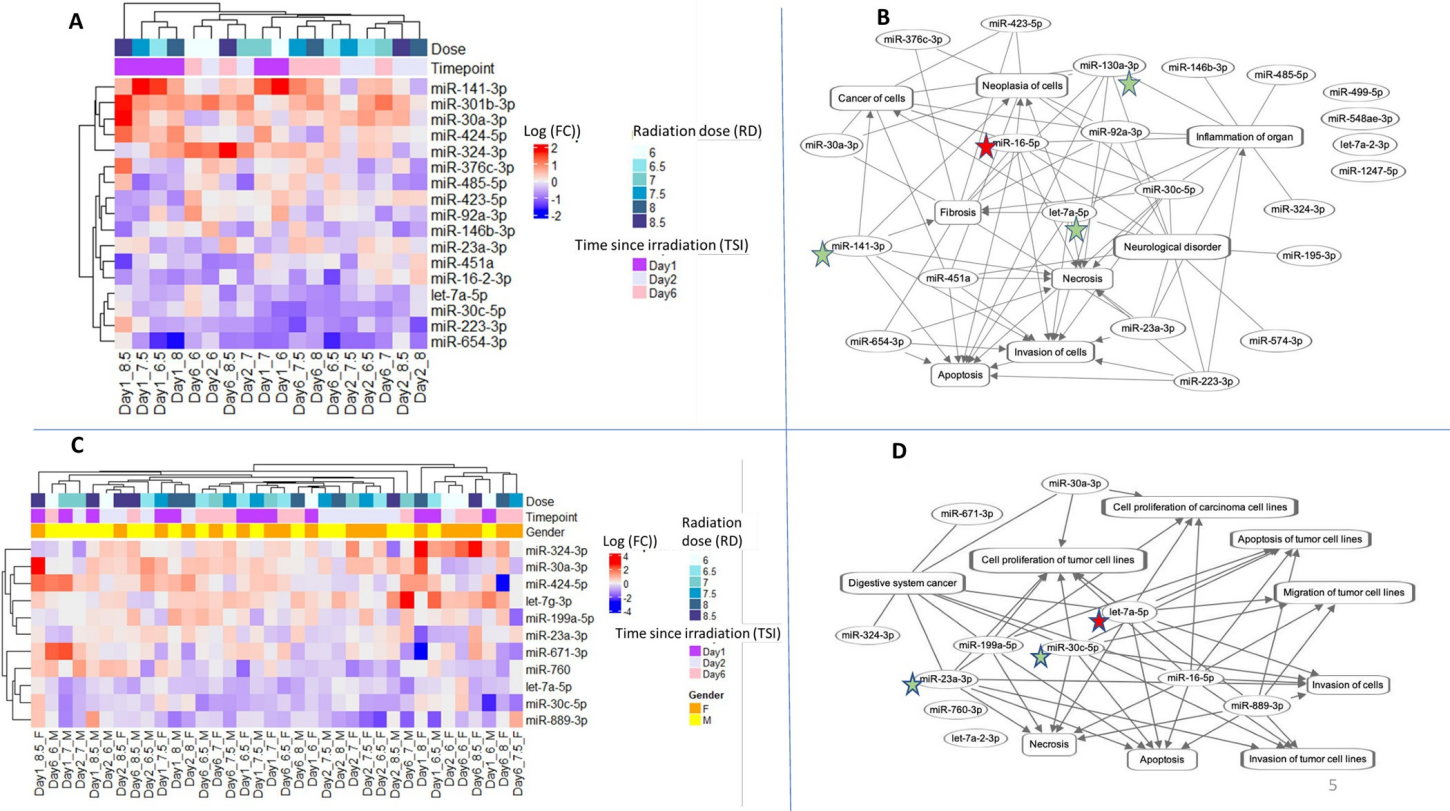
Functional association among the differentially expressed miRNA cluster linked to RD and TSI. The color keys of log2 (Fold change) were added at top left corners. In the networks, the oval and rectangular shaped nodes represent miRNA and biofunctions, respectively. The edges represent the relationship between two nodes: the solid lines represent their association, and pointed arrowheads denote the activating relationship between the two nodes. Of these, miRNAs that appeared most connected to the neighbors were annotated in the red colored stars for > 90% percentile, and green colored stars for > 75% percentile. **(A)** Hierarchical cluster of 17 miRNAs that were differentially expressed by the cumulative effects of RD and TSI. These miRNAs are sex-independent markers. **(B)** Network cluster of the same 17 miRNAs that were differentially expressed by RD*TSI. **(C)** Hierarchical cluster of 11 miRNAs that were differentially expressed by the cumulative effects of Sex, RD and TSI. **(D)** Network cluster of the same 11 miRNAs that were differentially expressed by Sex*RD*TSI.

There were 17 miRNAs that were significantly altered by RD*TSI; furthermore, these 17 miRNAs were a subset of *cnvd*-miRNAs as described in Fig 2. Fig 5A depicted a hierarchical cluster of these 17 miRNAs, and Fig 5B displayed a functional network using these 17 miRNAs to reveal their biological interrelationships. One miRNA, miR-165p, emerged as the most interconnected candidate, followed by miR-130a-3p, miR-7a-3p, and miR-141-3p. Together, these four miRNAs could be a set hub of molecules or key regulators of the functions associated with RD*TSI.

Functional enrichment analysis (Tables 1 and 2) found 11 bionetworks that were significantly regulated due to the dependent effects of RD*TSI; of which the bionetwork linked to invasion of tumor cells was the most enriched function. Bionetworks linked to apoptosis and cell migration emerged as other key candidates that were perturbed by RD*TSI. S2 Table lists the miRNAs linked to all 3 of these bionetworks.

**Table 1:** List of bionetworks enriched by a subset of *cnvd*-miRNA: Bionetworks significantly altered by the cumulative effects of RD and TSI (RD*TSI).

	Day 1						Day 2						Day 6					
Diseases and Bio Functions	6.0 Gy	6.5 Gy	7.0 Gy	7.5 Gy	8.0 Gy	8.5 Gy	6.0 Gy	6.5 Gy	7.0 Gy	7.5 Gy	8.0 Gy	8.5 Gy	6.0 Gy	6.5 Gy	7.0 Gy	7.5 Gy	8.0 Gy	8.5 Gy
Invasion of tumor cell lines	-1.04	-0.47	-1.04	1.5	0.39	-0.33	1.16	0.64	2.65	0.28	-1.04	-0.81	-1.96	-1.16	0.64	2.65	1.79	1.88
Invasion of cells	-0.56	-0.83	-1.35	1.77	0.75	-0.7	1.46	0.98	2.83	-0.14	-0.56	-1.14	-1.4	-0.67	0.19	2.04	1.25	2.12
Apoptosis of tumor cell lines	1.43	0.66	0.66	-0.2	1.14	0.18	-0.09	0.66	-1.24	-1.82	1.43	0.66	2.01	1.43	-0.11	-2.01	-1.14	-0.47
Migration of tumor cell lines	0.22	1.21	1.12	1.03	-0.4	-0.76	-1.39	1.03	0.05	1.66	0.22	2.01	0.31	0.58	1.93	0.4	0.4	0.05
Cell proliferation of tumor cell lines	-1.12	0.47	-0.81	0.13	0.22	-0.73	0.2	-0.49	1.4	1.14	-1.12	0.5	-1.04	-0.73	0.23	1.71	1.09	1.61
Apoptosis	0.94	0.94	0.2	0.67	1.4	-0.07	0.35	1.5	-0.2	-2.05	0.94	1.5	1.68	1.68	0.76	-0.94	-0.11	0.53
Migration of cells	0.37	1.34	1.25	0.63	-0.82	-1.14	-0.99	0.63	-0.06	2.02	0.37	2.1	0.37	0.63	2.02	0.82	0.31	0.46
Cell viability of tumor cell lines	-1.39	-1.39	-1.39	-0.28	-1.94	-0.83	0.28	-0.28	0.83	0.83	-1.39	-0.28	-1.94	-0.83	-0.28	0.83	0.83	-0.28
Cell proliferation of colorectal cancer cell lines	-0.18	0.43	-0.93	0.43	-0.58	-0.58	0.18	0.43	1.18	-0.18	-0.18	0.43	0.18	-0.58	0.43	1.18	1.18	1.18
Metastasis	-0.19	0.75	0.75	-0.19	-0.19	0.75	0.75	-0.19	0.75	0.19	-0.19	-0.75	-0.19	-1.69	0.75	1.69	1.69	0.75
Cell proliferation of carcinoma cell lines	-0.63	-0.63	-0.63	-0.16	-0.16	-1.41	0.63	-0.16	0.63	0.16	-0.63	0.63	-0.63	-0.94	0.63	0.63	0.63	0.63

Bionetworks with a Z score of ≥ 1 and ≤ -1 were considered activated and inhibited networks, respectively. The tables included those bionetworks, which scored ≥ |1| in at least one of all tested conditions.

**Table 2:** List of bionetworks enriched by a subset of *cnvd*-miRNA: Bionetworks significantly altered by the cumulative effects of sex, RD and TSI (sex*RD*TSI).

**Male**																		
	**6.0 Gy**			**6.5 Gy**			**7.0 Gy**			**7.5 Gy**			**8.0 Gy**			**8.5 Gy**		
**Diseases and Bio Functions**	**Day 1**	**Day 2**	**Day 6**	**Day 1**	**Day 2**	**Day 6**	**Day 1**	**Day 2**	**Day 6**	**Day 1**	**Day 2**	**Day 6**	**Day 1**	**Day 2**	**Day 6**	**Day 1**	**Day 2**	**Day 6**
Apoptosis	1.20	-0.57	0.57	-0.27	1.27	-0.59	-0.88	0.37	0.35	1.37	-1.26	0.21	0.25	-0.25	-0.63	0.74	-0.74	-0.59
Apoptosis of tumor cell lines	0.62	-0.62	0.62	0.41	0.68	-1.10	-0.77	0.30	-0.19	0.78	-1.29	-0.48	0.31	-0.31	-1.29	0.78	-1.39	-1.10
Cell proliferation of carcinoma cell lines	-0.76	1.07	-1.07	-1.07	-0.76	0.15	N/A	1.99	0.52	0.15	0.76	0.15	0.15	0.76	1.07	-1.07	0.15	0.15
Cell proliferation of tumor cell lines	-0.43	0.55	-0.55	-0.97	-0.39	1.25	0.52	0.22	0.42	-0.35	1.18	0.44	0.10	0.39	1.30	-0.97	1.26	1.25
Invasion of cells	1.60	-0.64	0.64	-1.24	0.64	0.70	N/A	-0.63	0.90	-0.27	0.33	2.21	0.64	-0.64	1.30	-1.24	2.21	0.70
Invasion of tumor cell lines	1.29	-0.20	0.20	-0.88	0.20	1.30	0.20	-0.20	1.29	0.20	0.88	1.97	0.20	-0.20	1.97	-0.88	1.97	1.29
Migration of tumor cell lines	0.65	-0.65	0.65	0.90	-0.53	1.88	N/A	-0.65	1.76	0.70	1.51	0.28	-0.33	0.33	1.51	0.70	0.28	1.88
**Female**																		
	**6.0 Gy**			**6.5 Gy**			**7.0 Gy**			**7.5 Gy**			**8.0 Gy**			**8.5 Gy**		
**Diseases and Bio Functions**	**Day 1**	**Day 2**	**Day 6**	**Day 1**	**Day 2**	**Day 6**	**Day 1**	**Day 2**	**Day 6**	**Day 1**	**Day 2**	**Day 6**	**Day 1**	**Day 2**	**Day 6**	**Day 1**	**Day 2**	**Day 6**
Apoptosis	0.42	0.21	-1.37	-0.38	0.21	0.57	-0.38	-0.74	0.36	-0.59	-1.37	1.00	0.83	-0.63	-0.25	-0.27	-1.58	0.21
Apoptosis of tumor cell lines	-0.13	0.13	-0.78	0.31	-0.48	1.23	-0.29	-0.78	-0.19	-0.50	-1.39	0.28	N/A	-1.29	-0.31	0.42	-2.20	-0.48
Cell proliferation of carcinoma cell lines	1.09	1.07	-0.15	-1.98	1.07	-1.98	-1.98	0.15	0.15	1.07	-1.07	N/A	-1.98	1.98	-0.15	-1.07	1.98	1.07
Cell proliferation of tumor cell lines	0.95	0.15	0.35	-1.01	0.93	-1.82	-0.22	0.48	0.43	0.95	0.65	0.64	-2.19	1.79	-0.10	-0.97	2.61	0.93
Invasion of cells	-0.27	1.24	0.27	-0.33	2.21	-0.33	0.64	1.24	0.70	-0.27	1.24	0.03	-0.93	1.30	-0.64	-1.24	1.30	2.21
Invasion of tumor cell lines	0.20	0.88	-0.20	-0.88	1.97	-0.88	0.20	0.88	1.29	0.20	0.88	N/A	N/A	1.97	-0.20	-0.88	1.97	1.97
Migration of tumor cell lines	0.70	-0.70	-0.70	-0.33	0.28	-0.33	0.65	-0.70	1.88	0.90	0.28	N/A	N/A	1.51	0.33	0.90	1.51	0.28

Bionetworks with a Z score of ≥ 1 and ≤ -1 were considered activated and inhibited networks, respectively. The tables included those bionetworks, which scored ≥ |1| in at least one of all tested conditions.

A correlation matrix was computed to comprehend the degree of coregulation among the 11 bionetworks that were perturbed by RD*TSI (S3A Table). In general, the cell invasion-related bionetworks were positively correlated with the bionetworks linked to proliferation and metastasis, while negatively correlated with bionetworks related to apoptosis. Bionetworks linked to invasion and proliferation were activated at 6-day after the exposure to three highest doses of this study including 7.5–8.5 Gy. In contrast, these bionetworks remained inactivated 6 days post-irradiation to the two lowest doses of this study including 6.0–6.5 Gy. A completely opposite picture emerged for the bionetworks linked to apoptosis. In general, the correlation profile among the networks was most significant at 6 days post-irradiation (S3B Table).

### Sex-, RD- and TSI-sensitive bionetworks

We further probed a list of 11 miRNAs that emerged as significantly altered due to Sex*RD*TSI. As noted earlier, these 11 miRNAs were a subset of *cnvd*-miRNAs. Fig 5C depicted a hierarchical cluster of these 11 miRNAs, and Fig 5D displayed a functional network using these 11 miRNAs to reveal their biological interrelationships. Six of these 11 miRNAs perturbed by Sex*RD*TSI overlapped with the 17 miRNAs perturbed by RD*TSI (S2A Fig). Of these miRNAs, let-7a-5p emerged as the most interconnected feature as shown in Fig 5D, followed by miR-30c-5p and miR-23a-3p. Together, these three miRNAs could be the hub molecules or key regulators of the functions associated with Sex*RD*TSI.

Table 2 lists 7 bionetworks that were differentially enriched by male and female cohorts across the RD*TSI profile. Bionetworks linked to apoptosis, cell invasion and migration again emerged as the most perturbed bionetwork following the trend associated with RD*TSI. Nevertheless, a correlation matrix (S1C Table) suggested sex-dependent bias in these bionetworks’ regulation profile. For instance, the negative correlation profile at 6.0 Gy (-0.24) between males and females became positive (+0.54) at 8.5 Gy. Maximum sex-biased differences in bionetworks’ regulation profile were observed at the lower doses, while a majority of the bionetworks were positively co-regulated between males and females at 8.5 Gy TBI.

### miRNAs and corresponding bionetworks linked to risk to radiation-induced fatality (RRiF)

As expected RRiF decreased with increasing radiation doses (Fig 1). Among the cohort of 18 NHPs that were exposed to 7.0 Gy or lower, 10 NHPs survived with a 56% survival rate. On the other hand, only 6 among the 18 NHPs survived from 7.5 Gy or higher.

**Fig 6:**
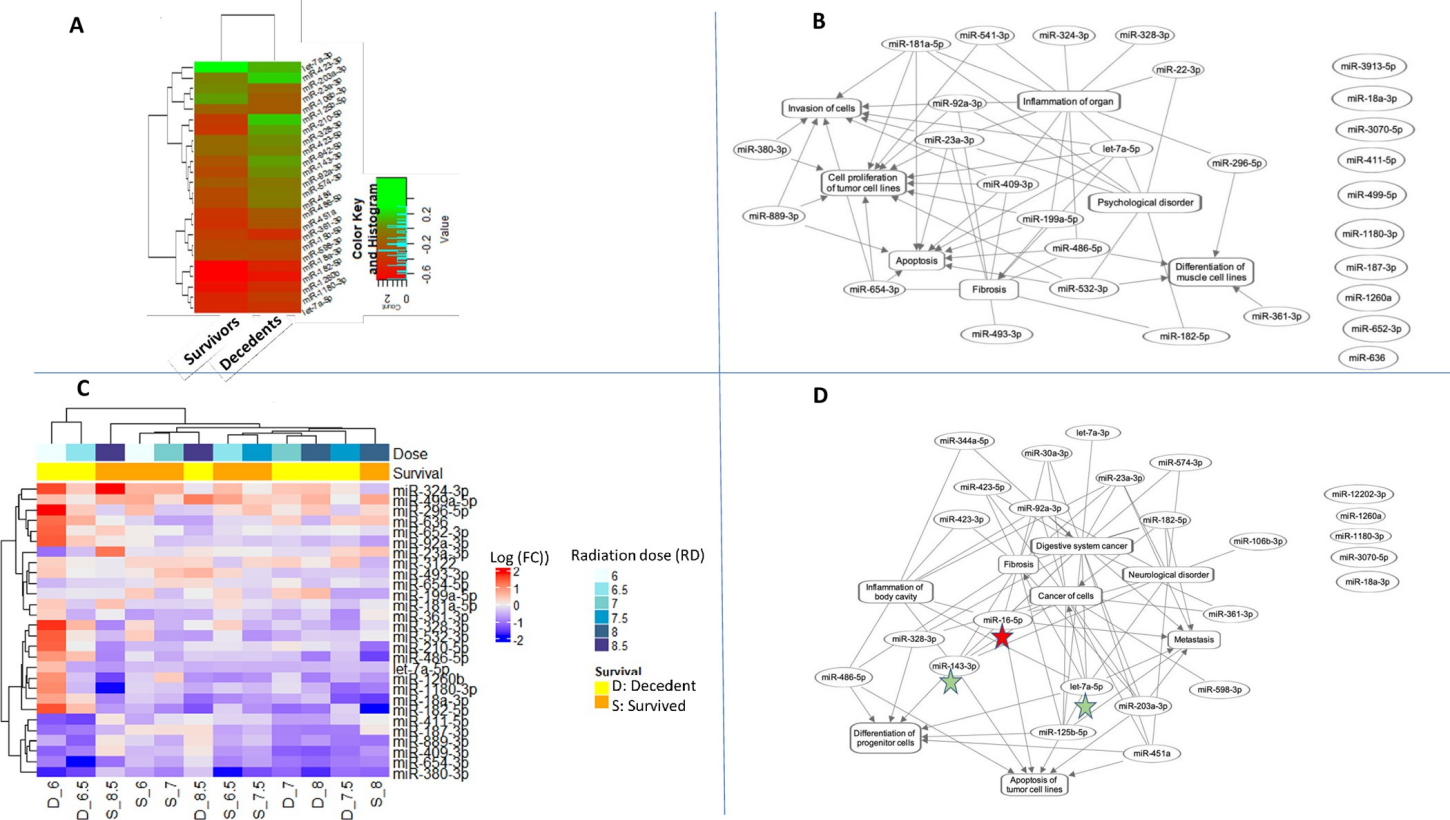
Functional association among the differentially expressed miRNA cluster linked to RRiF. The color keys of log2 (Fold change) were added at the top left corners. In these networks, the oval and rectangular shaped nodes represented miRNA and biofunctions, respectively. The edges represented the relationship between two nodes, the solid lines represented their association, and pointed arrowheads denoted the activating relationship between the two nodes. Red colored stars annotate miRNAs that were most connected to the neighbors for > 90% percentile, and green colored stars for > 75% percentile. **(A)** Hierarchical cluster of 21 differentially expressed miRNAs linked to RRiF. **(B)** Network cluster of the same 21 differentially expressed miRNAs linked to RRiF. **(C)** Hierarchical cluster of 28 differentially expressed miRNAs linked to the cumulative effects of radiation dose and RRiF. **(D)** Network cluster of the same 28 differentially expressed miRNAs linked to RD*RRiF.

There were 25 miRNAs that were significantly altered due to the independent variable RRiF. As previously mentioned, these 25 miRNAs were a subset of *cnvd*-miRNAs (Fig 2). Fig 6A depicted a hierarchical cluster of these 25 miRNAs, and Fig 6B displayed a functional network using these 25 miRNAs to reveal their biological interrelationships. Of these, miR-23a-3p and let-7a-5p emerged as the most interconnected features, followed by miR-92a-3p, miR-409-3p and miR-141-3p. Together, these four miRNAs could be the hub molecules or key regulators of the functions associated with RRiF. Functional enrichment analysis (Table 3) found that the activated bionetworks linked to cell quantity, particularly in muscle cells, were associated with the survivors, whereas the bionetwork linked to cell invasion was inhibited in the survivors. S2 Table lists the miRNAs linked to these bionetworks.

**Table 3:** List of bionetworks enriched by a subset of *cnvd*-miRNA: Bionetworks significantly altered by the RRiF.

Diseases and Bio Functions	Survivors	Decedents
Quantity of cells	1.96	-0.44
Quantity of muscle cell lines	1	-1
Differentiation of muscle cell lines	1.07	-0.76
Metastasis	1.09	-0.22
Differentiation of hematopoietic progenitor cells	1.15	0.07
Differentiation of progenitor cells	1.74	0.84
Cell viability	-0.34	-1.03
Neoplasia of tumor cell lines	0	1
Invasion of cells	-1.46	0.33
Invasion of tumor cell lines	-1.13	0.86

Bionetworks with a Z score of ≥ 1 and ≤ -1 were considered activated and inhibited networks, respectively. The tables included those bionetworks, which scored ≥ |1| in at least one of all tested conditions.

To understand the dose response in RRiF, 28 miRNAs were identified that were significantly altered by RD*RRiF. To note, there were 10 miRNAs that were common between the two sets that were linked to RRiF and TBI*RRiF, respectively (S2B Fig). Fig 6C depicted a hierarchical cluster of these 28 miRNAs, and Fig 6D displayed a functional network using these 28 miRNAs to reveal their biological interrelationships. One miRNA, miR-16p-5p, emerged as the most interconnected features, followed by miR-143-3p and miR-7a-5p. Together, these three miRNAs could be the hub molecules or key regulators of the functions associated with RD*RRiF. Functional enrichment analysis (Table 4) found that the inhibited bionetwork linked to the quantity of muscle cells emerged as a potential signature of fatality due to the low dose of radiation. S2 Table lists the miRNAs linked to this bionetwork.

**Table 4:** List of bionetworks enriched by a subset of *cnvd*-miRNA: Bionetworks significantly altered by the cumulative effects of RRiF and RD (RRiF *RD).

Dose	S/F	Invasion of cells	Cell proliferation of tumor cell lines	Migration of tumor cell lines	Apoptosis of tumor cell lines	Neoplasia of tumor cell lines	Quantity of muscle cell lines
6.0 Gy	S	0.26	-0.47	-0.17	-0.06	-1.09	1.00
	F	0.69	-0.31	0.74	0.06	0.22	2.00
6.5 Gy	S	0.69	1.29	-0.02	0.06	0.22	2.00
	F	2.02	-0.31	0.74	0.06	0.22	-2.00
7.0 Gy	S	1.20	1.51	1.68	-1.41	0.66	-1.00
	F	2.02	2.52	1.68	-1.51	-0.22	-2.00
7.5 Gy	S	2.79	1.73	0.74	-0.22	-1.09	0.00
	F	2.99	1.73	0.74	-0.67	-0.22	-2.00
8.0 Gy	S	1.25	2.07	0.93	-0.67	-1.09	-1.00
	F	2.79	1.73	0.74	-0.77	1.09	-2.00
8.5 Gy	S	-0.82	-0.35	0.74	0.62	-0.22	-1.00
	F	-0.69	-0.43	-0.74	0.21	0.22	-1.00

Bionetworks with a Z score of ≥ 1 and ≤ -1 were considered activated and inhibited networks, respectively. The tables included those bionetworks, which scored ≥ |1| in at least one of all tested conditions.

S: survived; F: fatal.

### Prognostic biomarkers to score the risk of radiation-induced fatality or RRiF

**Fig 7:**
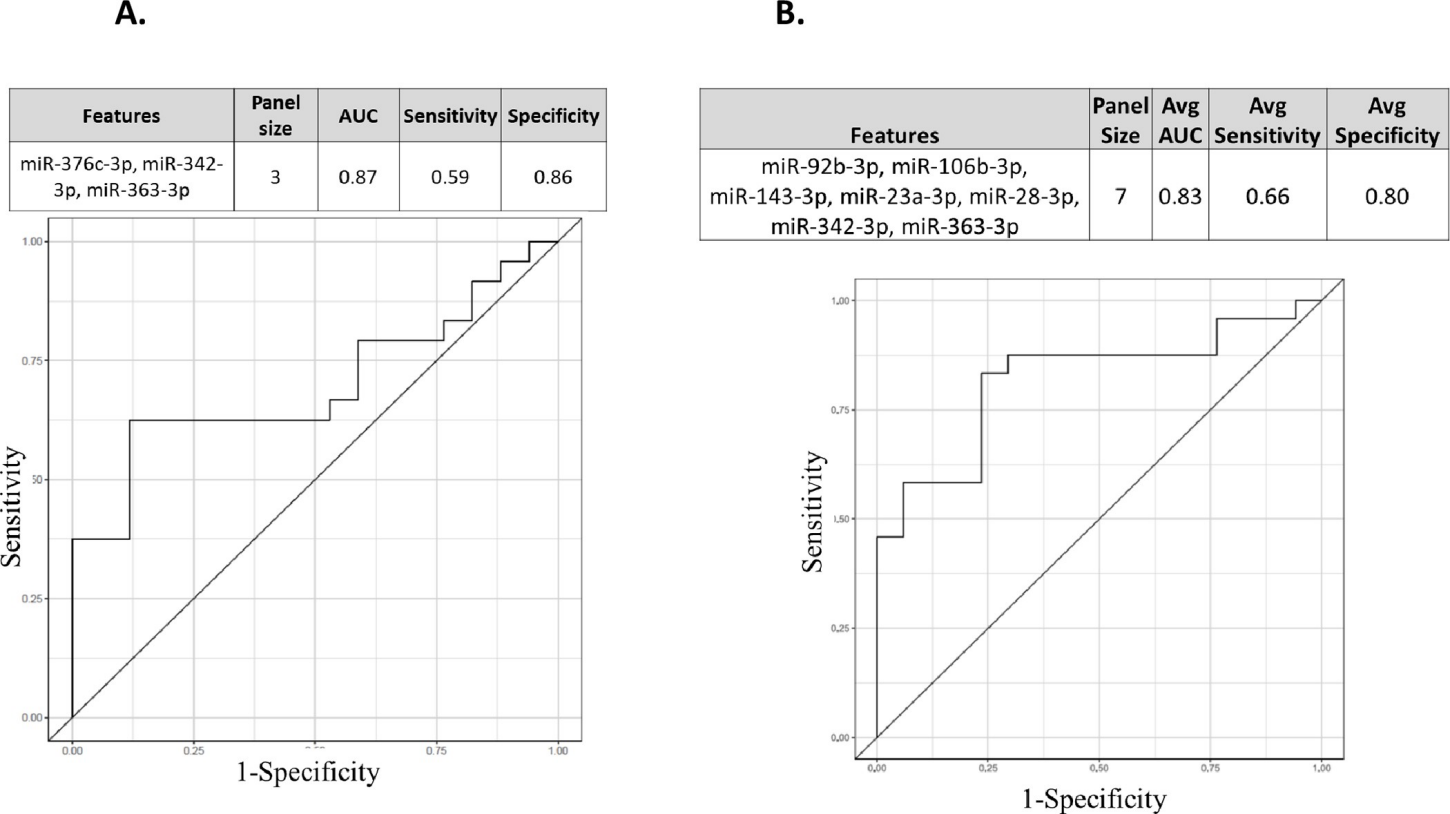
An miRNA panel to predict RRiF. **Two putative top performing markers were listed herein. (A)** This three-miRNA enriched panel was determined by k-fold routine of the 2BDP algorithm. AUC ROC plot is included herewith. **(B)** A seven-miRNA enriched panel was determined by RSBMR routine of the 2BDP algorithm. AUC ROC plot is included herewith.


The proprietary algorithm 2BDP curated miRNA panels that can predict RRiF. As described before, 2BDP used two methods to calculate the AUC of a given panel of miRNA biomarkers (Fig 3). Method 1, namely k-fold algorithm, identified 7 panels of miRNA biomarkers that could predict the RRiF with AUC > 0.85 (S4A Table). The top ranked candidate panel is presented in Fig 7A that conjoined three miRNAs (miR-376c-3p, miR-342-3p and miR-363-3p) through the following Eq 2 to achieve AUC = 0.87 with 95% CI ranging from 0.53 to 0.86.

$$logit(CtS)=0.58-0.41*X_{miR-376c-3p}-0.38*X_{miR-342-3p}-0.68*X_{miR-363-3p}$$ (Eq 2)

Method 2, namely RSBMR, failed to identify any panel that scored greater than 0.85 AUC. Herein, we listed 14 panels of miRNA biomarkers that could predict the RRiF with AUC >0.82 (S4B Table). The top ranked candidate panel is presented in Fig 7B that conjoined seven miRNAs namely miR-92b-3p, miR-106b-3p, miR-143-3p, miR-23a-3p, miR-28-3p, miR-342-3p, and miR-363-3p through the following Eq 3 to achieve AUC = 0.83 with 95% CI ranging from 0.65 to 0.94.


$$logit(CtS)=0.19+0.41*X_{miR-92b-3p}-0.47*X_{miR-106b-3p}+0.50*X_{let-143-3p}+0.84*X_{miR-23a-3p}-1.98*X_{miR-28-3p}-0.47*X_{miR-342-3p}+0.70*X_{miR-363-3p}$$ (Eq 3)

## Discussion

A significantly low success rate of human clinical studies has long been attributed to the ineffective translation of animal data to the clinical space [37]. This challenge is compounded in radiation studies due to the scarcity of human data with ‘true’ signal linked to radiation without any confounding factors, such as comorbidities like cancer. NHPs present the most viable platform to test radiation biomarkers since the gene heterogeneity between humans and NHPs is around 2%, although the scale of heterogeneity in small non-coding RNA regions is potentially higher [54, 55]. Hence, we took care in curating those miRNAs that registered sequential homology between humans and NHPs and would potentially display similar functionality in responding to radiation exposure. We identified a pool of 151 miRNAs and named it *cnvd*-miRNAs following an *in silico* routine that we have used earlier to identify miRNAs that were potential homologues between minipigs and humans [38]. In this previous study, male adult minipigs were exposed to LD_70/45_ doses and 92 miRNAs were found to be functionally and sequentially conserved between minipigs and humans. Of these 92 miRNAs, 27 were common with the present pool of 151 *cnvd*-miRNAs, potentially suggesting a phylogenetically conserved pool of miRNAs linked to radiation response.

In this respect, we have reported 7 miRNAs that were conserved among mice, humans and NHPs with radiation doses ranging from LD_30/60_, LD_50/60_, and LD_70/60_ [28]. A Venn analysis (S3 Table) found 5 of these seven miRNAs, namely miR-133a-3p, miR-215-5p, miR-150-5p, miR-30a-5p and miR-375-3p overlapped with 151 *cnvd*-miRNAs. The divergence between our current result and that of our earlier study [28] could be attributed to differences in the approaches to identify conserved miRNAs. For instance, our earlier study [28] established a cross-species homology by curating those miRNAs that target genes with overlapping sequences across mice, NHPs and humans. Furthermore, the range of radiation doses were mostly different between these two studies; namely, our earlier study [28] exposed NHPs to the following radiation doses: 5.8, 6.5, or 7.2 Gy, while the current study exposed NHPs to a wider range of radiation doses, namely 6.0, 6.5, 7.0. 7.5, 8.0 and 8.5 Gy. Regardless, the outcomes of these studies overlapped as described in S5 Table.

S5 Table lists the miRNAs distributed among the three pools, namely (i) 151 *cnvd*-miRNAs or the conserved miRNAs between humans and NHPs, which is the deliverable of the current study, (ii) 92 *cnvd*-miRNAs or the conserved miRNAs between humans and minipigs as reported here [38], and (iii) seven evolutionary conserved miRNAs among mice, NHPs and humans, which was published here [28]. S5 Table listed the potentially conserved miRNAs with high translational potential. Furthermore, we mapped our current data with a systematic review of more than 450 articles [30], which reported eight miRNAs that were significantly linked to radiation irrespective of the species model. The 151 *cnvd*-miRNA pool included 6 of these 8 miRNAs (miR-150, miR-29a, miR-30c, miR-200b, miR-30a and miR-150), emphasizing a cross-species applicability of these miRNAs in radiation biology.

We employed this pool of 151 *cnvd*-miRNAs to meet two objectives. The first objective was to determine the RD- and TSI-sensitive miRNA signatures and corresponding molecular bionetworks. Thereby, we elucidated the post-irradiation biomechanism dynamics with gender-specific resolution. The second objective was to determine the bionetworks leading to the survival of the subjects and pertinent dose-specific information was presented. Furthermore, we used our proprietary ML-algorithm named 2BDP [53] to deliver the putative prognostic markers linked to the risk of radiation-induced fatality or RRiF.

A multivariant analysis followed by a linear regression model delivered a set of miRNAs that consistently and significantly shifted with either increasing radiation dose or time. Of this set of miRNAs, miR-183-5p significantly distinguished the irradiated subjects from the non-TBI baseline, irrespective of the radiation doses; thereby, miR-183-5p was promoted as a potential radiation marker. In fact, multiple studies endorsed miR-183-5p as a potential signature of various carcinomas like prostate cancer [56], renal cancer [57] and gastric cancer [58], and additional reports found miR-183-5p’s radioresistant ability [59]. Our model found miR-183-5p was inhibited by high RDs, which could be linked to the role of down-regulated miR-183-5p in modulating potassium channels [60] and to escalated neuropathic pain disorders [61].

Subsequent multivariate analysis identified a group of miRNAs and their corresponding networks that could explain the dependent variable namely RD*TSI, and its gender bias, namely Sex*RD*TSI. Of this group of miRNAs, miR-424-5p, miR-30c-5p, and miR-30a-3p were functionally and sequentially conserved across minipigs, NHPs and humans and biologically both miRNAs are linked to the invasion and proliferation of carcinoma [62, 63].

Enumeration of the interconnecting nodes and edges of the miRNA networks linked to RD*TSI and Sex*RD*TSI revealed the hub molecules, which can contribute to multiple biofunctions in pleiotropic fashions [64, 65]. Indeed, one hub molecule, namely let-7a-5p was promoted as a potential radiation marker using a hematopoietic humanized mouse model [66]; furthermore, multiple studies reported the carcinogenic attributes of let-7a-5p [67]. In fact, all the hub miRNAs that were reported in the current study were found to be oncogenic in character highlighting the carcinogenic consequences of radiation [68].

Functional analysis of the miRNA profiles associated with RD*TSI revealed a dose-sensitive trend, particularly at 6 days post-irradiation. For instance, the bionetwork linked to apoptosis was activated and in conjunction with the cell viability network, was inhibited at low doses, namely 6.0–7 Gy [69]. Beyond 7 Gy, bionetworks linked to tumor cell invasion became activated, re-emphasizing the direct association between cancer and radiation [68]. A sex-biased trend dominated at the lower range of RDs, but both sexes responded similarly to the higher doses of radiation. This result might help in developing precision radiation therapy since a sex-based customization of radiation therapy seemed more warranted at the low range of radiation doses rather that its high range of doses.

The determination of prognostic markers linked to radiation-induced fatality and the comprehension of pertinent molecular attributes remain of high importance in radiation epidemiology studies. Since the survival tests depend on the animal model, its transition potentials are often questioned. Hence, we not only assayed an NHP model, which is one of the closest neighbors of humans in the phylogenetic tree, but also applied an *in silico* pipeline to compile those miRNAs that displayed sequential and potentially functional homology between humans and NHPs. Let-7a-5p re-emerged as a major hub molecule among the group linked to RRiF highlighting this marker’s diagnostic and therapeutic values in the context of radiation [66]. There were three additional miRNAs that were repeatedly featured as hub molecules in the networks linked to RD*TSI and RRiF; among them, miR-23a-3p [70] and miR-16-5p [71] are established oncogenic markers, and miR-7a-5p was identified as a potential marker of radiation exposure in a humanized mouse model [66].

Functional analysis found inhibited bionetworks linked to cell quantity, particularly muscle cell quantity, as the distinct features leading to radiation-induced fatality. These bionetworks were typically suppressed from 6.0 to 8.5 Gy suggesting a link between muscle wasting and radiation-induced fatality. Muscle wasting is a typical comorbidity of cachexia [72], a signal of the terminal phase of several diseases including cancer [73]. In fact, a recently published review article presented a panel of six miRNAs that were potentially linked to cachexia development [74] and one of them, namely miR-92a-3p, overlapped with our list that is linked to RRiF.

The current hypothesis states that a systematic integration of a group of biomarkers can potentially demonstrate higher efficacy than a single candidate biomarker [75]. Indeed, there is a clear trend in finding biomarker panels of different disease pathogenesis by taking the advantages from high throughput and high-resolution multi-omics readouts [75–77]. One of the early breakthroughs of this trend was Mamaprint, a 70-gene panel that was approved by the FDA as a prognostic marker for breast cancer relapse [78, 79]. Following this trend, we trained the 151 *cnvd*-miRNAs via the 2BDP algorithm to find a putative panel of biomarkers to predict radiation-induced fatality. In the past, we demonstrated the efficacy of 2BDP in grading different stages of Alzheimer’s disease by panels of gene markers [53]. Furthermore, we used this algorithm to determine a panel of gene markers to predict the onset of sepsis (patent pending). In the present study, the deliverables were to find panels of miRNAs to score the risk of radiation-induced fatality or RRiF. As described earlier, 2BDP operated on two parallel routines, which differed in process of determining and computing Training vs. Test sets, but eventually produced the best possible fitting model to explain a clinical event of interest, which was RRiF in the current study. One of the two fitting models of 2BDP, namely the k-fold algorithm, delivered a 3-miRNA panel that could score RRiF with an AUC ~ 0.87. Herein, all three featured miRNAs are linked to cancer; for instance, miR-376c-3p promotes apoptosis in cancer cells [80], miR-342-3p suppresses the invasion and proliferation of cancer cells [81] and miR-363-3p acts as an inhibitor of tumor growth [82]. K-fold methods delivered a few more panels that could be worth further consideration. For instance, there was a 5-miRNA panel with a slightly inferior AUC ~ 0.85, but better McFadden’s R^2^ ~ 0.19 than that of the above mentioned 3-miRNA panel. This 5-miRNA panel also featured the same three miRNAs, namely miR-376c-3p, miR-342-3p and miR-363-3p; additional candidates included miR-143-3p and miR-301a-3p that function as anti-carcinogenic [83] and pro-carcinogenic [84] factors, respectively. The second fitting model under 2BDP, namely RSBMR, delivered the prognostic panels for RRiF with inferior AUCs than that of the k-fold models. The best performing panel from RSBMR could predict RRiF with AUC ~ 0.82 and McFadden’s R^2^ ~ 0.28. It was a 7-miRNA panel that included the following: miR-92b-3p [85], miR-363-3p [85] and miR-143-3p [83] function as anti-carcinogenic factors; miR-106b-3p promotes metastasis [86]; miR-23a-3p is an oncogenic marker [70]; miR-28-3p is a marker of pulmonary diseases [87]; and miR-342-3p suppresses tumor cell proliferation [88].

As a limitation of this study, all these panels displayed comparatively low sensitivities, which could be attributed to a small sample size. Nevertheless, we must note that in the space of NHP research, six subjects per time point is not a trivial sample size. In addition, we took several measures to improve the outcome’s translational values. For instance, multi-level screening and *in silico* comparative routines were applied to curate those miRNAs that would display phylogenetically conserved responses to TBI. The blood samples collected from the pre-irradiated cohort were used as the baseline to minimize the individual sample bias. Furthermore, the 2BDP algorithm applied two unbiased routines to deliver the best fitting models which enabled the ability to score the risk of radiation-induced fatality. Because the present study used pre-irradiated samples as the baseline, the irradiated sample size outnumbered the unirradiated samples. Thus, we decided to refrain from using the 2BDP algorithm to find markers for radiation exposure, since being an ML-based algorithm, 2BDP could overfit the data. In the same token, we have certain confidence that 2BDP would produce viable results in predicting RRiF, since nearly half of the NHP cohort (16 of 36) survived across the radiation doses that were used in the current study.

In conclusion, we presented a pool of 151 serum miRNAs (*cnvd*-miRNA) that could have high translational potential in the context of radiation. The NHP samples collected from both genders and a wide range of radiation doses coupled with a high-resolution miRNA profiling assay and ML-empowered algorithm upheld the confidence level of the current results. Radiation dose- and time since irradiation-specific miRNA markers were identified; among them, miR-183-5p emerged as a leading candidate. Additional miRNA markers are identified that controls multiple biofunctions, such as suppressed cell proliferation, apoptosis, ion channel modulations, which leads to comorbidities like cancer and fatality. In addition, the 2BDP algorithm delivered candidate panels that can score the risk of radiation-induced fatality. In the clinical space, these panels could be used to triage the radiation exposed patients based on their risk of fatality, henceforth a customized precision care could be ensured. Validation studies of these miRNA panels using independent cohorts are warranted; nevertheless, the translational efficacies of these biomarker panels are expected to be high since these miRNAs affirm a cross-species homology.

Using these miRNA biomarkers developed from the candidate panels, an assay system could be developed to determine the dose of radiation exposure and the time since exposure with a high translational value. These biomarker panels have immense translational ability, and could be used in the clinic to not only triage radiation-exposed victims to determine their risk of death, but could also be used to assist in the development of precise radiation injury treatment strategies. Ultimately, these conservative strategies will optimize scarce resources during any large scale radiological/nuclear scenario and will provide optimal care to radiation exposed victims.

## Supporting information

S1 Fig Principal component analysis (PCA) of all the samples collected across radiation doses, time points and sex. (PDF)

S2 Fig **A.** Venn diagram showing the sub-clusters that were conserved and unique to the groups as defined in Fig 3A–3D, respectively. The underlined miRNAs were those which emerged most connected in Fig 3B or 3D. **B.** Venn diagram showing the sub-clusters that were conserved and unique to the groups as defined in Fig 4A–4D, respectively. The underlined miRNAs were those which emerged most connected in Fig 4B or 4D.
(ZIP)

S3 Fig Venn diagram to show the overlapping miRNAs across three conserved features, namely (i) Human and NHP conserved miRNAs, this list of 151 miRNAs was produced by the present work. (ii) Human and minipig conserved miRNAs, this list of 92 miRNAs is presented by our earlier work, (Chakraborty, N *et al*. Scientific Reports 13(1), 2023). (iii) Human-mouse-NHP conserved miRNAs, this list of 7 miRNAs was also published by us (Fendler, W. *et al*. Sci Transl Med, 2498 (9), 2017).
(PDF)

S1 Table A. Number of differentially expressed (DE) miRNAs identified by 15 dependent and independent variables under 4-way ANOVA model. **B.** List of 176 miRNAs’ log2 fold change values that emerged significantly expressed in at least one of the 15 analysis models using all dependent and independent variables. **C.** cnvd-miRNA: A display of the homologue sequences conserved between humans and NHPs.
(ZIP)

S2 Table List of miRNAs linked to major bionetworks. (PDF)

S3 Table A. Correlation matrix of 11 networks that were significantly regulated by RD*TSI. Pearson correlation was calculated across radiation doses and TSI. **B.** Correlation matrix of 11 networks that were significantly regulated by RD*TSI. Pearson correlation was calculated across radiation doses at 6-day post-TBI. **C.** Male vs. female correlation matrix of 7 networks that were significantly regulated by sex*RD*TSI. Pearson correlation was calculated across radiation doses and TSI.
(ZIP)

S4 Table A. K-fold results: List of potential panels that can predict RRiF with AUC >0.85. **B.** RSBMR results: List of potential panels that can predict RRiF with AUC >0.82.
(ZIP)

S5 Table The miRNA list linked to the Venn diagram S3 Fig. (PDF)
