# Supplementary figures and images for: Cross-species conserved miRNA as biomarker of radiation injury over a wide dose range using nonhuman primate model
Source: PLoS One. 2024 Nov 21;19(11):e0311379. doi: 10.1371/journal.pone.0311379 (PMC11581275; doi:10.1371/journal.pone.0311379)

S1 Fig.

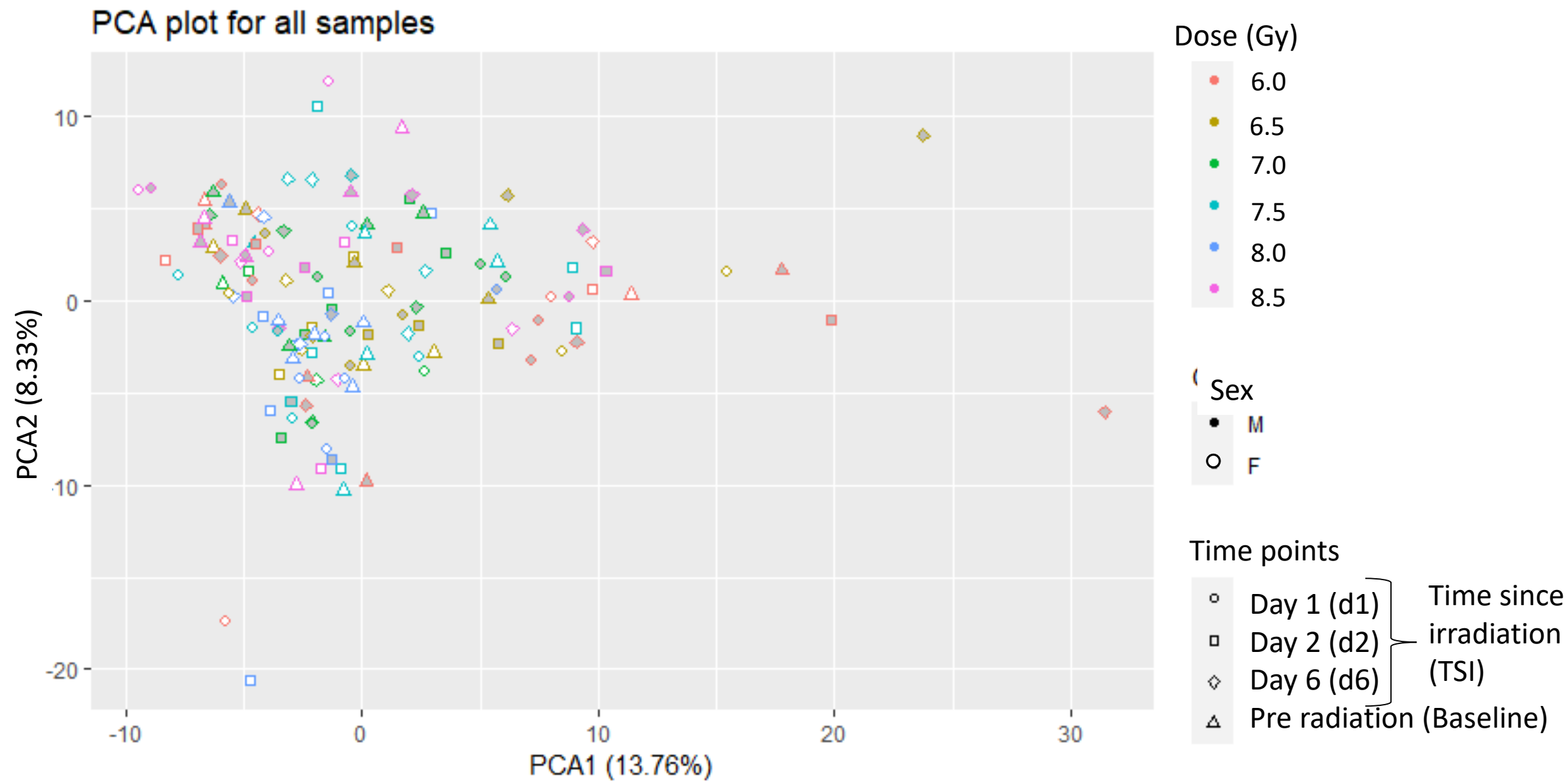

Supplement: S1 Fig — (PDF) [file pone.0311379.s001.pdf]

S2A Fig.

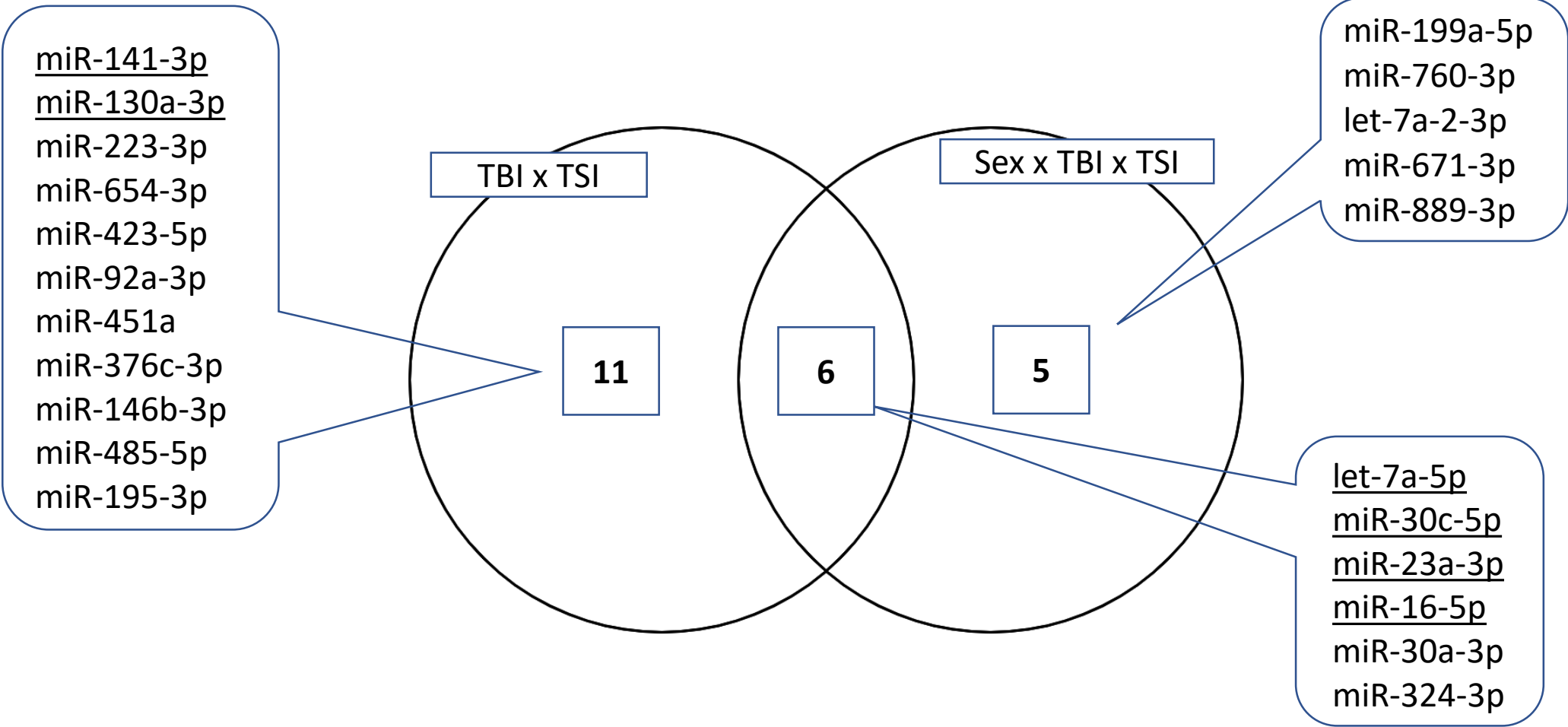

Supplement: S2 Fig — A. Venn diagram showing the sub-clusters that were conserved and unique to the groups as defined in Fig 3A–3D, respectively. The underlined miRNAs were those which emerged most connected in Fig 3B or 3D. B. Venn diagram showing the sub-clusters that were conserved and unique to the groups as defined in Fig 4A–4D, respectively. The underlined miRNAs were those which emerged most connected in Fig 4B or 4D. (ZIP) [file pone.0311379.s002.zip › S2A_Fig.pdf]

S2B Fig.

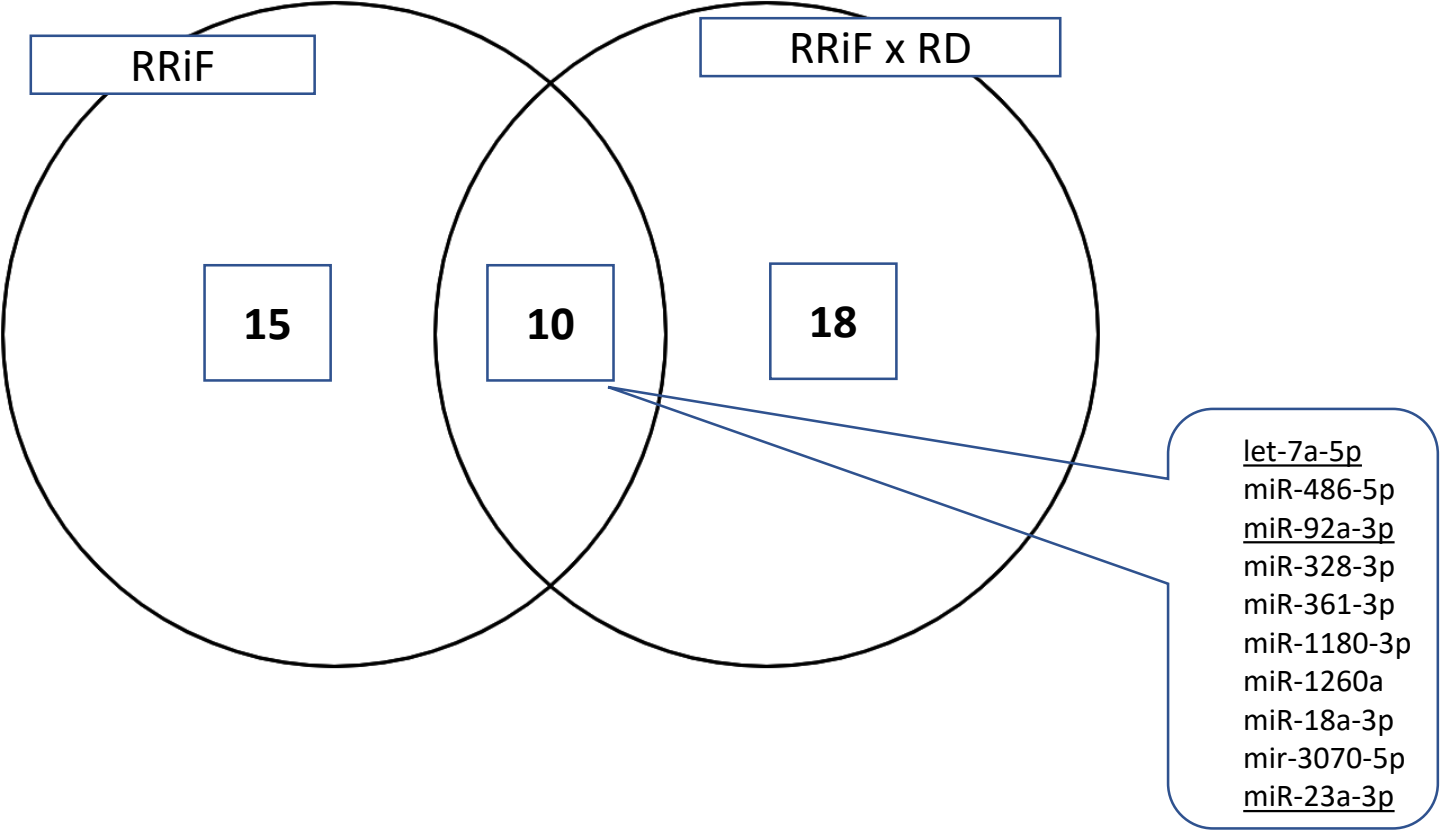

Supplement: S2 Fig — A. Venn diagram showing the sub-clusters that were conserved and unique to the groups as defined in Fig 3A–3D, respectively. The underlined miRNAs were those which emerged most connected in Fig 3B or 3D. B. Venn diagram showing the sub-clusters that were conserved and unique to the groups as defined in Fig 4A–4D, respectively. The underlined miRNAs were those which emerged most connected in Fig 4B or 4D. (ZIP) [file pone.0311379.s002.zip › S2B_Fig.pdf]

S3 Fig.

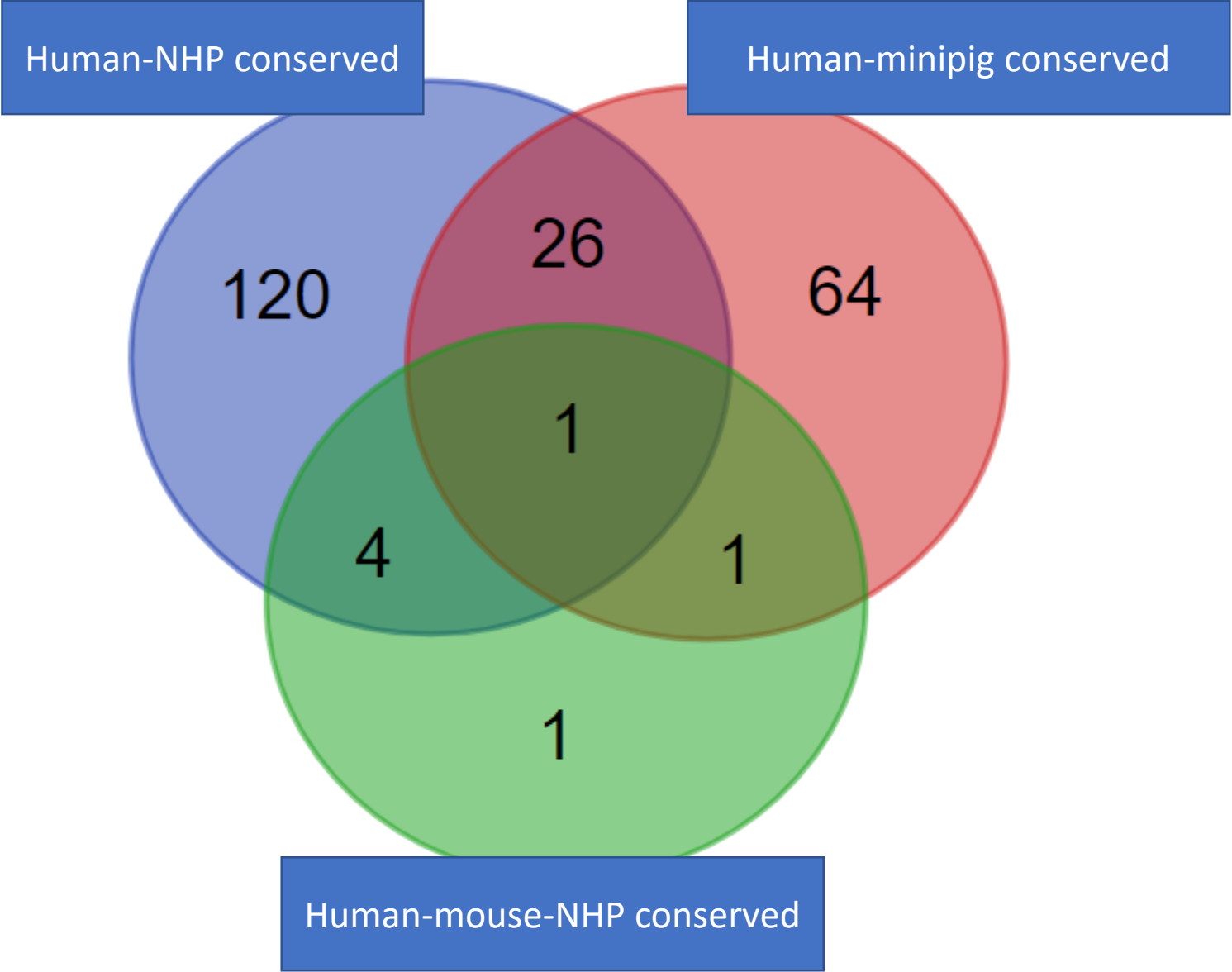

Supplement: S3 Fig — (ii) Human and minipig conserved miRNAs, this list of 92 miRNAs is presented by our earlier work, (Chakraborty, N et al. Scientific Reports 13(1), 2023). (iii) Human-mouse-NHP conserved miRNAs, this list of 7 miRNAs was also published by us (Fendler, W. et al. Sci Transl Med, 2498 (9), 2017). (PDF) [file pone.0311379.s003.pdf]
