# Supplementary material for: Cross-species conserved miRNA as biomarker of radiation injury over a wide dose range using nonhuman primate model
Source: PLoS One. 2024 Nov 21;19(11):e0311379. doi: 10.1371/journal.pone.0311379 (PMC11581275; doi:10.1371/journal.pone.0311379)
Supplement: S1 Table — B. List of 176 miRNAs’ log2 fold change values that emerged significantly expressed in at least one of the 15 analysis models using all dependent and independent variables. C. cnvd-miRNA: A display of the homologue sequences conserved between humans and NHPs. (ZIP) [file pone.0311379.s004.zip › S1A_Table.pdf]

S1A Table. Number of differentially expressed (DE) miRNAs identified by 15 dependent and independent variables under 4-way ANOVA model

Variables	Number of differentually expressed miRNAs
Dosimentry or Total body irradiation (TBI)	54
Time since irradiation (TSR)	33
Sex	18
Risk to radiation induced fatality (RRiF)	34
TBI*TSR	25
TBI*Sex	17
TSR*Sex	9
TBI*RRiF	31
TSR*RRiF	5
Sex*RRiF	15
TSR*TBI*Sex	12
TSR*TBI*RRiF	12
TSR*Sex*RRiF	12
TBI*Sex*RRiF	12
TSR*TBI*Sex*RRiF	23
