# Supplementary material for: Cross-species conserved miRNA as biomarker of radiation injury over a wide dose range using nonhuman primate model
Source: PLoS One. 2024 Nov 21;19(11):e0311379. doi: 10.1371/journal.pone.0311379 (PMC11581275; doi:10.1371/journal.pone.0311379)
Supplement: S1 Table — B. List of 176 miRNAs’ log2 fold change values that emerged significantly expressed in at least one of the 15 analysis models using all dependent and independent variables. C. cnvd-miRNA: A display of the homologue sequences conserved between humans and NHPs. (ZIP) [file pone.0311379.s004.zip › S1B_Table.pdf]

S1B Table. List of 176 miRNAs' log2 fold change values that emerged significantly expressed in at least one of the 15 analysis models using all dependent and independent variables

miRNA	Day1 6 Gy	Day1 6 Gy	Day1 6.5 (	Day1 6.5 (	Day1 7Gy	Day1 7Gy	Day1 7.5G	Day1 7.5G	Day1 8Gy	Day1 8Gy	Day1 8.5G	Day1 8.5G	Day2 6Gy	Day2 6Gy	Day2 6.5G	Day2 6.5G	Day2 7Gy	Day2 7Gy	Day2 7.5G	Day2 7.5G	Day2 8Gy	Day2 8Gy	Day2 8.5G	Day2 8.5G	Day6 6Gy	Day6 6Gy	Day6 6.5G	Day6 6.5G	Day6 7Gy	Day6 7Gy	Day6 7.5G	Day6 7.5G	Day6 8Gy	Day6 8Gy	Day6 8.5G	Day6 8.5Gy M
mml-let-7a-3p	-0.06	1.19	0.24	0.17	0.70	0.00	1.35	0.57	0.00	0.18	-0.08	-0.15	0.10	1.17	0.96	0.89	1.05	-1.41	-0.45	0.82	-0.58	0.27	0.52	-2.07	0.23	0.47	0.15	-0.06	0.34	0.04	-2.11	0.72	0.41	-0.47	-0.01	-0.28
mml-let-7a-5p	-0.88	-1.09	-0.69	0.57	-0.70	-0.08	0.03	-0.78	0.60	-0.45	0.07	0.27	-0.56	-0.45	0.04	0.29	-0.42	0.40	-0.13	-0.66	-0.12	-0.12	-0.21	-0.04	0.09	-0.90	-0.61	-0.09	-0.54	-0.30	0.01	-0.93	0.02	-0.87	-0.74	-0.34
mml-let-7b-3p	0.50	2.69	1.08	0.20	0.33	3.25	2.03	0.46	-1.62	0.46	-0.44	0.78	0.13	2.39	1.40	1.05	1.72	1.24	2.13	0.37	1.19	0.35	-0.07	1.33	0.74	0.96	1.00	1.18	1.60	2.06	-0.11	1.21	-0.17	1.06	-0.27	1.46
mml-let-7c-5p	-1.56	0.72	-0.22	-0.08	0.27	0.00	-0.55	-0.60	3.98	0.53	-0.72	-1.05	-0.12	0.62	1.07	1.09	0.92	-0.09	0.48	-1.06	0.89	0.29	-1.12	0.33	-0.34	0.78	1.72	-0.64	0.38	-0.37	-1.50	-0.80	0.19	-0.74	1.80	-0.15
mml-let-7d	-0.45	-0.68	-0.20	0.95	-0.67	-1.78	0.34	-0.81	0.10	-0.12	-1.86	0.22	-0.52	-0.32	-0.53	-0.17	-0.75	-0.96	-0.12	-0.46	-0.06	-0.02	0.10	0.01	0.65	-0.48	-0.36	0.11	-1.21	-1.19	-0.34	-0.38	-0.26	-0.10	-0.77	0.30
mml-let-7e-5p	-1.78	0.37	-1.42	-0.75	-0.79	0.11	-0.43	-1.22	1.13	-1.10	-0.57	-1.52	-1.60	1.34	-1.42	-0.47	-0.11	0.11	0.41	-0.06	-0.10	-0.44	-0.26	-1.07	-1.04	0.77	0.59	0.46	0.03	0.34	0.75	-0.91	-0.24	-1.69	0.18	-0.83
mml-let-7f-3p	1.05	1.97	1.33	-0.20	0.44	0.00	0.00	1.37	0.00	0.41	-0.76	0.71	0.12	3.35	1.37	-0.42	0.22	0.00	0.00	0.87	-0.99	0.30	0.42	1.67	0.62	1.88	1.07	-0.68	0.84	1.21	0.00	1.29	0.58	0.28	-0.30	0.81
mml-let-7f-5p	-0.80	-0.84	-0.48	-1.40	-1.01	-1.56	-0.90	-0.19	-1.55	-1.00	-2.04	0.10	-1.05	0.29	-0.17	0.02	-0.54	0.62	-0.99	-1.20	-0.12	-0.14	-0.51	0.15	-0.74	-0.87	-1.61	-0.06	-0.53	-0.69	0.28	-0.21	-0.81	-1.02	-0.79	-0.68
mml-let-7g-3p	1.64	0.00	0.79	2.91	0.48	0.00	0.00	1.06	0.00	0.42	-0.63	-0.13	1.87	0.00	0.15	-0.11	0.63	0.00	0.00	0.40	0.42	0.37	-0.44	1.39	1.94	0.00	0.08	0.88	0.98	1.21	0.00	1.41	1.00	0.63	0.40	-0.54
mml-let-7g-5p	-1.14	0.36	-0.33	-0.46	-0.36	-0.38	-0.06	-0.32	-0.36	-0.44	0.07	-0.40	-0.88	0.05	-0.03	0.20	-0.40	-0.14	-0.50	-0.75	0.23	-0.45	-0.16	0.10	-0.47	-0.50	-1.23	0.04	-0.21	-0.19	-0.66	-0.75	-0.82	-0.96	-0.23	-0.86
mml-let-7i-5p	-0.42	-0.52	-0.17	0.04	-0.17	-0.15	-0.08	-0.06	0.82	-0.41	0.30	-0.02	-0.47	-0.28	-0.10	0.36	-0.13	-0.41	0.30	-0.59	0.07	0.17	0.20	0.37	0.05	-0.65	-0.15	0.40	-0.10	-0.71	-0.41	-0.39	0.04	-0.37	-0.85	-0.80
mml-miR-101-1-5p	0.08	1.74	0.87	0.07	0.33	0.00	0.00	-0.62	0.00	-0.26	0.67	0.57	0.26	1.34	-0.14	0.25	0.00	2.08	0.00	-0.10	0.00	-0.26	-0.10	-0.43	1.20	0.00	-0.21	0.30	0.16	0.00	0.00	-0.05	2.26	-0.55	0.65	-0.11
mml-miR-101-3p	-0.29	0.59	0.28	-1.03	-0.52	-0.39	-0.16	0.73	2.12	0.01	0.63	-0.14	-0.86	-0.60	-0.44	0.12	-0.24	-0.52	0.78	-0.21	0.82	0.12	-0.29	0.47	-0.44	-0.79	-1.50	0.05	0.17	-0.57	-0.54	-0.14	0.96	0.31	-1.07	0.12
mml-miR-106b-3p	-0.89	-0.63	-0.47	1.40	-0.49	1.25	0.20	-1.23	-0.42	0.53	0.72	-0.15	-0.77	-0.62	-0.08	-0.09	0.11	0.52	0.02	-0.46	0.46	0.51	1.65	-0.18	0.18	-0.54	0.03	0.00	-0.90	1.21	0.08	-0.93	1.14	-0.21	-0.10	0.76
mml-miR-106b-5p	-1.05	1.49	0.42	0.20	0.35	-1.91	0.18	0.14	0.44	-0.18	-0.17	-0.81	-0.78	0.40	-0.09	0.12	0.06	-0.59	0.20	-0.18	0.74	-0.05	-0.14	0.01	0.15	0.12	-1.60	0.64	-0.09	-0.26	-1.59	-1.47	-1.13	-0.09	-0.87	-0.42
mml-miR-10a-3p	-0.38	0.33	0.58	0.85	-0.11	3.57	0.03	0.43	0.00	1.55	0.07	0.82	0.57	1.30	0.36	1.41	0.70	3.34	0.55	0.16	0.70	0.81	0.72	1.66	0.28	0.71	0.32	1.06	0.40	4.02	-0.11	0.10	-0.23	1.03	0.06	1.10
mml-miR-10a-5p	-0.11	0.26	-0.29	-0.19	0.36	0.92	0.17	-0.17	-1.84	0.27	-0.82	-0.54	-0.54	0.99	0.94	-0.17	0.12	1.17	-0.26	0.55	1.15	0.39	-0.14	0.20	0.35	-0.80	0.54	0.08	0.54	1.83	2.26	1.31	0.99	0.46	-0.56	-0.26
mml-miR-1180-3p	-1.50	2.11	-1.56	1.10	0.84	1.69	0.29	-1.32	0.00	0.65	-1.91	-0.52	-0.96	2.64	-0.36	-1.17	-0.09	-0.12	-0.78	-0.89	-0.12	-0.03	-0.25	-0.87	0.20	0.55	0.15	-0.69	-0.39	0.07	0.38	-0.81	0.95	0.06	-0.38	-0.36
mml-miR-1185-3p	0.81	-0.26	-0.41	0.29	0.35	0.00	-0.26	-0.79	0.00	-0.13	0.47	1.64	1.91	1.24	0.45	-0.12	0.66	-0.09	-0.91	-0.28	-3.28	0.19	-0.72	0.91	0.99	0.58	-0.49	-0.31	0.68	-1.96	0.00	0.65	-0.38	0.59	0.68	0.77
mml-miR-122a-5p	0.74	-0.03	-1.24	0.38	-0.98	0.00	0.03	-0.81	0.00	2.31	-0.86	-0.11	-0.76	1.90	-0.59	-0.42	0.00	0.00	0.00	-0.43	0.00	0.99	0.00	-2.00	-0.44	0.92	-1.36	2.00	-2.13	0.00	2.89	-0.45	0.00	1.15	-0.39	0.00
mml-miR-1235	0.49	1.83	0.90	0.15	0.24	0.00	0.03	0.16	0.00	0.95	0.41	0.31	0.00	0.00	0.15	0.07	0.40	0.00	0.00	0.13	2.42	0.07	1.01	0.09	0.03	0.00	0.00	0.05	0.28	0.00	0.00	0.41	0.00	0.16	0.56	2.31
mml-miR-1247-5p	1.20	6.43	0.50	0.12	0.77	0.00	0.00	-1.93	0.00	0.12	1.47	-0.77	0.22	1.40	0.48	0.25	0.23	0.00	0.00	-0.66	0.42	-0.03	0.58	-1.66	-0.41	2.12	1.03	-0.04	0.50	0.00	0.00	-0.63	0.00	-0.60	1.33	-1.76
mml-miR-125b-1-3p	-0.17	0.74	0.58	-0.54	-0.15	0.00	0.62	1.32	0.00	0.02	-0.96	-0.12	0.83	0.64	0.91	0.39	1.19	0.00	0.55	1.35	0.00	-0.13	-0.02	1.00	0.61	1.58	1.67	-0.37	0.94	3.21	1.48	1.77	1.41	-0.81	0.67	-0.18
mml-miR-125b-2-3p	0.07	0.62	0.78	0.28	0.33	1.11	-0.09	-0.03	0.00	-0.15	-0.18	-0.47	0.61	1.53	0.38	0.97	0.45	-0.53	0.77	0.21	1.97	0.10	0.27	0.54	-0.25	0.52	1.61	0.74	0.52	-0.25	0.39	0.40	2.94	-0.31	0.91	1.22
mml-miR-125b-5p	-0.09	0.55	-0.33	-0.26	0.35	0.00	1.20	-2.10	4.36	0.47	0.62	0.10	1.49	0.93	0.65	-0.79	1.21	1.17	1.13	-0.61	1.76	-0.65	-0.30	1.25	0.98	-0.08	1.27	-0.25	-0.32	2.21	2.41	-0.62	-0.91	-0.91	0.31	0.56
mml-miR-126	-0.88	-0.48	-0.02	-0.02	-0.88	0.51	0.45	-0.13	-0.76	0.41	-0.68	-0.20	-0.91	0.55	-0.66	0.52	0.03	0.28	-0.75	-0.74	0.16	-0.47	-0.39	-0.08	-0.87	0.45	-0.73	0.37	-0.14	0.58	-0.63	-0.05	-0.14	0.11	-0.79	-0.07
mml-miR-1260b	-2.01	-2.47	-1.85	1.78	-0.64	0.40	0.25	-2.08	2.68	0.50	-1.78	1.24	-1.46	-2.04	-1.48	-1.03	-0.62	2.29	-0.04	-0.17	-0.82	-0.14	-0.43	0.04	-0.77	-3.34	0.10	-0.41	-1.61	-0.37	1.55	-0.34	0.91	-0.43	-0.81	1.18
mml-miR-1271-3p	0.12	4.75	1.45	1.00	0.60	0.00	3.35	2.08	0.00	1.31	1.14	0.08	0.57	1.23	0.00	0.58	-0.24	1.76	0.00	0.94	-0.58	0.30	0.53	0.52	0.50	0.71	1.12	0.48	-0.74	1.63	0.00	1.69	1.00	0.14	0.66	-0.09
mml-miR-128a-3p	-0.26	-1.01	-0.03	-0.83	0.04	-0.13	0.67	0.88	0.35	0.91	2.03	0.52	-0.79	-1.06	0.20	-1.18	0.21	0.60	0.27	0.99	0.13	0.38	0.84	0.20	0.07	-1.21	1.09	-0.41	0.39	-0.27	0.42	0.57	-1.14	0.21	-0.12	0.07
mml-miR-1296-5p	1.04	0.00	0.00	0.17	-0.12	1.98	0.00	2.36	0.00	0.65	0.29	0.42	0.54	0.00	0.00	0.15	-0.19	0.76	0.00	1.84	1.42	-0.06	0.02	0.22	0.42	0.00	0.34	-0.16	0.59	0.00	0.00	0.82	0.00	0.65	0.53	0.30
mml-miR-1306-5p	-1.22	3.35	-0.17	3.29	-0.64	0.00	-0.55	-1.64	0.00	0.76	0.12	1.42	-0.87	3.79	0.77	1.60	-0.24	0.00	-0.04	0.10	-1.16	2.13	0.32	-0.25	-0.42	1.21	-0.14	0.78	-0.53	1.21	0.31	0.04	0.83	1.32	0.00	1.82
mml-miR-130a-3p	0.15	-0.91	0.45	-0.61	-0.60	-1.96	-0.14	0.63	-0.56	-0.04	-0.98	0.67	-0.23	0.11	-0.48	1.04	-0.11	-0.03	0.22	0.23	-0.69	0.24	-0.01	0.68	0.56	0.13	-0.23	0.51	-0.01	0.00	-1.32	0.47	-0.24	0.32	-0.85	0.20
mml-miR-130b-5p	0.30	0.36	0.08	1.91	-0.44	0.00	0.00	-0.71	4.06	1.49	-1.38	1.06	0.56	2.72	0.10	-0.10	-0.22	0.00	0.00	0.05	0.10	0.42	-1.04	-0.54	1.08	-0.08	0.52	0.25	-0.67	0.00	0.00	0.44	-0.33	0.47	-0.55	1.26
mml-miR-133a	1.44	0.00	1.46	0.35	0.18	3.57	5.79	1.93	0.00	4.76	0.64	1.41	0.56	0.00	-0.10	0.12	-0.30	-0.24	4.13	0.85	3.81	2.54	0.86	-1.57	0.70	0.00	0.03	-1.25	-0.74	1.21	1.48	0.09	3.32	1.35	0.15	-1.00
mml-miR-133c-3p	0.34	0.00	0.96	-0.10	0.18	3.57	0.00	4.16	0.00	3.45	0.85	0.70	-0.04	0.00	0.62	-0.35	-0.26	0.76	0.00	2.33	0.00	1.34	0.03	-1.77	-0.25	0.00	0.39	-0.48	-1.05	0.00	0.00	0.00	0.00	1.18	-0.11	-0.72
mml-miR-136	0.69	0.38	-0.42	0.19	-0.48	0.00	0.37	1.82	0.00	-0.20	0.35	0.59	0.42	0.50	-0.95	0.34	-0.94	-0.05	-1.36	0.50	-1.16	0.36	0.14	-1.11	0.15	1.01	-0.02	0.56	-0.48	-1.60	-0.69	1.71	1.32	1.50	0.26	0.56
mml-miR-140-3p	-0.24	-0.57	-0.24	-1.02	-0.40	-1.45	-1.30	0.14	-1.62	-0.39	-1.36	-0.83	-0.53	-1.40	-0.88	-0.18	-0.25	-1.02	0.33	-0.57	0.01	-0.20	-0.32	-0.10	-0.04	-2.37	-0.49	-0.56	0.01	-1.04	-2.13	-0.63	-1.08	-0.35	-0.17	-0.59
mml-miR-141-3p	1.25	7.59	2.25	0.66	1.11	3.33	3.11	2.11	0.00	0.54	1.51	-0.53	-0.34	4.83	1.08	-0.63	-0.07	1.70	-2.98	0.46	2.29	-1.21	1.01	-0.69	-0.02	2.38	0.37	-0.88	0.50	1.51	0.27	1.83	0.83	0.52	1.21	-0.43
mml-miR-142-3p	-1.39	-0.11	0.27	-0.54	-0.91	-1.24	-0.61	-0.45	0.00	-0.40	0.04	0.18	-0.06	-0.13	-1.52	-0.40	-1.05	1.04	-0.37	-0.53	-0.08	-0.86	0.27	0.33	-1.56	0.38	0.02	-0.18	-1.79	-1.01	-3.03	-0.93	-0.54	-1.13	-0.42	0.55
mml-miR-143-3p	-0.61	-0.57	-0.74	-0.63	0.07	-0.27	1.00	-0.75	1.66	0.78	-1.04	-0.98	-0.94	1.69	-0.21	0.58	-0.08	-0.05	0.82	-1.34	2.06	0.82	-0.34	-0.41	0.11	0.37	-0.32	0.06	0.63	2.18	2.76	0.69	4.11	0.75	0.64	-0.08
mml-miR-146b-3p	-0.65	-2.11	0.98	0.04	-0.30	0.00	-0.97	0.52	4.14	0.15	0.07	0.70	0.29	-4.31	2.04	-1.65	-0.81	-0.24	0.55	-0.22	-0.16	-0.27	0.66	-0.31	-1.07	-1.85	0.00	-1.37	-0.46	0.00	0.89	0.94	0.00	-0.33	0.00	1.77
mml-miR-150-5p	-1.28	-1.16	-0.61	1.04	-1.21	-0.25	-1.13	-1.38	4.02	-0.52	0.08	-1.92	-1.65	-1.66	-1.12	-0.61	-0.64	-1.69	-2.17	-1.81	-0.69	-2.47	-0.95	-2.54	-1.32	-2.44	0.07	-0.87	-2.72	-1.58	-3.75	-1.99	-1.24	-2.46	-0.75	-0.77
mml-miR-152-3p	-0.42	1.00	-0.30	1.39	0.41	1.98	0.03	-0.53	0.00	0.65	0.22	0.37	-0.04	1.22	0.40	0.32	1.05	0.17	1.77	-0.21	1.06	0.22	0.08	-0.10	-0.58	1.04	0.26	0.80	0.38	2.80	-1.69	-0.70	-0.17	-0.38	0.23	1.03
mml-miR-15b-5p	-0.43	-0.42	-0.80	0.35	-0.20	0.00	0.80	0.15	-1.46	0.04	-0.74	-1.09	0.41	-0.05	-0.95	-0.57	-0.71	-0.15	-0.19	-0.29	1.01	-0.21	0.95	-1.03	1.03	-0.99	0.34	-0.51	-0.66	-0.11	-0.26	-0.60	0.25	-0.64	-0.17	-0.80
mml-miR-16-2-3p	-0.34	1.82	-0.25	-1.02	0.52	-0.10	0.48	-0.32	-0.86	-0.77	-0.62	-1.64	-0.87	0.97	0.25	-0.62	-0.30	-0.84	0.97	-0.28	1.35	-0.37	0.11	-0.56	0.03	-0.20	-2.60	-0.03	0.74	0.49	1.14	-0.76	0.52	-0.43	0.01	-0.57
mml-miR-181a-5p	-0.14	-0.36	0.07	0.54	-0.24	-0.16	0.24	-0.19	0.71	-0.02	0.36	-0.08	-0.21	0.15	-0.29	0.50	-0.10	-0.45	-0.41	-0.72	-0.19	-0.33	0.15	0.23	0.37	-0.15	0.57	0.09	-0.35	-0.40	-1.07	-0.24	0.06	-0.23	0.00	0.01
mml-miR-181c-5p	-0.40	0.45	-0.36	0.73	-0.37	-1.99	0.48	0.38	-0.84	0.07	-0.22	-0.21	0.18	1.11	-0.82	0.55	0.19	-0.27	-0.62	0.10	-0.68	-0.39	-0.08	0.16	0.28	0.96	-0.52	0.41	-0.26	-1.02	-0.15	0.17	0.13	0.06	-0.80	0.42
mml-miR-182	-1.56	-0.86	0.15	2.95	0.18	1.57	0.62	-0.70	4.69	1.51	-0.11	-0.92	-1.39	-1.71	-0.21	0.37	-0.86	-2.83	-0.26	-0.01	1.29	0.71	-0.07	-0.35	-1.03	-0.84	1.04	-0.21	-2.53	-1.37	0.99	-0.37	1.90	0.26	2.38	0.50
mml-miR-183-5p	-0.31	0.00	0.74	0.81	0.71	0.00	0.00	-0.36	0.00	1.08	0.00	0.00	-0.91	-0.72	0.56	-0.39	1.55	0.00	0.00	0.00	0.42	1.56	0.75	0.13	0.26	-2.47	0.85	0.46	-0.08	0.00	0.00	0.45	2.00	0.94	0.00	0.35
mml-miR-185-3p	3.06	0.74	0.89	1.23	0.43	0.00	0.00	0.21	0.00	0.40	0.69	1.83	1.33	-0.06	1.13	-0.11	0.48	0.00	0.00	0.00	-0.38	0.38	0.20	1.33	2.70	0.00	0.00	1.00	-0.42	1.80	0.00	0.89	1.19	1.30	0.17	3.09
mml-miR-185-5p	-1.48	0.00	-0.49	1.65	0.29	0.00	0.00	1.21	0.00	1.30	0.09	3.89	-0.95	0.00	0.95	1.61	0.00	0.00	0.00	1.29	0.00	1.11	0.00	1.75	-0.54	0.00	-1.28	0.61	0.09	0.00	0.00	0.80	0.00	0.12	1.24	0.00
mml-miR-186-5p	-0.49	-0.22	-0.33	0.20	-0.52	-0.67	-0.18	-0.05	0.52	-0.38	-0.11	-0.51	-0.46	0.02	-0.24	0.03	-0.21	0.13	-0.72	-0.68	-0.48	-0.35	-0.01	0.01	0.16	-0.64	-0.38	-0.06	-0.31	-0.17	0.36	0.16	-0.27	-0.47	0.14	-0.12
mml-miR-187-3p	-0.40	-0.06	-1.19	-0.24	-1.11	0.00	-0.11	0.26	0.00	-0.73	-0.37	0.51	0.85	0.71	-2.02	-0.90	-0.49	0.08	-0.91	0.23	-1.96	0.22	-0.23	-0.53	-0.57	0.52	-1.24	-0.14	0.18	-1.73	1.13	0.40	-2.55	1.05	-1.18	0.06
mml-miR-18a-3p	-2.57	2.96	-1.20	2.23	-0.77	0.00	-2.78	-1.78	0.00	1.12	0.93	-0.23	-2.31	3.78	-0.06	0.68	-0.43	-0.83	-1.26	-1.15	2.18	0.76	-0.42	-1.17	-1.32	2.31	0.49	0.37	-2.11	0.00	-2.92	0.55	3.54	-0.73	-0.53	0.97
mml-miR-192-5p	-0.05	1.25	-0.34	-1.72	-1.77	1.49	-1.17	-1.15	0.04	-0.60	0.34	-0.83	-1.73	-0.25	0.62	-0.17	-1.73	-0.20	-2.08	-1.35	0.78	-0.82	0.11	-0.20	-0.72	0.22	-0.15	-0.17	-0.96	0.46	-1.76	-1.37	-0.64	-1.09	0.01	0.56
mml-miR-193a-3p	-0.23	2.69	3.33	0.07	0.71	0.00	0.88	1.85	0.00	2.79	1.59	0.03	-0.26	0.90	1.42	0.22	1.60	0.02	1.81	2.23	0.00	2.53	1.40	1.61	0.08	0.71	0.27	0.60	1.09	0.89	0.00	0.84	3.00	2.00	0.89	1.39
mml-miR-193b-3p	0.27	-0.64	1.43	-0.24	0.16	0.00	1.62	-0.54	0.00	0.30	-0.23	0.51	-0.12	-0.73	2.42	0.69	0.80	0.76	0.00	-0.60	-1.16	-0.21	0.33	0.32	-0.51	-0.74	1.25	-0.26	-0.22	-0.79	0.00	0.19	0.00	-1.23	-0.18	0.73
mml-miR-195-5p	0.01	4.22	0.45	0.73	1.03	0.00	3.20	-0.42	0.00	0.92	0.67	-0.19	-0.05	1.23	0.00	1.56	1.98	1.76	0.00	0.06	3.30	0.77	1.74	0.98	0.40	1.21	0.08	1.74	1.68	0.00	0.00	0.90	0.00	0.64	0.46	0.16
mml-miR-197-3p	-1.05	-0.35	-0.76	0.92	-0.12	0.92	0.55	-1.48	0.00	0.41	0.37	-0.68	-0.36	-0.25	0.27	-0.05	-0.19	1.11	-0.87	-0.36	0.36	-0.11	-0.51	-0.92	-0.35	0.43	-0.16	-0.36	-0.93	-1.43	1.65	-0.25	0.18	0.30	0.25	0.05
mml-miR-199a	0.21	-1.73	1.44	-0.14	0.24	0.40	0.62	-0.80	0.00	0.43	-0.83	-0.67	0.32	-1.47	0.32	-0.94	0.50	-2.41	2.13	0.03	0.42	0.21	0.20	-1.46	0.76	-1.83	0.28	0.23	0.70	-0.37	-1.11	-0.53	0.88	-0.04	-0.76	-0.06
mml-miR-199a-3p	-0.83	-0.52	-0.31	-0.39	-0.49	-0.86	0.13	-0.71	2.22	-0.14	-1.60	-0.48	-0.78	0.56	-0.03	0.12	0.06	-0.57	0.00	-0.70	-0.47	-0.43	-0.66	-0.65	-0.26	0.34	-0.69	0.32	0.77	0.32	-0.86	-0.86	0.14	-0.10	-0.42	-0.44
mml-miR-19a-5p	0.53	0.00	-0.07	0.07	0.79	0.00	0.00	3.36	0.00	0.48	-0.35	0.00	0.06	0.00	-0.38	1.50	0.44	0.76	0.00	1.14	0.00	0.52	3.02	0.27	0.96	0.00	-0.45	0.41	0.93	0.00	0.00	0.82	3.00	0.45	0.00	0.28
mml-miR-200a-3p	3.78	1.97	2.88	-0.03	1.30	0.00	3.84	1.82	0.00	-0.29	0.68	2.96	0.98	0.94	0.04	-0.30	0.99	0.00	0.00	0.18	4.23	-0.32	0.36	3.27	2.07	1.88	2.24	-1.00	2.12	0.00	2.21	0.97	2.00	0.43	0.11	0.00
mml-miR-203	0.22	0.24	0.87	0.17	0.85	0.00	-0.38	-0.25	0.00	-0.12	-0.80	-0.06	1.14	3.60	0.20	-0.22	0.40	1.17	1.55	0.68	0.14	-0.88	-0.25	0.63	0.54	1.51	0.12	0.21	0.98	-0.37	0.00	-0.09	0.91	-0.89	-0.38	-0.33
mml-miR-205	0.00	0.90	0.97	-0.20	1.19	0.00	-1.23	-1.54	0.00	0.47	-0.92	0.21	0.43	0.48	0.90	0.66	1.88	1.86	0.96	-0.13	1.15	0.90	0.14	0.88	-0.82	0.64	0.17	0.09	0.46	0.73	0.00	-0.66	0.84	-0.69	-0.21	0.46
mml-miR-20a-5p	-1.31	1.47	0.00	0.12	0.68	0.57	0.88	0.63	0.00	-0.30	-0.44	-0.87	-1.35	1.52	-0.68	-0.07	0.04	0.22	1.22	0.23	0.85	0.09	0.26	-0.09	-0.20	0.32	0.05	0.65	-0.09	0.38	1.15	-0.95	0.00	-0.18	-0.02	0.02
mml-miR-21-3p	1.33	0.23	-0.22	-0.70	0.13	-1.00	-0.19	1.02	-1.20	0.70	0.46	0.20	1.60	0.02	-0.11	0.19	-0.35	0.84	0.98	0.73	-1.26	0.96	-0.07	1.64	0.57	0.55	-0.53	0.02	0.65	0.05	-0.07	2.52	0.56	1.22	0.22	0.11
mml-miR-21-5p	-0.12	-1.30	-0.55	-0.88	-0.61	-0.51	-0.53	0.70	1.81	-0.43	-0.31	0.57	-0.58	-0.34	-0.92	0.44	-0.30	0.27	-0.74	-0.78	-0.56	-0.39	-0.56	0.26	-0.13	-0.83	-1.15	0.15	0.21	0.15	-0.58	0.27	0.10	0.08	-0.92	-0.02
mml-miR-210-3p	-0.49	1.13	0.47	-0.36	-0.48	-0.27	1.18	0.19	0.00	0.48	0.79	-0.48	-1.02	-0.42	-0.67	-0.12	-0.72	0.09	0.70	0.04	0.32	0.50	0.04	-0.50	-0.41	0.05	-0.73	0.00	-0.37	-0.52	-1.18	0.08	-0.45	0.49	-0.68	-1.23
mml-miR-210-5p	-1.19	1.97	-1.43	1.36	0.26	2.57	0.03	-1.34	0.00	-0.20	-0.80	-1.08	-0.09	2.95	0.51	0.32	-0.73	0.00	0.00	0.36	0.00	1.12	0.06	-2.47	-1.06	-0.08	0.05	0.07	-0.21	0.00	0.00	-0.08	0.00	0.87	0.36	-0.61
mml-miR-214-3p	-0.05	0.81	0.25	-0.37	0.65	0.00	0.00	-1.42	0.00	-0.11	-1.05	-0.87	0.32	1.07	0.35	-0.03	0.50	-0.09	0.00	0.13	1.12	-0.79	-0.04	-1.35	-0.03	1.14	0.79	-0.12	0.75	1.04	0.00	-0.32	2.17	-0.35	0.35	-1.12
mml-miR-215-5p	-1.80	-1.26	-0.55	-1.74	-1.00	-0.96	-4.83	-1.38	-0.87	-0.90	-0.62	-2.70	-2.21	-0.19	0.76	1.14	-0.08	0.08	-2.92	-0.29	0.71	-0.81	0.03	-0.64	-1.12	0.71	0.56	0.22	0.03	0.01	-3.50	-1.35	-0.01	-1.35	-0.85	-0.15
mml-miR-22	0.28	0.26	0.02	-0.71	-0.16	-0.68	0.77	0.39	-0.68	0.35	-0.31	-0.17	0.28	0.44	0.07	0.20	-0.24	0.26	0.27	-0.33	0.16	0.09	-0.30	0.25	0.59	-0.45	-0.43	0.21	0.19	0.27	-0.24	0.57	0.13	0.42	-1.01	-0.17
mml-miR-221-3p	-0.35	0.74	-0.88	-0.99	-0.03	-0.42	-0.39	0.21	-1.63	0.06	-1.40	-0.36	-0.56	0.21	-0.41	0.20	-0.07	0.10	0.22	-0.10	0.10	0.10	-0.72	0.00	-0.37	-1.11	-1.66	0.02	0.43	0.50	-0.02	0.50	-0.03	0.16	-1.06	-0.97
mml-miR-223	-0.01	-1.83	-0.81	-0.69	-0.86	-0.74	0.86	0.71	-1.84	0.45	-0.67	0.59	0.30	0.03	-0.96	-0.28	-0.31	0.43	-0.91	-0.12	-0.51	-0.59	-0.15	-0.68	0.49	0.27	-1.38	-0.21	-0.48	0.18	1.54	0.53	0.00	0.60	-1.07	-1.52
mml-miR-23a-3p	0.29	0.63	-0.68	-0.72	-0.24	-0.75	0.58	0.45	-1.78	0.25	-0.34	-0.36	0.04	1.22	-0.05	0.17	0.09	0.09	0.15	0.07	0.12	-0.16	-0.11	-0.12	0.21	0.13	-0.64	-0.15	0.07	0.65	0.47	1.01	-0.35	0.60	0.43	-0.29
mml-miR-23b-3p	-1.10	2.63	-0.13	0.13	0.10	0.00	0.22	0.37	1.47	0.20	-0.33	0.21	-0.50	4.02	0.22	0.09	0.94	-1.56	-2.26	-0.02	0.62	-1.03	0.26	0.17	-0.46	1.71	0.72	0.38	0.03	0.89	-0.59	0.32	0.41	0.03	0.20	0.13
mml-miR-25	-0.24	1.04	-0.33	0.04	0.33	0.13	-0.24	0.18	-0.60	-0.41	-0.13	-1.04	-0.73	0.25	0.35	-0.20	0.08	-0.79	0.86	-0.36	1.01	0.24	-0.13	-0.25	0.22	-0.80	-0.09	0.01	0.38	-0.06	0.22	-0.53	0.11	-0.35	-0.22	-0.83
mml-miR-26a-5p	-0.64	-0.06	-0.54	-0.38	-0.63	-1.12	0.39	-0.36	1.04	-0.27	-2.45	-0.20	-0.20	0.94	-0.35	0.33	-0.23	0.34	-0.83	-0.80	-0.38	-0.76	-0.85	0.19	-0.18	0.34	-0.20	0.07	-0.56	0.23	0.17	-0.14	-0.55	-0.76	-1.47	-0.60
mml-miR-26b-5p	-0.48	-0.11	0.30	-0.02	-0.76	-0.50	-0.85	0.00	-0.36	-0.39	-1.28	0.13	-0.66	0.74	-0.10	0.41	-0.29	0.34	-0.92	-0.89	-0.35	-0.47	-0.01	0.10	-0.41	0.51	-1.79	-0.23	-0.53	-0.21	-1.74	-0.23	-1.22	-0.70	-0.41	-0.41
mml-miR-27b-3p	0.36	0.23	-0.30	-1.12	0.03	0.28	-0.48	0.73	-1.81	0.31	0.08	0.02	-0.29	0.90	0.42	0.73	-0.03	0.71	-0.43	-0.35	0.65	0.27	-0.14	1.09	0.13	-0.40	-0.65	0.36	0.65	0.91	-0.06	1.32	0.75	0.39	-0.90	0.34
mml-miR-28-3p	-0.10	-0.15	-0.34	-0.25	-0.27	-1.15	-0.40	0.13	0.68	-0.58	-0.64	-0.02	-0.18	0.32	-0.58	0.18	-0.20	-0.02	-0.76	0.08	-1.40	-0.62	-0.12	-0.51	-0.19	-0.12	-0.04	-0.01	-0.19	-0.94	0.03	0.24	-0.64	-0.08	0.02	-1.10
mml-miR-296-5p	-0.27	1.87	0.00	1.24	-0.53	0.00	0.00	-0.60	0.00	2.74	0.25	-0.06	0.05	2.55	0.00	0.49	0.82	0.76	0.00	0.75	-0.16	1.53	1.59	-0.88	-1.63	0.21	0.00	-0.65	-0.87	2.21	0.00	0.77	-0.91	0.85	0.18	0.14
mml-miR-29a-3p	-0.03	1.61	1.09	-1.86	-0.24	1.26	1.91	0.38	3.65	0.79	0.34	0.10	-0.17	0.88	1.08	-0.48	0.48	1.54	0.39	-0.42	1.08	-0.43	1.84	0.31	-0.41	0.25	0.16	-1.08	0.11	2.02	-0.11	0.19	0.22	-0.17	0.67	0.41
mml-miR-29c-3p	0.25	1.31	1.51	-0.09	-0.05	1.78	1.57	0.64	1.47	1.11	-0.05	-0.42	-0.16	-0.35	0.65	-0.06	-0.33	0.85	1.42	-0.17	1.55	0.08	0.46	0.18	-0.19	-0.29	-0.65	-0.62	-0.29	1.19	-1.77	-0.05	0.66	0.05	-0.15	0.19
mml-miR-301a-3p	-0.95	0.22	0.33	0.41	-0.31	0.00	-1.71	0.01	0.96	-1.59	0.01	-0.82	0.17	0.19	-0.11	0.48	-0.74	-0.54	-0.45	-0.25	-0.37	-0.75	-0.14	-0.48	-0.44	0.53	-0.28	-0.03	-0.86	-1.00	-0.26	-1.01	-0.70	-0.21	-0.84	-0.42
mml-miR-301b	0.16	0.74	0.56	1.31	0.11	0.00	-1.55	0.00	0.00	0.82	0.35	2.59	1.03	2.06	0.00	1.83	0.89	2.34	0.00	0.00	0.00	-0.06	-0.28	1.34	0.48	1.71	0.62	0.47	1.57	2.21	0.63	0.00	0.00	0.62	-0.39	0.75
mml-miR-30a-3p	0.08	1.40	0.28	0.19	0.76	0.00	-0.67	1.85	2.25	0.12	1.77	1.33	-0.56	2.46	0.19	1.22	1.03	1.34	-0.57	1.29	0.30	-0.54	-0.24	1.57	-0.28	0.85	0.26	0.53	0.93	2.21	-2.81	1.70	-0.21	-0.07	-0.82	1.68
mml-miR-30a-5p	0.09	1.59	0.75	-0.25	0.78	0.53	0.93	0.65	-2.33	0.34	0.90	0.28	-0.30	1.01	1.16	0.29	0.47	0.78	0.24	0.41	1.21	0.51	0.52	0.85	-0.23	0.61	0.85	0.32	1.22	1.10	0.84	0.88	0.86	0.58	0.55	0.74
mml-miR-30b-5p	-0.20	-0.34	0.52	0.04	-0.64	0.00	-0.27	0.21	0.00	-0.03	-0.16	-0.83	-0.02	-1.38	-0.88	-0.02	0.19	-0.80	-0.37	0.05	-0.40	0.06	0.68	-0.98	-0.07	-0.58	0.87	-0.01	-0.98	-1.27	-2.52	-1.25	-0.45	-0.47	-1.20	-0.69
mml-miR-30c-5p	-0.80	-3.66	-0.49	1.39	-0.63	-0.21	0.78	-1.03	0.42	-0.04	0.21	0.06	-0.39	-1.28	-0.48	0.45	-0.03	0.50	-0.31	-0.79	-0.14	-0.33	-0.33	-0.38	0.28	-1.49	0.35	-0.38	-1.07	0.64	-1.09	-1.09	-1.07	-0.71	-1.01	0.38
mml-miR-30d-3p	0.49	-0.07	0.57	0.15	0.36	0.00	1.62	2.27	0.00	0.04	0.00	1.42	0.55	0.32	-0.09	-0.39	-0.06	0.76	1.55	0.90	-1.58	0.44	0.21	0.98	-1.03	0.47	0.57	0.34	0.06	-1.11	3.06	1.69	-1.33	-0.24	0.08	0.63
mml-miR-30d-5p	-0.15	-0.02	-0.42	0.02	-0.05	-0.63	0.10	-0.03	-0.56	-0.16	0.02	-0.03	-0.02	-0.29	-0.04	-0.03	-0.23	-0.08	-0.87	-0.23	-0.70	-0.19	0.13	-0.16	0.40	-1.47	0.10	0.10	0.11	-0.15	-0.34	-0.02	-0.64	-0.07	-0.73	-0.44
mml-miR-30e-5p	-0.39	0.87	-0.09	-0.64	0.24	-0.46	0.46	0.05	-0.61	-0.22	-1.09	-0.76	-0.38	0.62	-0.29	-0.07	-0.29	-0.34	-0.83	-0.36	-0.02	-0.22	0.06	-0.59	0.03	-0.26	-0.98	0.25	0.23	0.27	0.46	-0.15	-0.06	-0.24	0.08	-0.78
mml-miR-3122	0.53	1.26	0.78	-0.15	0.31	0.00	-0.23	1.09	0.00	-1.02	-0.45	-0.85	0.62	0.55	0.21	-1.02	0.68	0.02	2.13	1.06	3.13	-0.53	-0.97	0.27	0.72	0.27	0.24	-0.40	0.12	0.00	0.11	0.33	1.12	-1.09	1.11	-0.66
mml-miR-320a	-0.34	0.30	-0.21	0.77	0.05	0.13	0.33	-0.29	-0.20	-0.14	0.37	-0.47	-0.45	-0.09	-0.31	0.34	0.34	-0.77	1.12	-0.28	0.63	0.29	0.12	0.06	0.27	-0.63	0.10	0.08	-0.05	-0.35	-0.94	-0.87	-0.04	-0.09	-0.38	-0.12
mml-miR-320b	0.30	1.29	0.44	-0.27	0.70	-0.08	0.32	-0.39	-0.31	-0.62	0.06	-1.05	0.02	0.24	0.33	0.62	0.59	0.51	0.79	-0.18	1.43	0.21	1.15	0.87	0.21	0.44	-0.27	0.55	0.42	0.93	-1.50	-0.18	1.61	-0.04	0.04	0.58

mml-miR-323b-3p	0.99	-0.74	-0.36	0.12	-0.56	0.00	-1.53	0.02	0.00	0.08	0.16	0.11	0.57	-0.03	-1.39	0.02	-0.46	-0.48	-0.60	0.15	-2.09	0.28	-0.61	-1.08	0.15	-0.31	-1.10	0.05	-0.79	-1.52	-0.80	-0.14	-1.93	0.86	-0.74	-1.33
mml-miR-324-3p	-0.36	3.22	-0.70	1.92	0.28	0.00	0.77	-2.11	4.02	1.17	-0.84	0.42	-0.30	2.09	-0.03	0.11	0.23	-0.24	0.00	-0.70	0.31	1.15	0.70	-0.61	0.54	0.29	1.07	0.15	-0.49	0.00	0.00	0.03	3.20	0.21	3.44	0.24
mml-miR-328	-1.29	5.57	0.19	2.30	-0.35	3.57	1.84	-1.75	2.87	1.25	0.77	1.17	-1.11	5.75	-0.06	1.02	-0.06	2.08	0.55	-0.93	0.39	0.71	-0.43	0.27	-0.51	1.38	1.46	0.22	-0.86	1.21	2.35	-0.09	1.90	0.16	0.59	1.25
mml-miR-331-3p	-0.66	0.65	0.36	2.08	-0.06	0.00	0.45	-0.98	0.00	0.16	-0.22	0.45	-1.11	0.39	0.68	0.97	-0.85	-0.83	-0.04	0.09	0.14	0.54	0.11	-1.73	-0.19	1.91	2.09	0.29	-0.53	0.21	0.00	-0.71	-0.35	0.02	-0.03	0.43
mml-miR-331-5p	-0.57	2.20	0.64	0.04	1.02	0.00	-0.97	0.22	0.00	0.06	-0.19	-0.97	-0.29	-0.10	0.72	0.22	0.66	0.50	1.08	1.00	0.71	0.16	-0.25	-0.35	-0.66	1.69	0.45	0.29	0.46	1.09	-1.28	0.43	-0.20	0.30	0.64	-1.03
mml-miR-337-3p	0.69	0.00	0.29	0.22	0.59	0.00	-1.55	0.00	0.00	0.47	0.04	1.76	1.83	0.90	0.07	-0.07	1.34	0.00	0.00	0.00	-2.16	0.69	0.53	0.49	0.79	0.51	0.11	0.30	1.02	0.00	0.63	0.00	-1.59	1.25	-0.21	0.90
mml-miR-338-5p	0.65	0.24	0.45	0.49	0.41	0.00	0.00	1.96	0.00	0.52	-0.88	-0.15	1.10	1.52	0.15	0.34	0.34	0.00	0.00	2.54	0.00	0.09	-0.40	0.09	1.73	0.71	1.33	-0.45	0.04	2.80	0.00	0.67	-0.59	0.00	0.00	1.44
mml-miR-340-5p	-0.39	-1.83	-0.11	-1.14	-0.92	-0.73	-0.47	0.53	0.15	0.02	0.02	0.58	-1.03	-2.45	-1.37	0.18	-0.50	-0.73	-0.56	0.14	-1.18	0.23	-0.06	0.00	-0.73	0.05	-1.31	0.13	-0.35	-1.85	-1.19	-0.17	-0.63	0.56	-0.86	-0.49
mml-miR-342-3p	0.32	2.52	0.61	0.19	-0.80	1.11	-0.38	0.70	4.42	-0.24	0.34	-1.55	0.13	1.43	-0.29	-0.32	-0.56	-0.70	-2.26	0.26	-0.04	-1.44	-0.21	-2.27	0.01	0.34	-0.57	-1.00	-1.41	0.56	-3.92	-1.23	-0.88	-1.78	-0.71	-1.68
mml-miR-342-5p	0.93	-0.26	0.43	0.42	-0.43	0.00	0.00	-0.84	3.89	0.25	-0.91	-0.52	0.06	1.69	-0.80	0.19	-0.15	-0.24	0.00	-0.54	0.00	-0.07	0.00	-0.74	1.52	0.71	0.55	0.24	-0.65	0.00	-0.59	-0.16	-1.00	-0.39	-0.53	-0.18
mml-miR-34a-5p	1.64	1.15	3.21	-0.02	2.27	0.00	1.03	0.87	0.00	0.56	0.58	2.02	0.89	-0.06	3.04	-0.26	1.26	0.00	0.00	0.31	0.00	0.53	0.20	2.71	1.86	1.71	5.52	-0.09	4.88	0.00	0.89	2.95	0.00	0.86	0.51	0.00
mml-miR-361-3p	-1.56	1.71	-0.10	0.52	-0.36	2.40	-0.50	0.39	0.48	0.08	-0.20	-0.02	-1.29	0.40	-0.71	0.58	0.02	-0.83	-1.15	-0.10	-1.15	-1.07	-0.92	0.74	-0.83	2.28	0.84	0.58	-1.57	-0.96	-1.81	-0.13	-0.11	-0.99	-0.44	0.30
mml-miR-362-3p	-0.16	0.91	0.00	0.03	0.65	0.00	0.00	0.67	0.00	0.97	0.32	0.67	0.34	1.02	0.00	0.56	1.28	0.00	0.00	1.38	3.01	0.27	1.07	2.45	-0.38	0.21	0.00	-0.56	0.66	2.80	0.00	0.00	0.00	0.14	-0.06	2.10
mml-miR-363-3p	-0.37	-1.62	-0.83	1.12	-0.48	2.48	0.17	-0.58	1.49	0.06	0.19	0.87	0.25	-1.44	-0.69	-0.59	-1.20	1.37	-0.78	-1.12	0.13	-0.58	0.82	0.61	0.81	-1.37	-0.19	-0.41	-0.80	-0.29	0.66	-0.47	-0.54	-0.44	1.19	0.19
mml-miR-369-3p	0.00	-2.51	0.06	-1.32	-1.16	-1.17	-0.96	0.38	0.00	-0.03	1.19	-0.04	0.10	-0.82	-0.66	-0.53	-0.77	-1.03	-0.95	-0.35	-1.91	0.36	-0.44	-1.19	-0.87	-0.64	-1.03	-1.21	-0.84	-2.36	-1.71	-0.10	-0.96	1.40	-1.20	-0.20
mml-miR-369-5p	-0.47	-1.00	-0.95	0.04	0.06	0.00	-0.55	0.67	0.00	-0.48	-0.36	0.33	0.26	-0.32	-0.69	0.10	-0.44	-0.73	0.00	0.79	-5.38	-0.60	0.11	-1.35	-0.81	-0.04	-0.20	0.19	-0.56	-2.60	2.48	1.43	-2.81	-0.56	-0.42	-0.94
mml-miR-374b-5p	-0.02	3.96	0.99	0.05	0.00	0.00	0.00	1.56	0.00	-0.16	-1.16	0.60	0.22	1.60	0.90	0.86	-0.63	0.00	0.00	0.00	1.01	0.08	-0.11	-0.36	0.51	1.51	-0.03	0.82	-0.06	2.80	0.00	0.88	0.00	0.00	-0.61	0.95
mml-miR-375	3.32	4.86	4.18	1.59	2.82	5.99	4.99	2.67	0.00	1.85	3.09	3.21	0.79	1.35	1.87	0.46	0.64	3.57	1.97	0.28	4.52	0.27	2.05	1.70	-0.33	0.95	1.31	0.32	1.24	2.74	0.46	0.92	1.81	1.00	0.99	1.77
mml-miR-376a-3p	0.30	0.24	-0.03	0.14	-0.17	0.00	0.00	0.10	0.00	0.61	-0.31	-0.42	1.00	1.23	-0.67	1.36	-0.60	0.00	0.00	0.03	-0.28	0.77	-0.81	-2.37	0.65	0.51	0.03	1.15	-0.80	0.21	0.00	0.87	0.30	0.86	-0.12	-0.31
mml-miR-376b-3p	2.04	0.00	-0.02	0.11	1.29	0.00	-1.55	0.00	0.00	0.21	0.49	1.76	1.58	0.00	-0.55	-0.14	0.00	0.00	0.00	0.00	0.00	1.32	0.12	0.34	0.57	0.00	0.22	0.73	1.35	0.00	0.00	0.00	0.00	2.22	0.21	0.48
mml-miR-376c-3p	0.79	0.51	0.53	0.21	0.08	0.00	1.45	1.24	0.00	-0.18	0.18	-0.48	1.32	1.23	-0.34	0.12	0.00	-0.90	-0.04	1.11	-1.99	-0.27	-0.02	-1.98	1.43	0.71	0.31	0.27	-0.24	-1.04	1.89	1.88	-0.68	0.98	-0.33	-0.80
mml-miR-377-3p	1.56	3.85	1.18	2.19	0.84	0.00	1.35	1.52	0.00	0.00	-0.62	-0.83	1.47	3.69	0.14	1.74	0.39	2.34	0.55	1.48	-1.10	0.48	-0.88	-1.18	1.77	2.01	0.32	1.19	1.17	0.00	3.06	2.93	-1.53	0.11	0.28	-0.27
mml-miR-378a	0.15	-0.11	0.50	-0.87	-0.48	-0.60	0.03	0.20	-2.26	0.68	0.68	0.24	-0.82	-0.56	0.17	0.08	-0.17	-1.16	0.32	-1.21	0.19	0.24	0.38	-0.07	-0.51	-0.61	-0.03	-0.15	0.16	-1.38	-1.43	-0.79	-0.42	-0.24	-0.80	0.27
mml-miR-378d	0.26	0.26	0.69	-0.26	-0.21	0.25	0.60	0.51	-1.20	0.88	0.71	0.00	-0.14	-0.35	0.62	0.23	0.10	-0.21	0.87	-0.65	0.38	0.29	0.44	0.27	-0.09	0.32	0.41	0.07	-0.14	-0.89	-1.34	-0.47	-0.11	-0.24	-0.87	0.56
mml-miR-380-3p	-0.31	-2.74	0.18	-1.10	-1.00	-2.76	-1.86	0.35	-7.83	-0.91	0.63	0.47	-1.07	-1.09	-0.80	-0.72	-0.87	-1.06	-2.06	-0.64	-2.22	0.07	-0.54	-1.17	-0.63	-1.13	-0.92	-0.65	-0.62	-2.35	-1.51	-0.49	-1.90	0.46	-2.09	-1.88
mml-miR-381-3p	0.28	-0.62	0.13	-1.98	-0.88	-1.71	-0.77	0.66	-7.79	-0.07	0.63	0.88	0.17	-0.57	-1.21	-0.90	-0.59	-0.22	-0.99	0.36	-2.33	0.39	-0.29	0.75	0.00	-0.17	-0.95	-0.69	-0.77	-1.56	-0.93	1.28	-0.13	1.23	-1.02	0.49
mml-miR-409-3p	0.19	-1.61	-0.86	-0.93	-0.75	-3.73	-0.93	-0.26	-4.67	-0.68	0.28	0.80	-0.57	-0.43	-1.61	-0.08	-0.96	-0.13	-1.12	-0.76	-2.65	-0.06	-0.87	-0.82	-0.22	0.05	-0.91	0.21	-0.41	-1.25	1.11	0.23	-1.38	0.47	-0.49	-1.24
mml-miR-410-3p	0.68	-1.96	-0.39	-1.76	-0.70	-3.22	-1.13	0.32	-2.83	-0.51	0.35	0.94	0.69	-0.87	-1.42	-0.12	-0.79	0.02	-1.44	-0.80	-2.90	0.08	-0.26	0.31	0.61	-0.60	-0.75	-0.23	-0.30	-1.16	-0.06	0.38	-1.47	0.57	-1.73	-0.63
mml-miR-411-5p	-0.33	-1.20	0.08	-0.25	-0.86	-2.74	-0.88	-0.21	-3.49	-0.32	0.13	0.62	0.46	0.49	-1.86	-0.06	-0.38	-0.02	-1.59	-0.11	-1.47	-0.05	-0.31	0.19	0.02	0.61	-0.84	-0.06	-0.30	-0.84	-0.45	0.37	-0.20	-0.02	-0.18	-1.26
mml-miR-421	0.13	-0.60	-1.99	-0.77	-0.42	-2.29	-0.82	-0.19	1.13	-0.43	-0.76	0.31	0.40	0.27	-1.48	-0.58	-0.71	-0.46	-0.62	0.02	-0.04	-0.26	0.09	0.00	-0.25	-0.01	-0.33	-0.75	-0.06	-1.19	0.18	-0.37	-0.24	0.09	-0.22	-0.25
mml-miR-423-3p	-0.19	0.92	-0.46	1.30	-0.53	-0.23	1.34	-0.41	-1.96	0.86	-0.10	0.04	0.33	0.84	-0.17	-0.34	-0.60	0.84	1.52	0.51	-0.60	0.51	0.58	0.36	0.59	0.59	0.18	-0.16	-0.76	-0.19	1.29	0.53	-0.34	0.13	-0.23	0.30
mml-miR-423-5p	-0.16	0.52	-0.72	0.34	0.53	-1.34	-0.33	-0.79	-0.32	-0.73	-0.22	-1.42	-0.21	-0.31	0.19	-0.50	-0.12	0.02	0.46	0.16	0.68	-0.07	0.90	-0.16	0.51	-0.11	0.63	0.02	0.05	0.61	0.82	0.22	0.39	-0.52	0.72	-0.70
mml-miR-424-3p	1.05	1.74	0.82	-0.16	2.38	0.00	1.62	0.54	0.00	-0.38	-0.33	0.16	0.06	3.92	0.57	1.13	1.95	0.00	0.00	0.12	3.30	0.25	-0.96	-0.77	1.10	0.00	1.41	0.14	1.03	0.00	0.00	0.00	3.00	-0.25	0.00	-0.57
mml-miR-424-5p	0.28	3.88	1.48	0.06	0.68	3.57	2.03	0.43	1.88	0.56	1.23	0.66	0.27	3.42	0.44	0.18	-0.15	2.34	1.55	-0.15	1.42	-0.93	0.04	0.08	0.11	4.92	1.31	-0.56	0.00	3.09	0.00	-0.30	-2.43	-0.56	-0.59	0.35
mml-miR-425	-0.57	0.20	-0.76	0.45	0.18	-0.44	0.17	0.01	-3.60	-0.36	-0.16	-0.98	-0.38	-0.12	-0.63	-0.03	-0.14	-0.16	0.24	-0.42	0.10	-0.39	-0.55	-0.09	0.31	0.02	0.32	0.13	-0.11	0.33	0.04	-0.47	-0.54	-0.59	-0.85	-0.13
mml-miR-429-3p	0.65	1.97	2.06	-0.75	0.50	0.98	0.96	2.19	0.00	1.80	0.74	1.75	0.22	2.35	1.05	-0.96	-0.04	-0.24	-1.78	0.26	3.10	-0.01	-0.31	1.30	0.02	1.21	1.53	-1.36	0.60	1.21	-1.43	0.63	1.67	2.06	1.24	0.52
mml-miR-433-3p	0.37	0.64	-0.92	1.07	-0.20	2.98	-0.48	0.25	1.86	0.23	0.18	-0.33	0.99	1.39	-0.26	-0.41	-0.19	1.98	-0.78	-0.05	-2.58	-0.30	-0.36	-1.58	-0.15	0.35	2.07	1.24	-0.29	0.00	-0.26	0.47	0.13	0.88	0.73	0.27
mml-miR-4446-3p	0.50	-0.99	-1.61	-0.15	-0.72	0.00	-0.47	-0.92	0.00	-0.54	-0.69	-0.04	0.94	-0.25	-1.05	-0.39	-1.03	-0.55	-1.45	-0.19	-2.79	-0.30	-0.11	-0.41	0.69	0.39	-0.12	0.20	-0.65	-1.04	1.55	0.16	-0.45	0.03	-0.33	0.06
mml-miR-451	-0.15	1.11	-0.07	-0.38	0.86	0.78	0.83	-0.10	0.31	-0.48	-1.10	-2.32	-0.59	-0.13	0.33	-0.67	-0.21	-0.83	0.79	-0.48	1.49	-0.41	0.26	-0.80	0.75	-0.89	-0.48	0.13	0.50	1.33	1.97	0.01	0.59	-0.34	-0.94	-1.24
mml-miR-452-5p	0.04	0.24	0.00	0.49	0.00	0.00	1.03	2.35	0.00	0.49	-0.25	0.60	0.08	1.10	0.00	1.71	0.90	0.00	0.55	0.84	1.10	0.80	0.10	2.64	1.01	1.88	0.00	0.92	1.11	0.00	0.00	0.70	0.00	-0.34	-0.39	0.87
mml-miR-484	-0.63	0.94	-1.10	0.56	-0.07	0.44	0.70	-0.80	-0.25	0.73	-0.57	-0.93	-0.50	0.19	0.05	-0.87	-0.38	0.71	0.13	-0.16	0.38	0.19	0.12	-0.24	0.47	-2.20	-0.12	-0.28	-0.42	0.11	2.06	0.28	0.19	-0.16	-0.04	-0.62
mml-miR-485-3p	0.89	-1.26	1.53	0.66	-0.40	0.00	-1.43	0.91	0.00	1.28	0.71	1.14	0.25	-0.39	0.00	-0.48	-0.67	-0.05	-0.33	0.06	-1.81	0.55	-0.43	-0.88	0.36	0.15	2.72	0.53	-0.93	-1.60	0.00	0.50	-2.56	1.77	-0.05	0.35
mml-miR-485-5p	0.56	0.13	-1.00	0.64	0.87	0.00	0.00	-0.55	0.00	-0.49	-0.80	-0.32	0.89	0.48	0.00	0.40	-0.31	-0.98	0.00	0.16	-2.20	-0.62	-0.18	-3.03	0.78	0.40	1.04	1.16	0.71	-1.11	1.89	1.03	-0.26	-0.23	-0.94	-1.15
mml-miR-486-5p	-1.11	-0.13	-1.19	1.14	0.77	1.56	-0.90	-1.48	0.68	0.12	-0.41	-1.50	-1.09	-1.54	-0.13	-0.55	0.11	-0.85	0.47	-0.19	0.57	0.13	1.05	-0.32	-0.06	-1.26	0.22	-0.20	-0.58	-0.03	1.59	-0.63	1.10	-0.57	1.81	-0.28
mml-miR-487b-3p	-0.94	-0.48	0.34	-0.73	-0.53	-1.52	-0.68	0.41	0.00	-0.09	-0.41	0.71	0.28	-1.07	-0.68	-0.19	-0.45	0.13	-2.28	0.03	-1.42	0.01	-0.05	-1.01	-0.05	-0.10	0.43	0.35	-0.75	-2.29	-1.61	0.22	-0.51	0.70	-0.16	-0.37
mml-miR-493-3p	0.61	-0.12	-0.09	0.26	-0.36	0.00	0.20	0.21	0.00	-0.12	0.45	1.17	0.75	0.25	0.65	0.15	-0.27	0.50	0.00	-0.08	-1.60	-0.27	-0.15	0.01	-0.07	0.60	0.25	0.37	0.44	-0.37	-0.30	0.29	-0.19	-0.41	-0.03	0.43
mml-miR-493-5p	0.24	-0.14	-1.44	-0.10	-0.33	-0.24	-1.88	-0.23	0.00	-0.05	0.21	0.60	0.32	0.20	-0.31	-0.20	-0.51	-1.05	-0.78	-0.38	-1.87	0.18	-0.56	-0.52	-0.07	0.59	0.67	0.38	-0.08	0.00	-1.43	0.65	-2.03	0.45	-0.13	0.80
mml-miR-499-5p	1.00	4.22	1.11	1.74	0.60	0.00	0.00	1.32	0.00	0.95	0.62	0.42	0.27	0.44	1.05	0.14	0.40	0.00	0.00	0.52	2.01	0.07	0.39	-0.79	-0.33	1.21	-0.25	0.31	0.43	0.00	0.00	0.00	0.00	0.00	1.44	2.24
mml-miR-500a-3p	1.44	-1.46	-0.50	0.55	0.00	1.76	1.62	1.88	0.00	1.68	0.42	-0.88	2.24	-1.18	0.41	0.10	0.09	0.27	-0.04	2.09	-0.83	-0.10	1.44	0.65	1.16	-0.49	-0.13	-0.22	-0.15	0.99	0.63	3.10	-2.25	0.56	-0.06	0.21
mml-miR-505-3p	0.18	0.63	0.60	0.35	0.22	0.00	2.84	-0.53	0.00	0.46	0.13	0.71	-0.11	0.82	0.56	0.38	0.91	-1.41	0.55	-0.46	1.79	-0.12	-0.27	-0.14	0.57	-0.45	0.82	-0.03	-0.30	0.00	-1.11	0.00	0.04	0.36	-0.54	0.24
mml-miR-532-3p	0.12	-0.98	-1.35	2.41	-0.22	0.00	1.62	-1.87	0.00	1.43	-0.92	0.19	-0.26	-1.55	0.30	0.77	0.27	-0.66	2.13	-1.04	0.67	1.12	-0.37	-0.50	0.91	-1.73	0.81	0.70	-1.04	0.80	0.00	-0.97	1.51	0.79	0.54	1.02
mml-miR-543-3p	-1.49	-0.17	-0.44	0.42	-0.01	0.00	0.62	0.21	0.00	0.71	-0.07	-0.06	-0.06	0.83	-0.69	0.21	0.49	-0.46	-1.62	0.80	-3.23	0.69	-0.53	-0.80	-0.03	0.59	0.19	0.17	-0.35	-1.60	-0.19	0.48	-1.17	1.51	-1.00	-0.26
mml-miR-548j-3p	2.49	0.00	-0.01	-0.16	1.00	0.00	0.00	0.49	0.00	0.62	-0.03	-0.50	0.39	0.00	0.39	-1.28	-0.55	0.00	0.00	0.34	1.17	0.08	0.23	0.96	0.38	0.00	-0.54	-1.63	-0.16	1.21	-0.11	1.06	0.00	-0.29	0.14	0.22
mml-miR-550-3p	-0.86	-1.35	-0.13	0.64	0.83	0.00	0.00	-0.01	0.00	0.59	0.05	0.27	-1.40	-2.59	0.17	-0.17	-0.15	0.00	0.00	0.36	1.10	0.56	-0.36	0.89	-0.15	0.00	-0.84	-0.87	-0.45	-0.79	0.00	0.00	0.67	0.27	0.05	0.73
mml-miR-550-5p	0.00	0.24	1.01	1.83	1.62	0.00	0.00	1.54	0.00	1.31	-1.14	0.20	0.20	2.69	1.12	-0.20	0.11	0.76	0.00	0.40	-0.75	1.12	-0.75	0.58	1.29	0.21	2.20	-0.10	0.39	0.00	0.00	0.90	0.00	1.19	-1.19	0.46
mml-miR-574	0.51	0.24	-0.87	0.04	-0.86	1.11	2.14	-0.91	0.00	0.80	-0.36	-1.11	0.17	1.37	0.43	0.47	-0.42	0.62	0.55	-0.94	1.37	-0.48	0.36	-1.30	0.59	0.11	1.11	-0.05	-0.77	1.45	-1.43	-1.02	1.39	-0.09	-0.31	-0.33
mml-miR-584-5p	-0.64	1.77	-0.79	-0.32	0.65	0.62	-1.19	0.26	0.00	-0.11	0.13	-2.26	-0.63	0.52	-0.32	0.04	0.07	-0.29	0.35	0.27	1.30	0.27	0.74	-0.43	0.01	0.43	0.14	0.39	0.93	0.66	-0.52	-0.03	0.58	-0.27	0.41	-1.13
mml-miR-590-3p	0.11	0.32	-1.77	0.03	-0.01	2.25	1.03	0.69	0.00	-0.28	-0.70	1.66	0.44	2.09	-1.16	0.62	-1.10	0.02	0.00	0.12	-0.81	-0.07	-0.03	0.69	0.64	0.86	-0.39	0.59	-0.28	-0.11	3.06	0.41	-1.05	0.90	-0.33	0.73
mml-miR-598-3p	0.46	-1.42	-1.09	-0.03	-0.28	0.00	0.03	0.11	0.00	0.05	0.22	0.35	-0.04	1.80	-1.38	-0.78	-0.03	-0.83	0.00	-0.31	-3.58	0.55	0.08	-1.25	0.34	0.21	-0.87	0.13	-0.32	-1.96	-0.11	-0.21	-1.00	0.82	-0.21	0.25
mml-miR-627-5p	-0.08	0.56	0.11	-0.75	0.64	0.00	-0.86	0.55	0.00	-1.36	0.27	-0.15	-0.37	0.56	0.57	-0.07	-0.02	-0.13	0.17	0.85	0.34	-0.19	0.29	0.11	-0.13	0.24	-0.82	-0.19	0.72	-0.05	-1.00	-0.14	-0.50	-1.06	0.40	-0.89
mml-miR-636	-0.66	1.83	-1.26	2.31	0.81	0.00	0.00	-0.87	4.80	2.06	0.00	0.99	-1.39	3.89	0.17	0.22	0.51	0.00	0.00	-0.03	0.84	1.51	0.40	0.00	-1.64	0.00	-0.50	0.17	-0.60	0.00	0.00	-1.65	2.41	0.69	1.49	2.95
mml-miR-652	-0.35	2.05	-1.22	0.61	-0.10	2.62	0.60	-0.94	0.38	0.13	-1.58	-0.42	-0.12	2.22	0.24	-0.33	-0.75	0.76	1.01	-0.06	0.40	-0.15	-0.13	-0.05	0.53	0.91	0.16	-0.05	-0.53	2.01	0.32	0.00	0.15	-0.41	1.52	-0.26
mml-miR-654-3p	0.32	-2.11	-0.19	-1.84	-1.08	-1.76	-0.62	0.50	-3.15	-0.92	-2.09	0.29	0.44	0.38	-1.24	-0.09	-1.22	0.46	-1.72	0.27	-2.27	-0.36	-0.49	-0.99	0.24	-0.33	-2.06	-0.11	-1.12	-0.77	0.94	0.57	-1.95	-0.11	-1.56	-2.00
mml-miR-654-5p	1.12	0.73	0.23	0.15	-0.17	0.00	-1.55	2.40	0.00	0.42	-0.30	-1.32	0.55	-0.25	-0.44	-0.48	-0.42	0.00	0.00	1.06	-3.22	1.04	-0.28	-1.66	-0.64	0.86	-0.32	0.63	0.38	0.00	0.63	1.73	-0.65	0.92	-0.24	-0.73
mml-miR-656-3p	0.38	0.10	-0.56	-0.31	-0.15	0.00	1.03	0.68	0.00	0.12	-0.52	0.67	1.34	1.22	-0.50	-0.41	-1.19	-1.68	0.09	0.55	-2.08	-0.14	-0.55	-0.74	0.28	0.30	-0.20	-0.30	-0.61	-1.23	0.34	0.39	-0.66	0.73	-0.55	-0.87
mml-miR-665	0.35	0.74	1.66	0.04	0.03	0.00	0.00	0.73	0.00	0.00	-0.10	-0.31	1.64	1.23	1.13	0.63	-0.53	1.34	0.00	0.57	-0.01	0.96	-1.14	-1.67	0.34	1.21	0.82	0.80	0.01	1.80	0.00	1.93	0.00	1.16	-0.28	-1.61
mml-miR-668	0.74	0.00	1.23	0.73	0.15	0.00	0.00	-0.13	0.00	0.96	-0.80	0.36	0.31	-0.06	-0.18	1.33	0.20	1.76	0.00	-0.36	0.00	0.74	-0.09	-0.27	0.63	0.55	0.41	1.47	0.99	0.00	0.00	-0.17	0.00	0.54	-0.36	-0.13
mml-miR-671-3p	-0.61	0.43	0.60	0.40	-0.61	2.57	-1.14	0.36	-2.29	0.78	0.43	0.05	-0.08	0.64	0.31	0.53	0.09	1.08	0.00	-0.20	-1.00	0.13	0.08	0.11	-1.09	2.54	1.10	0.38	-0.79	-0.79	-1.69	0.52	-0.84	-0.14	1.13	-0.18
mml-miR-7186-5p	-0.03	1.50	0.13	-0.55	1.72	-0.60	1.13	1.15	0.00	-0.08	-0.22	-1.19	-0.45	0.95	-0.05	-0.90	0.31	0.59	0.32	0.49	0.32	-0.42	0.16	-1.05	0.57	0.79	-1.30	-1.26	1.10	1.04	1.94	0.94	-0.85	-0.65	0.47	-2.25
mml-miR-7205-3p	-0.20	0.00	0.12	1.79	0.25	0.00	0.00	0.64	0.00	1.09	0.78	0.55	0.25	0.00	0.00	0.54	0.13	0.76	0.00	0.10	0.00	0.15	0.05	1.17	0.07	0.00	-0.59	0.55	0.46	0.21	0.00	1.54	0.00	0.10	0.22	0.48
mml-miR-760	0.85	-0.72	-2.08	0.69	-0.04	0.00	-0.16	-0.08	0.00	0.58	0.00	1.01	0.52	0.80	-0.82	-0.12	-1.77	0.50	-1.45	-0.16	-1.36	0.14	0.44	-0.39	0.97	0.65	-0.29	0.66	-0.47	-0.79	0.00	0.02	-1.78	0.22	-0.82	0.94
mml-miR-873-5p	1.59	0.00	1.20	0.30	1.24	0.00	0.77	1.60	0.00	0.80	1.45	0.51	0.25	0.00	3.47	0.73	1.17	0.00	0.00	0.43	1.58	0.22	0.00	2.09	2.72	0.00	0.63	0.43	0.06	0.00	0.00	0.00	0.49	0.00	0.59	3.82
mml-miR-874-3p	0.57	-0.83	-0.73	0.61	-0.30	1.98	0.77	-0.15	0.00	1.88	0.37	0.89	1.03	-0.80	-0.09	0.92	0.57	1.17	-0.04	0.96	0.88	0.26	1.76	1.49	0.40	-0.57	0.52	-0.04	-0.43	1.21	-0.69	-0.44	-1.25	1.34	0.40	1.63
mml-miR-889-3p	0.63	-1.40	-0.25	-1.43	-0.65	-1.50	-1.27	0.32	0.00	-0.54	0.07	1.07	0.59	-0.40	-2.07	-0.04	-0.79	-0.82	-2.16	-0.04	-1.90	-0.20	-0.58	-0.10	0.29	-0.62	-0.58	-0.14	-0.45	-1.95	0.38	-0.30	-0.83	0.60	-0.65	-0.50
mml-miR-92a-3p	-0.95	-0.53	-0.89	1.74	0.22	1.24	0.61	-1.35	0.70	0.68	0.53	-0.47	-1.02	-1.74	0.04	-0.22	0.00	0.32	1.37	-0.31	0.33	0.52	1.04	0.31	-0.12	-1.32	0.38	0.06	-0.64	0.31	0.54	-0.48	1.00	0.20	0.77	0.95
mml-miR-92b-3p	-1.00	-1.09	-1.00	1.09	0.26	0.97	1.07	-1.15	2.04	0.67	0.61	-0.75	-1.06	-1.90	0.25	-0.69	0.27	1.10	0.46	-0.18	1.00	0.19	1.16	-0.69	-0.39	-1.33	0.44	-0.66	-0.62	-0.44	1.74	-0.14	1.37	-0.16	1.59	-0.27
mml-miR-93-3p	0.50	1.24	2.12	-0.48	1.92	0.25	1.62	-0.15	0.00	-0.24	-0.22	-0.36	-1.63	-0.15	0.87	-0.08	1.61	-1.56	0.00	0.80	-0.47	0.03	-0.07	0.58	-0.09	-0.87	0.73	-0.15	1.92	-0.52	1.48	-0.16	0.88	-1.18	-0.13	0.69
mml-miR-942-5p	-0.21	1.81	-0.81	-0.33	0.93	1.98	-1.19	0.39	0.00	-0.27	-0.36	-0.58	-0.36	2.33	0.04	-0.69	0.79	-0.24	0.32	0.74	1.63	-0.57	-0.15	-0.80	-0.01	-0.22	0.42	-0.62	0.83	1.63	1.08	-0.45	1.20	-0.07	-0.09	-1.00
mml-miR-98	-0.72	-0.26	-0.78	0.12	-0.66	0.00	0.03	0.47	0.54	-0.76	-0.32	1.07	-0.78	-0.18	-0.66	0.24	0.01	-1.15	-2.91	0.31	-2.00	-0.09	-0.16	0.16	-1.58	0.42	0.16	-0.37	0.01	-1.37	0.39	0.13	-0.54	-0.49	-0.38	0.14
