# Supplementary material for: Cross-species conserved miRNA as biomarker of radiation injury over a wide dose range using nonhuman primate model
Source: PLoS One. 2024 Nov 21;19(11):e0311379. doi: 10.1371/journal.pone.0311379 (PMC11581275; doi:10.1371/journal.pone.0311379)
Supplement: S1 Table — B. List of 176 miRNAs’ log2 fold change values that emerged significantly expressed in at least one of the 15 analysis models using all dependent and independent variables. C. cnvd-miRNA: A display of the homologue sequences conserved between humans and NHPs. (ZIP) [file pone.0311379.s004.zip › S1C_Table.pdf]

S1C Table. cnvd-miRNA: A display of the homologue sequences conserved between human and NHP

Mapped Human miRNAs	NHP alignment	Human alignment			
hsa-let-7a-3p	------------CUAUACAAUCUACUGUCUUUCC--------------------------	------------CUAUACAAUCUACUGUCUUUC---------------------------			
hsa-let-7a-5p	-------------UGAGGUAGUAGGUUGUAUAGUU-------------------------	-------------UGAGGUAGUAGGUUGUAUAGUU-------------------------			
hsa-let-7b-3p	------------CUAUACAACCUACUGCCUUCCC--------------------------	------------CUAUACAACCUACUGCCUUCCC--------------------------			
hsa-let-7c-5p	-------------UGAGGUAGUAGGUUGUAUGGUU-------------------------	-------------UGAGGUAGUAGGUUGUAUGGUU-------------------------			
hsa-let-7d-3p	------CCUAGGAAGAGGUAGUAGGUUGCAUAGUUUUAGGGCAGGGAUUUUGCCCACAAG	------------CUAUACGACCUGCUGCCUUUCU--------------------------			
hsa-let-7d-5p	------CCUAGGAAGAGGUAGUAGGUUGCAUAGUUUUAGGGCAGGGAUUUUGCCCACAAG	-------------AGAGGUAGUAGGUUGCAUAGUU-------------------------			
hsa-let-7e-5p	-------------UGAGGUAGGAGGUUGUAUAGUU-------------------------	-------------UGAGGUAGGAGGUUGUAUAGUU-------------------------			
hsa-let-7f-1-3p	------------CUAUACAAUCUAUUGCCUUCCC--------------------------	------------CUAUACAAUCUAUUGCCUUCCC--------------------------			
hsa-let-7f-5p	-------------UGAGGUAGUAGAUUGUAUAGUU-------------------------	-------------UGAGGUAGUAGAUUGUAUAGUU-------------------------			
hsa-let-7g-3p	------------CUGUACAGGCCACUGCCUUGCC--------------------------	------------CUGUACAGGCCACUGCCUUGC---------------------------			
hsa-let-7g-5p	-------------UGAGGUAGUAGUUUGUACAGUU-------------------------	-------------UGAGGUAGUAGUUUGUACAGUU-------------------------			
hsa-let-7i-5p	-------------UGAGGUAGUAGUUUGUGCUGUU-------------------------	-------------UGAGGUAGUAGUUUGUGCUGUU-------------------------			
hsa-miR-101-3p	------------UACAGUACUGUG-AUAACUGAAG-------------------------	------------UACAGUACUGUG-AUAACUGAA--------------------------			
hsa-miR-101-5p	--------------UCAGUUAUCACAGUGCUGAUGCU-----------------------	---------------CAGUUAUCACAGUGCUGAUGCU-----------------------			
hsa-miR-106b-3p	----------ACCGCACUGUGG-GUACUUGCUG---------------------------	-----------CCGCACUGUGG-GUACUUGCUGC--------------------------			
hsa-miR-106b-5p	-----------UAAAGUGCUGACAGUGCAGAU----------------------------	-----------UAAAGUGCUGACAGUGCAGAU----------------------------			
hsa-miR-10a-3p	-------------AAAUUCGUAUCUAGGGGAAUA--------------------------	------------CAAAUUCGUAUCUAGGGGAAUA--------------------------			
hsa-miR-10a-5p	----------------UACCCUGUAGAUCCG--AAUUUGUG-------------------	----------------UACCCUGUAGAUCCG--AAUUUGUG-------------------			
hsa-miR-1180-3p	---------UUUCCGGCUCGCGUGGGUGUGU-----------------------------	---------UUUCCGGCUCGCGUGGGUGUGU-----------------------------			
hsa-miR-1185-1-3p	-------------UAUACAGGGGGAGACUCUUA---------------------------	------------AUAUACAGGGGGAGACUCUUAU--------------------------			
hsa-miR-1247-5p	-------------ACCCGUCCCGUUCGUCCCCGGA-------------------------	-------------ACCCGUCCCGUUCGUCCCCGGA-------------------------			
hsa-miR-125b-1-3p	-----------ACGGGUUAGGCUCUUGGGAGCU---------------------------	-----------ACGGGUUAGGCUCUUGGGAGCU---------------------------			
hsa-miR-125b-2-3p	-----------ACAAGUCAGGCUCUUGGGACCU---------------------------	---------UCACAAGUCAGGCUCUUGGGAC-----------------------------			
hsa-miR-125b-5p	------------------UCCCUGAGACCCU--AACUUGUGA------------------	------------------UCCCUGAGACCCU--AACUUGUGA------------------			
hsa-miR-1260b	-------------AUCCCACCACUGCCACCA-----------------------------	-------------AUCCCACCACUGCCACCAU----------------------------			
hsa-miR-1271-3p	---------------GUGCCUGCUAUGUGCCGGGCA------------------------	--------------AGUGCCUGCUAUGUGCCAGGCA------------------------			
hsa-miR-128-3p	----------------UCACAGUGAACCGGUCUCUUUU----------------------	----------------UCACAGUGAACCGGUCUCUUU-----------------------			
hsa-miR-1296-5p	-------------UUAGGGCCC-UGGCUCCAUCUCCU-----------------------	-------------UUAGGGCCC-UGGCUCCAUCUCC------------------------			
hsa-miR-1306-5p	-----------CCACCUCCCCUGCAAACGUCC----------------------------	-----------CCACCUCCCCUGCAAACGUCCA---------------------------			
hsa-miR-130b-5p	-------ACUCUUUCCCUGUUGCA-CUACU------------------------------	-------ACUCUUUCCCUGUUGCA-CUAC-------------------------------			
hsa-miR-133a-3p	-----------UUGGUCCCCUUCAACCAGCUG----------------------------	----------UUUGGUCCCCUUCAACCAGCUG----------------------------			
hsa-miR-136-5p	-------ACUCCAUUUGUUUUGAUGAUGGA------------------------------	-------ACUCCAUUUGUUUUGAUGAUGGA------------------------------			
hsa-miR-140-3p	-------------UACCACAGGGUAGAACCACGG--------------------------	-------------UACCACAGGGUAGAACCACGG--------------------------			
hsa-miR-141-3p	------------------AACACUGUCUGGUAAAGAUGG---------------------	-----------------UAACACUGUCUGGUAAAGAUGG---------------------			
hsa-miR-142-3p	------------UGUAGUGUUUCCUACUUUAUGGA-------------------------	------------UGUAGUGUUUCCUACUUUAUGGA-------------------------			
hsa-miR-143-3p	---------------UGAGAUGAAGCACUGUAGCUC------------------------	---------------UGAGAUGAAGCACUGUAGCUC------------------------			
hsa-miR-146b-3p	---------AUGCCCUAUGGACUCAGUUC-UG----------------------------	-----------GCCCUGUGGACUCAGUUC-UGGU--------------------------			
hsa-miR-150-5p	-----------UCUCCCAACCCUUGUACCAGUG---------------------------	-----------UCUCCCAACCCUUGUACCAGUG---------------------------			
hsa-miR-152-3p	------------UCAGUGCAUGACAGAACUUGG---------------------------	------------UCAGUGCAUGACAGAACUUGG---------------------------			
hsa-miR-15b-5p	-----------UAGCAGCACAUCAUGGUUUACA---------------------------	-----------UAGCAGCACAUCAUGGUUUACA---------------------------			
hsa-miR-16-2-3p	-----------CCAAUAUUA-CUGUGCUGCUUCA--------------------------	-----------CCAAUAUUA-CUGUGCUGCUUUA--------------------------			
hsa-miR-181a-5p	---------------AACAUUCAACGCUGUCGGUGAGU----------------------	---------------AACAUUCAACGCUGUCGGUGAGU----------------------			
hsa-miR-181c-5p	---------------AACAUUCAACGCUGUCGGUGAGU----------------------	---------------AACAUUCAAC-CUGUCGGUGAGU----------------------			
hsa-miR-182-5p	---------------UUUGGCAAUGGUAGAACUCACA-----------------------	---------------UUUGGCAAUGGUAGAACUCACACU---------------------			
hsa-miR-183-5p	---------------UAUGGCACUGGUAGAAUUCACUG----------------------	---------------UAUGGCACUGGUAGAAUUCACU-----------------------			
hsa-miR-185-5p	------------UGGAGAGAAAG-GCAGUUCCUGA-------------------------	------------UGGAGAGAAAG-GCAGUUCCUGA-------------------------			
hsa-miR-186-5p	----------CAAAGAAUUCUC-CUUUUGGGCU---------------------------	----------CAAAGAAUUCUC-CUUUUGGGCU---------------------------			

hsa-miR-187-3p	------------UCGUGUCUUGU-GUUGCAGCCGG--------------------------	------------UCGUGUCUUGU-GUUGCAGCCGG--------------------------			
hsa-miR-18a-3p	-----------ACUGCCCUAAGUGCUCCUUCUGGC-------------------------	-----------ACUGCCCUAAGUGCUCCUUCUGG--------------------------			
hsa-miR-192-5p	---------------CUGACCUAUGAAUUGACAGCC------------------------	---------------CUGACCUAUGAAUUGACAGCC------------------------			
hsa-miR-193a-3p	-------------AACUGGCCUACAAAGUCCCAGU-------------------------	-------------AACUGGCCUACAAAGUCCCAGU-------------------------			
hsa-miR-193b-3p	-------------AACUGGCCCUCAAAGUCCCGCU-------------------------	-------------AACUGGCCCUCAAAGUCCCGCU-------------------------			
hsa-miR-195-5p	--------UAGCAGCACAGAAAUAUUGGC-------------------------------	--------UAGCAGCACAGAAAUAUUGGC-------------------------------			
hsa-miR-197-3p	---------------UUCACCACCUUCUCCACCCAGC-----------------------	---------------UUCACCACCUUCUCCACCCAGC-----------------------			
hsa-miR-199a-3p	-------------ACAGUAGUCUGCACAUUGGUUA-------------------------	-------------ACAGUAGUCUGCACAUUGGUUA-------------------------			
hsa-miR-199a-5p	-------------ACAGUAGUCUGCACAUUGGUUA-------------------------	---------------CCCAGUGUUCAGACUACCUGUUC----------------------			
hsa-miR-200a-3p	-----------------UAACACUGUCUGGUAACGAUGU---------------------	-----------------UAACACUGUCUGGUAACGAUGU---------------------			
hsa-miR-203a-3p	-------------GUGAAAUGUUUAGGACCACUAG-------------------------	-------------GUGAAAUGUUUAGGACCACUAG-------------------------			
hsa-miR-205-5p	---------UCCUUCAUUC-CACCGGAGUCUG-----------------------------	---------UCCUUCAUUC-CACCGGAGUCUG-----------------------------			
hsa-miR-210-3p	---------------CUGUGCGUGUGACAGCGGCUGA-----------------------	---------------CUGUGCGUGUGACAGCGGCUGA-----------------------			
hsa-miR-210-5p	--------------AGCCCCUGCCCACCGCACACUG------------------------	--------------AGCCCCUGCCCACCGCACACUG------------------------			
hsa-miR-21-3p	-----------CAACA-CCAGUCGAUGGGCUGUC--------------------------	-----------CAACA-CCAGUCGAUGGGCUGU---------------------------			
hsa-miR-214-3p	-------------ACAGCAGGCACAGACAGGCAG--------------------------	-------------ACAGCAGGCACAGACAGGCAGU-------------------------			
hsa-miR-215-5p	---------------AUGACCUAUGAAUUGACAGAC------------------------	---------------AUGACCUAUGAAUUGACAGAC------------------------			
hsa-miR-21-5p	------------UAGCUUAUC-AGACUGAUGUUGA-------------------------	------------UAGCUUAUC-AGACUGAUGUUGA-------------------------			
hsa-miR-22-3p	----------AAGCUGCCAGUUGAAGAACUGU----------------------------	----------AAGCUGCCAGUUGAAGAACUGU----------------------------			
hsa-miR-221-3p	------------AGCUACAU-UGUCUG--CUGGGUUUC----------------------	------------AGCUACAU-UGUCUG--CUGGGUUUC----------------------			
hsa-miR-223-3p	------------UGUCAGUUUGUC-AAAUACCCC--------------------------	------------UGUCAGUUUGUC-AAAUACCCCA-------------------------			
hsa-miR-23a-3p	--------------AUCACAUUGCCAGGGAUUUCC-------------------------	--------------AUCACAUUGCCAGGGAUUUCC-------------------------			
hsa-miR-25-3p	-----------------CAUUGCACUUGUCUCGGUCUGA---------------------	-----------------CAUUGCACUUGUCUCGGUCUGA---------------------			
hsa-miR-26a-5p	-------------UUCAAGUAAUC-CAGGAUAGGCU------------------------	-------------UUCAAGUAAUC-CAGGAUAGGCU------------------------			
hsa-miR-26b-5p	-------------UUCAAGUAAUU-CAGGAUAGGU-------------------------	-------------UUCAAGUAAUU-CAGGAUAGGU-------------------------			
hsa-miR-27b-3p	-------------UUCACAGUGGCUAA-GUUCUGC-------------------------	-------------UUCACAGUGGCUAA-GUUCUGC-------------------------			
hsa-miR-28-3p	-----CACUAGAUUGUGAGCUCCUGGA---------------------------------	-----CACUAGAUUGUGAGCUCCUGGA---------------------------------			
hsa-miR-296-5p	-----------AGGGCCCCCCUCAAUCCUGU-----------------------------	-----------AGGGCCCCCCUCAAUCCUGU-----------------------------			
hsa-miR-29a-3p	-------------CUAGCACCAUCUGAAAUCGGUU-------------------------	--------------UAGCACCAUCUGAAAUCGGUUA------------------------			
hsa-miR-29c-3p	--------------UAGCACCAUUUGAAAUCGGUUA------------------------	--------------UAGCACCAUUUGAAAUCGGUUA------------------------			
hsa-miR-301a-3p	--------------CAGUGCAAUAGUAUUGUCAAAGC-----------------------	--------------CAGUGCAAUAGUAUUGUCAAAGC-----------------------			
hsa-miR-301b-3p	--------------CAGUGCAAUGAUAUUGUCAAAGC-----------------------	--------------CAGUGCAAUGAUAUUGUCAAAGC-----------------------			
hsa-miR-30a-3p	--------CUUUCAGUCGGAUGUUUGCAGC------------------------------	--------CUUUCAGUCGGAUGUUUGCAGC------------------------------			
hsa-miR-30a-5p	-----------UGUAAACAUCCUCGACUGGAAG---------------------------	-----------UGUAAACAUCCUCGACUGGAAG---------------------------			
hsa-miR-30b-5p	-----------UGUAAACAUCCUACACUC--AGC--------------------------	-----------UGUAAACAUCCUACACUC--AGCU-------------------------			
hsa-miR-30c-5p	-----------UGUAAACAUCCUACACUCUCAGC--------------------------	-----------UGUAAACAUCCUACACUCUCAGC--------------------------			
hsa-miR-30d-3p	---------UUUCAGUCAGAUGUUUGCUGC------------------------------	--------CUUUCAGUCAGAUGUUUGCUGC------------------------------			
hsa-miR-3122	----------GUUGGGACAAGAGAACGGUCU-----------------------------	----------GUUGGGACAAGAGGACGGUCUU----------------------------			
hsa-miR-320b	------------AAAAGCUGGGUUGAGAGGGCAA--------------------------	------------AAAAGCUGGGUUGAGAGGGCAA--------------------------			
hsa-miR-324-3p	-----------ACUGCCCCAGGUGCUGCUGG-----------------------------	--------CCCACUGCCCCAGGUGCUGCUGG-----------------------------			
hsa-miR-328-3p	------------CUGGCCCUCUCUGCCCUUCC----------------------------	------------CUGGCCCUCUCUGCCCUUCCGU--------------------------			
hsa-miR-331-3p	-------------GCCCCUGGGCCUAUCCUAGAA--------------------------	-------------GCCCCUGGGCCUAUCCUAGAA--------------------------			
hsa-miR-331-5p	--------CUAGGUAUGG--UCCCAGGGAUCC----------------------------	--------CUAGGUAUGG--UCCCAGGGAUCC----------------------------			
hsa-miR-338-5p	------------AACAAUAUC-CUGGUGCUGAGUG-------------------------	------------AACAAUAUC-CUGGUGCUGAGUG-------------------------			
hsa-miR-342-3p	---------------UCUCACA-CAGAAAUCGCACCCGU---------------------	---------------UCUCACA-CAGAAAUCGCACCCGU---------------------			
hsa-miR-34a-5p	-----------UGGCAGUGUC-UUAGCUGGUUGU--------------------------	-----------UGGCAGUGUC-UUAGCUGGUUGU--------------------------			
hsa-miR-361-3p	--------------UCCCCCAGGUG-UGAUUCUGAUUU----------------------	--------------UCCCCCAGGUG-UGAUUCUGAUUU----------------------			
hsa-miR-362-3p	----------AACACACCUAUUCAAGGAUUCA----------------------------	----------AACACACCUAUUCAAGGAUUCA----------------------------			
hsa-miR-363-3p	------------------AAUUGCACGGUAUCCAUCUGUA--------------------	------------------AAUUGCACGGUAUCCAUCUGUA--------------------			

hsa-miR-369-3p	----------AAUAAUACAUGGUUGAUCUUU----------------------------	----------AAUAAUACAUGGUUGAUCUUU----------------------------			
hsa-miR-369-5p	------------AGAUCGACCGUGUUAUAUUCGC--------------------------	------------AGAUCGACCGUGUUAUAUUCGC--------------------------			
hsa-miR-374b-5p	-------AUAUAAUACAACCUGCUAAGUG------------------------------	-------AUAUAAUACAACCUGCUAAGUG------------------------------			
hsa-miR-375-3p	-----------UUUGUUCGUUCGGCUCGCGUGA--------------------------	-----------UUUGUUCGUUCGGCUCGCGUGA--------------------------			
hsa-miR-376a-3p	--------------AUCAUAGAGGAAAAUCCACGU------------------------	--------------AUCAUAGAGGAAAAUCCACGU------------------------			
hsa-miR-376b-3p	--------------AUCAUAGAGGAAAAUCCAUGUU-----------------------	--------------AUCAUAGAGGAAAAUCCAUGUU-----------------------			
hsa-miR-376c-3p	--------------AACAUAGAGGAAAUUCCACGU------------------------	--------------AACAUAGAGGAAAUUCCACGU------------------------			
hsa-miR-377-3p	-----------AUCACACAAAGGCAACUUUUGU--------------------------	-----------AUCACACAAAGGCAACUUUUGU--------------------------			
hsa-miR-378a-3p	--------------ACUGGACUUGGAGUCAGAAGG------------------------	--------------ACUGGACUUGGAGUCAGAAGGC-----------------------			
hsa-miR-378f	--------------ACUGGACUUGGAGUCAGAAGCA-----------------------	--------------ACUGGACUUGGAGCCAGAAG-------------------------			
hsa-miR-378g	--------------ACUGGACUUGGAGUCAGAAGCA-----------------------	--------------ACUGGGCUUGGAGUCAGAAG-------------------------			
hsa-miR-380-3p	----------UAUGUAAUAUGGUCCACGUCUU---------------------------	----------UAUGUAAUAUGGUCCACAUCUU---------------------------			
hsa-miR-381-3p	-------------UAUACAAGGGCAAGCUCUCUGU------------------------	-------------UAUACAAGGGCAAGCUCUCUGU------------------------			
hsa-miR-409-3p	---------GAAUGUUGCUCGGUGAACCCCU----------------------------	---------GAAUGUUGCUCGGUGAACCCCU----------------------------			
hsa-miR-411-5p	--------------UAGUAGACCG--UAUAGCGUACG----------------------	--------------UAGUAGACCG--UAUAGCGUACG----------------------			
hsa-miR-423-3p	-----------AGCUCGGUCUG-AGGCCCCUCAGU------------------------	-----------AGCUCGGUCUG-AGGCCCCUCAGU------------------------			
hsa-miR-423-5p	-----------UGAGGGGCAG-AGAGCGAGACUUU------------------------	-----------UGAGGGGCAG-AGAGCGAGACUUU------------------------			
hsa-miR-424-3p	---------------AAAACGUG--AGGCGCUGCUAUA---------------------	--------------CAAAACGUG--AGGCGCUGCUAU----------------------			
hsa-miR-424-5p	-----------CAGCAGCAAUUCAUGUUUUGAA--------------------------	-----------CAGCAGCAAUUCAUGUUUUGAA--------------------------			
hsa-miR-425-5p	----------AAUGACACGAUCACUCCCGUUGA--------------------------	----------AAUGACACGAUCACUCCCGUUGA--------------------------			
hsa-miR-429	-----------------UAAUACUGUCUGGUAAAACCGU--------------------	-----------------UAAUACUGUCUGGUAAAACCGU--------------------			
hsa-miR-433-3p	--------------AUCAUGAUGGGCUCCUCGGUGU-----------------------	--------------AUCAUGAUGGGCUCCUCGGUGU-----------------------			
hsa-miR-4446-3p	--------------CAGGGCUG--GCAGUGAGAUGGG----------------------	--------------CAGGGCUG--GCAGUGACAUGGGU---------------------			
hsa-miR-451a	-------------AAACCGUUACCAUUACUGAGUU------------------------	-------------AAACCGUUACCAUUACUGAGUU------------------------			
hsa-miR-484	----------UCAGGCUCAGUCCCCUCCCGAU---------------------------	----------UCAGGCUCAGUCCCCUCCCGAU---------------------------			
hsa-miR-485-3p	-----------GUCAUACACGGCUCUCCUCUCU--------------------------	-----------GUCAUACACGGCUCUCCUCUCU--------------------------			
hsa-miR-485-5p	----------AGAGGCUGGCCGUGAUGAAUUC---------------------------	----------AGAGGCUGGCCGUGAUGAAUUC---------------------------			
hsa-miR-486-5p	---------------UCCUGUACUGAGCUGCCCCGAG----------------------	---------------UCCUGUACUGAGCUGCCCCGAG----------------------			
hsa-miR-487b-3p	----------AAUCGUACAGGGUCAUCCACUU---------------------------	----------AAUCGUACAGGGUCAUCCACUU---------------------------			
hsa-miR-493-3p	------------UGAAGGUCUACUGUGUGCCAGG-------------------------	------------UGAAGGUCUACUGUGUGCCAGG-------------------------			
hsa-miR-493-5p	------------UUGUACAUGGUAGGCUUUCAUU-------------------------	------------UUGUACAUGGUAGGCUUUCAUU-------------------------			
hsa-miR-499a-5p	--------------UUAAGACU-UGCAGUGAUGUUU-----------------------	--------------UUAAGACU-UGCAGUGAUGUUU-----------------------			
hsa-miR-500a-3p	-----------AUGCACCUGGGCAAGGAUUCUGA-------------------------	-----------AUGCACCUGGGCAAGGAUUCUG--------------------------			
hsa-miR-505-3p	--------------CGUCAACACU-UGCUGGUUUCCU----------------------	--------------CGUCAACACU-UGCUGGUUUCCU----------------------			
hsa-miR-532-3p	------------CCUCCCACACCCAAGGCU-UGCA------------------------	------------CCUCCCACACCCAAGGCU-UGCA------------------------			
hsa-miR-543	----------AAACAUUCGCGGUGCACUUCUU---------------------------	----------AAACAUUCGCGGUGCACUUCUU---------------------------			
hsa-miR-548h-3p	---------AAAAACCG--CAAUUACUUUUGC---------------------------	--------CAAAAACCG--CAAUUACUUUUGCA--------------------------			
hsa-miR-550a-3p	---------------CUUACUCCCUCAGGCACAUC------------------------	------------UGUCUUACUCCCUCAGGCACAU-------------------------			
hsa-miR-550a-5p	--------------AGUGCCUGAGGG-GGUAAGAGCCC---------------------	--------------AGUGCCUGAGGG-AGUAAGAGCCC---------------------			
hsa-miR-574-3p	------------CACGCUCAUGCACACACCCACA-------------------------	------------CACGCUCAUGCACACACCCACA-------------------------			
hsa-miR-590-3p	-------------UAAUUUUAUGUAUAAGCUGGU-------------------------	-------------UAAUUUUAUGUAUAAGCUAGU-------------------------			
hsa-miR-598-3p	------------UACGUCAUCGU-UGUCAUCGUCA------------------------	------------UACGUCAUCGU-UGUCAUCGUCA------------------------			
hsa-miR-636	--------------UGUGCUUGCUCGUCCCGCCUGCA----------------------	--------------UGUGCUUGCUCGUCCCGCCCGCA----------------------			
hsa-miR-652-3p	--------------AAUGGCGCCACUAGGGU-UGUG-----------------------	--------------AAUGGCGCCACUAGGGU-UGUG-----------------------			
hsa-miR-654-3p	-----------UAUGUCUGCUGACCAUCACCUU--------------------------	-----------UAUGUCUGCUGACCAUCACCUU--------------------------			
hsa-miR-654-5p	--------------UGGUGGGCCG--CAGAACAUGUGC---------------------	--------------UGGUGGGCCG--CAGAACAUGUGC---------------------			
hsa-miR-665	----------ACCAGGAGGCU-GAGGCCCCU----------------------------	----------ACCAGGAGGCU-GAGGCCCCU----------------------------			
hsa-miR-671-3p	-------------UCCGGUUCUCAGGGCUCCACC-------------------------	-------------UCCGGUUCUCAGGGCUCCACC-------------------------			
hsa-miR-760	--------------CGGCUC-UGGGUCUGUGGGGA------------------------	--------------CGGCUC-UGGGUCUGUGGGGA------------------------			

hsa-miR-874-3p	------------CUGCCCUGGCCCGAGGGACCGA--------------------------	------------CUGCCCUGGCCCGAGGGACCGA--------------------------			
hsa-miR-889-3p	------------UUAAUAUCGGACAACCAUUGU---------------------------	------------UUAAUAUCGGACAACCAUUGU---------------------------			
hsa-miR-92a-3p	-----------------UAUUGCACUUGUCCCGGCCUGU---------------------	-----------------UAUUGCACUUGUCCCGGCCUGU---------------------			
hsa-miR-92b-3p	-----------------UAUUGCACUCGUCCCGGCCUCC---------------------	-----------------UAUUGCACUCGUCCCGGCCUCC---------------------			
hsa-miR-93-3p	---------ACUGCUGAGCUAGCACUUCCCG-----------------------------	---------ACUGCUGAGCUAGCACUUCCCG-----------------------------			
hsa-miR-942-5p	------------CUUCUCUGU-UUUGGCCAUGUG--------------------------	-----------UCUUCUCUGU-UUUGGCCAUGUG--------------------------			
