# Supplementary material for: Cross-species conserved miRNA as biomarker of radiation injury over a wide dose range using nonhuman primate model
Source: PLoS One. 2024 Nov 21;19(11):e0311379. doi: 10.1371/journal.pone.0311379 (PMC11581275; doi:10.1371/journal.pone.0311379)
Supplement: S2 Table — (PDF) [file pone.0311379.s005.pdf]

S2 Table. List of miRNAs linked to major bionetworks

TBI*RD		
Apoptosis	Invasion of cells	Cell proliferation of tumor cell lines
let-7a-5p	let-7a-5p	let-7a-5p
miR-130a-3p	miR-141-3p	miR-130a-3p
miR-141-3p	miR-16-5p	miR-141-3p
miR-16-5p	miR-223-3p	miR-16-5p
miR-223-3p	miR-23a-3p	miR-223-3p
miR-23a-3p	miR-30c-5p	miR-23a-3p
miR-30c-5p	miR-451a	miR-30a-3p
miR-451a	miR-654-3p	miR-30c-5p
miR-654-3p	miR-92a-3p	miR-451a
		miR-485-5p
		miR-654-3p
		miR-92a-3p

RRiF			
Invasion of cells	Invasion of tumor cell lines	Quantity of cells	Quantity of muscle cell lines
let-7a-5p	miR-125b-5p	miR-125b-5p	miR-125b-5p
miR-125b-5p	miR-16-5p	miR-16-5p	miR-361-3p
miR-16-5p	miR-23a-3p	miR-361-3p	miR-486-5p
miR-23a-3p	miR-451a	miR-451a	miR-574-3p
miR-451a	miR-92a-3p	miR-486-5p	
miR-92a-3p		miR-574-3p	
		miR-92a-3p	

TBI*RRiF
Cell proliferation of tumor cell lines
let-7a-5p
miR-181a-5p
miR-199a-5p
miR-23a-3p
miR-380-3p
miR-409-3p
miR-532-3p
miR-541-3p
miR-654-3p
miR-889-3p
miR-92a-3p
