# Supplementary material for: Cross-species conserved miRNA as biomarker of radiation injury over a wide dose range using nonhuman primate model
Source: PLoS One. 2024 Nov 21;19(11):e0311379. doi: 10.1371/journal.pone.0311379 (PMC11581275; doi:10.1371/journal.pone.0311379)
Supplement: S3 Table — B. Correlation matrix of 11 networks that were significantly regulated by RD*TSI. Pearson correlation was calculated across radiation doses at 6-day post-TBI. C. Male vs. female correlation matrix of 7 networks that were significantly regulated by sex*RD*TSI. Pearson correlation was calculated across radiation doses and TSI. (ZIP) [file pone.0311379.s006.zip › S3A_Table.pdf]

S3A Table. Correlation matrix of 11 networks that were significantly regulated by RD*TSR. Pearson correlation was calculated across entire dosimetry and TSR.

	Invasion of tumor cell lines	Invasion of cells	Apoptosis of tumor cell lines	Migration of tumor cell lines	Cell proliferation of tumor cell lines	Apoptosis	Migration of cells	Cell viability of tumor cell lines	Cell proliferation of colorectal cancer cell lines	Metastasis	Cell proliferation of carcinoma cell lines
Invasion of tumor cell lines	1	0.95	-0.75	-0.13	0.72	-0.38	-0.2	0.72	0.58	0.41	0.62
Invasion of cells	0.95	1	-0.56	-0.29	0.56	-0.21	-0.35	0.6	0.57	0.24	0.55
Apoptosis of tumor cell lines	-0.75	-0.56	1	-0.16	-0.81	0.84	-0.18	-0.87	-0.37	-0.48	-0.54
Migration of tumor cell lines	-0.13	-0.29	-0.16	1	0.24	-0.03	0.97	0.15	0.21	-0.17	0.25
Cell proliferation of tumor cell lines	0.72	0.56	-0.81	0.24	1	-0.5	0.24	0.71	0.57	0.3	0.72
Apoptosis	-0.38	-0.21	0.84	-0.03	-0.5	1	-0.11	-0.61	0.03	-0.52	-0.18
Migration of cells	-0.2	-0.35	-0.18	0.97	0.24	-0.11	1	0.19	0.19	-0.13	0.29
Cell viability of tumor cell lines	0.72	0.6	-0.87	0.15	0.71	-0.61	0.19	1	0.52	0.2	0.65
Cell proliferation of colorectal cancer cell lines	0.58	0.57	-0.37	0.21	0.57	0.03	0.19	0.52	1	0.2	0.64
Metastasis	0.41	0.24	-0.48	-0.17	0.3	-0.52	-0.13	0.2	0.2	1	0.2
Cell proliferation of carcinoma cell lines	0.62	0.55	-0.54	0.25	0.72	-0.18	0.29	0.65	0.64	0.2	1
