# Supplementary material for: Cross-species conserved miRNA as biomarker of radiation injury over a wide dose range using nonhuman primate model
Source: PLoS One. 2024 Nov 21;19(11):e0311379. doi: 10.1371/journal.pone.0311379 (PMC11581275; doi:10.1371/journal.pone.0311379)
Supplement: S3 Table — B. Correlation matrix of 11 networks that were significantly regulated by RD*TSI. Pearson correlation was calculated across radiation doses at 6-day post-TBI. C. Male vs. female correlation matrix of 7 networks that were significantly regulated by sex*RD*TSI. Pearson correlation was calculated across radiation doses and TSI. (ZIP) [file pone.0311379.s006.zip › S3C_Table.pdf]

S3C Table. Male vs. female correlation matrix of 7 networks that were significantly regulated by sex*RD*TSR. Pearson correlation was calculated across entire dosimetry and TSR.

	6Gy	6,5Gy	7Gy	7.5Gy	8Gy	8.6Gy
Apoptosis	-0.05	-0.02	0.18	0.39	0.77	0.35
Apoptosis of tumor cell lines	-0.72	-0.94	-0.74	0.43	N/A	0.83
Cell proliferation of carcinoma cell lines	0.61	-0.28	N/A	N/A	0.62	0.96
Cell proliferation of tumor cell lines	-0.62	-0.53	-0.79	-0.86	0.27	0.89
Invasion of cells	-1	0.48	-1	-0.1	-0.89	0.76
Invasion of tumor cell lines	-0.4	0	0.6	N/A	N/A	0.97
Migration of tumor cell lines	0.5	-0.91	1	N/A	N/A	-0.96
