# Supplementary material for: Cross-species conserved miRNA as biomarker of radiation injury over a wide dose range using nonhuman primate model
Source: PLoS One. 2024 Nov 21;19(11):e0311379. doi: 10.1371/journal.pone.0311379 (PMC11581275; doi:10.1371/journal.pone.0311379)
Supplement: S4 Table — B. RSBMR results: List of potential panels that can predict RRiF with AUC >0.82. (ZIP) [file pone.0311379.s007.zip › S4A_Table.pdf]

S4A Table. K-fold results: List of potential panels that can predict RRiF with AUC >0.85

Features	NumberOf	AUC	Specificity	Sensitivity	P.Value	McFadden	Adjusted.R	Intercept	min (95% (	mean/med	max(95% (	Feature1	Feature2	Feature3	Feature4	Feature5	Feature6
mml-miR-376c-3p, mml-miR-342-3p, mml-miR-363-3p	3	0.8651960	0.875	0.5882352	0	0.1429934	0.1159300	0.5796344	0.535119	0.698529	0.86194	-0.405648	-0.384341	0.677038260350885			
mml-miR-376c-3p, mml-miR-221-3p, mml-miR-342-3p, mml-miR-363-3p	4	0.8602941	0.875	0.5882352	0	0.1432300	0.1067717	0.5974652	0.503866	0.671569	0.839271	-0.413010	0.0478296	-0.379207	0.693305853159143		
mml-miR-377-3p, mml-miR-92b-3p, mml-miR-143-3p, mml-miR-26b-5p, mml-miR-342-3p, mml-miR-363-3p	6	0.8578431	0.8333333	0.6470588	0	0.1834890	0.1302383	0.6181608	0.474099	0.64951	0.82492	-0.332985	0.2125211	0.3515987	-0.272515	-0.351172	0.47794852416934
mml-miR-377-3p, mml-miR-92b-3p, mml-miR-143-3p, mml-miR-342-3p, mml-miR-363-3p	5	0.8553921	0.7916666	0.7058823	0	0.1800806	0.1359989	0.6746918	0.467933	0.644608	0.821282	-0.319080	0.2886598	0.3374702	-0.388172	0.472686388485167	
mml-miR-376c-3p, mml-miR-143-3p, mml-miR-342-3p, mml-miR-363-3p	4	0.8504901	0.875	0.6470588	0	0.1636954	0.1281080	0.5740784	0.54936	0.708333	0.867307	-0.371711	0.3276835	-0.385963	0.701373933591616		
mml-miR-376c-3p, mml-miR-143-3p, mml-miR-301a-3p, mml-miR-342-3p, mml-miR-363-3p	5	0.8504901	0.8333333	0.6470588	0	0.1927581	0.1493580	0.4264428	0.582207	0.735294	0.888382	-0.385160	0.3123976	-0.682845	-0.293152	0.657033243027212	
mml-miR-376c-3p, mml-miR-143-3p, mml-miR-221-3p, mml-miR-342-3p, mml-miR-363-3p	5	0.8504901	0.875	0.6470588	0	0.1636980	0.1187356	0.5721281	0.512324	0.678922	0.845519	-0.370866	0.3280911	-0.005115	-0.386538	0.699631175088491	
