# Supplementary material for: Cross-species conserved miRNA as biomarker of radiation injury over a wide dose range using nonhuman primate model
Source: PLoS One. 2024 Nov 21;19(11):e0311379. doi: 10.1371/journal.pone.0311379 (PMC11581275; doi:10.1371/journal.pone.0311379)
Supplement: S4 Table — B. RSBMR results: List of potential panels that can predict RRiF with AUC >0.82. (ZIP) [file pone.0311379.s007.zip › S4B_Table.pdf]

S4B Table. RSBMR results: List of potential panels that can predict RRiF with AUC >0.82

Index	Features	NumberO	Average.A	StandardD	Average.S	StandardD	Average.S	StandardD	Average.P	StandardD	Average.N	StandardD	Average.A	StandardD	min (95%	mean/me	max(95%
18737	mml-miR-92b-3p, mml-miR-106b-3p, mml-miR-143-3p, mml-miR-23a-3p, mml-miR-28-3p, mml-miR-342-3p, mml-miR-363-3p	7	0.829656	0.048681	0.8	0.087400	0.664705	0.117810	0	0	0.282719	0.040285	0.227543	0.043384	0.64769	0.79167	0.93564
7230	mml-miR-92a-3p, mml-miR-106b-3p, mml-miR-143-3p, mml-miR-23a-3p, mml-miR-28-3p, mml-miR-342-3p, mml-miR-363-3p	7	0.827450	0.052836	0.8125	0.083910	0.641176	0.128427	0	0	0.283362	0.041562	0.228236	0.044759	0.67189	0.81127	0.95066
13305	mml-miR-377-3p, mml-miR-484, mml-miR-143-3p, mml-miR-342-3p, mml-miR-363-3p, mml-miR-369-5p	6	0.825	0.036339	0.825	0.061488	0.688235	0.083419	0	0	0.246701	0.030459	0.197573	0.032445	0.63444	0.77696	0.91948
7215	mml-miR-484, mml-miR-106b-3p, mml-miR-143-3p, mml-miR-23a-3p, mml-miR-28-3p, mml-miR-342-3p, mml-miR-363-3p	7	0.824509	0.046420	0.825	0.085165	0.652941	0.070424	0	0	0.290689	0.040554	0.236127	0.043673	0.68159	0.81618	0.95076
7251	mml-miR-484, mml-miR-92a-3p, mml-miR-106b-3p, mml-miR-143-3p, mml-miR-23a-3p, mml-miR-28-3p, mml-miR-342-3p, mml-miR-363-3p	8	0.823529	0.049060	0.816666	0.086066	0.652941	0.080607	0	0	0.294766	0.040979	0.232078	0.044621	0.67297	0.81127	0.94958
18751	mml-miR-92a-3p, mml-miR-92b-3p, mml-miR-106b-3p, mml-miR-143-3p, mml-miR-23a-3p, mml-miR-28-3p, mml-miR-342-3p, mml-miR-363-3p	8	0.823284	0.049405	0.8	0.091708	0.658823	0.116991	0	0	0.286970	0.040363	0.223590	0.043951	0.63665	0.78431	0.93198
2377	mml-miR-92b-3p, mml-miR-143-3p, mml-miR-23a-3p, mml-miR-28-3p, mml-miR-342-3p, mml-miR-363-3p	6	0.823039	0.049112	0.804166	0.094301	0.652941	0.112467	0	0	0.270003	0.044201	0.222394	0.047084	0.60757	0.7598	0.91203
18752	mml-miR-92b-3p, mml-miR-106b-3p, mml-miR-143-3p, mml-miR-23a-3p, mml-miR-28-3p, mml-miR-301a-3p, mml-miR-342-3p, mml-miR-363-3p	8	0.822303	0.047501	0.8	0.103563	0.670588	0.100746	0	0	0.291106	0.039720	0.228093	0.043251	0.64146	0.78922	0.93697
13159	mml-miR-377-3p, mml-miR-484, mml-miR-143-3p, mml-miR-342-3p, mml-miR-363-3p	5	0.822058	0.034044	0.854166	0.068746	0.664705	0.078676	0	0	0.233461	0.029728	0.192250	0.031326	0.63679	0.77941	0.92203
18747	mml-miR-484, mml-miR-92b-3p, mml-miR-106b-3p, mml-miR-143-3p, mml-miR-23a-3p, mml-miR-28-3p, mml-miR-342-3p, mml-miR-363-3p	8	0.821813	0.045999	0.808333	0.096625	0.658823	0.077444	0	0	0.295258	0.039028	0.232614	0.042497	0.60188	0.76225	0.92263
36275	mml-miR-493-5p, mml-miR-92a-3p, mml-miR-106b-3p, mml-miR-143-3p, mml-miR-23a-3p, mml-miR-28-3p, mml-miR-342-3p, mml-miR-363-3p	8	0.821813	0.050337	0.8	0.105409	0.635294	0.140849	0	0	0.286046	0.041450	0.222583	0.045134	0.68088	0.81863	0.95638
2292	mml-miR-484, mml-miR-143-3p, mml-miR-23a-3p, mml-miR-28-3p, mml-miR-342-3p, mml-miR-363-3p	6	0.821568	0.051318	0.795833	0.079664	0.652941	0.115835	0	0	0.278749	0.044523	0.231711	0.047426	0.67003	0.80637	0.94272
7262	mml-miR-92a-3p, mml-miR-106b-3p, mml-miR-143-3p, mml-miR-23a-3p, mml-miR-28-3p, mml-miR-301a-3p, mml-miR-342-3p, mml-miR-363-3p	8	0.821568	0.048192	0.820833	0.090117	0.658823	0.110223	0	0	0.291826	0.041118	0.228877	0.044773	0.65417	0.79657	0.93896
15408	mml-miR-377-3p, mml-miR-484, mml-miR-143-3p, mml-miR-23a-3p, mml-miR-342-3p, mml-miR-363-3p	6	0.821568	0.049084	0.833333	0.073493	0.647058	0.107396	0	0	0.249473	0.037547	0.200526	0.039995	0.60346	0.75245	0.90144

Index	Average.I	StandardD	Average.F	StandardD	Average.F	StandardD	Average.F	StandardD	Average.F	StandardD	Average.F	StandardD	Average.F	StandardD	Average.F	StandardD	Average.F	StandardDev.Feature8
18737	0.189992	0.087881	0.414850	0.080199	-0.474066	0.225200	0.500865	0.106640	-0.843427	0.192369	-1.976539	0.300328	-0.469844	0.083564	0.696718	0.146252	NaN	
7230	0.208982	0.086415	0.606676	0.236835	-0.642992	0.296854	0.504128	0.098976	-0.791985	0.226985	-1.802627	0.293265	-0.435222	0.083934	0.696185	0.153749	NaN	
13305	0.727315	0.089704	-0.340857	0.098003	0.614211	0.149282	0.310442	0.086714	-0.435482	0.067007	0.562986	0.098944	-0.313333	0.115232	NaN		NaN	
7215	0.315898	0.085726	0.535881	0.169133	-0.386734	0.196115	0.483141	0.102594	-0.908425	0.169266	-1.806858	0.268665	-0.446484	0.084098	0.751125	0.176238	NaN	
7251	0.295003	0.078265	0.406401	0.213587	0.341409	0.272526	-0.607197	0.311130	0.483892	0.101273	-0.866453	0.218595	-1.797994	0.283384	-0.442012	0.085395	0.712274	0.167024954311643
18751	0.195758	0.091891	0.315370	0.452771	0.253534	0.235362	-0.608048	0.300990	0.501946	0.103779	-0.830172	0.223710	-1.910632	0.336880	-0.457293	0.076996	0.684350	0.150018495129543
2377	0.243877	0.099818	0.172040	0.074855	0.514409	0.107455	-0.686234	0.202853	-1.665816	0.256841	-0.452203	0.084659	0.618414	0.116535	NaN		NaN	
18752	0.137012	0.110347	0.372085	0.100312	-0.481100	0.228294	0.450704	0.126641	-0.874082	0.199176	-1.841296	0.377342	-0.323122	0.260968	-0.441234	0.088520	0.704342	0.148860731898511
13159	0.841350	0.070273	-0.416640	0.089932	0.625338	0.160781	0.347461	0.088679	-0.386971	0.066952	0.491094	0.128357	NaN		NaN		NaN	
18747	0.282684	0.091542	0.412216	0.256162	0.242401	0.161983	-0.526591	0.240730	0.482359	0.106145	-0.906284	0.194966	-1.904691	0.308042	-0.463989	0.084757	0.702441	0.16043021987 7357
36275	0.208534	0.100607	-0.098560	0.142337	0.601529	0.242522	-0.649465	0.305319	0.492435	0.104314	-0.768640	0.248172	-1.775878	0.291615	-0.440243	0.086730	0.706872	0.166987698950456
2292	0.321529	0.084621	0.351478	0.185591	0.492008	0.110699	-0.730912	0.204800	-1.565791	0.264490	-0.442785	0.083384	0.611522	0.146833	NaN		NaN	
7262	0.153602	0.110114	0.543282	0.239504	-0.628474	0.298510	0.450830	0.117378	-0.829525	0.232181	-1.683145	0.345499	-0.329475	0.250620	-0.409229	0.090276	0.701941	0.156638362824002
15408	0.814677	0.079273	-0.340590	0.096864	0.604861	0.179008	0.355728	0.090668	-0.504253	0.239986	-0.403426	0.070665	0.470120	0.140358	NaN		NaN	
