# Supplementary material for: Cross-species conserved miRNA as biomarker of radiation injury over a wide dose range using nonhuman primate model
Source: PLoS One. 2024 Nov 21;19(11):e0311379. doi: 10.1371/journal.pone.0311379 (PMC11581275; doi:10.1371/journal.pone.0311379)
Supplement: S5 Table — (PDF) [file pone.0311379.s008.pdf]

S5 Table. The miRNA list linked to the Venn diagram Figure S3

		A-B-C	A-B	A-C	B-C	A	B	C
		miR-133a-3p	miR-429	miR-215-5p	miR-126-5p	let-7f-5p	miR-708-3p	miR-133b-3p
A	Human-NHP conserved		miR-142-3p	miR-150-5p		miR-92a-3p	miR-133a-5p	
B	Human-minipig conserved		miR-424-5p	miR-30a-5p		miR-665	miR-221-5p	
C	Human-mouse-NHP conserved		miR-143-3p	miR-375-3p		miR-363-3p	miR-671-5p	
			miR-374b-5p			miR-548h-3p	miR-361-5p	
			miR-22-3p			miR-128-3p	miR-148a-5p	
			miR-1306-5p			let-7i-5p	miR-92b-5p	
			miR-296-5p			miR-152-3p	miR-139-5p	
			miR-30c-5p			miR-654-3p	miR-30c-1-3p	
			miR-28-3p			miR-369-5p	miR-374a-5p	
			miR-424-3p			miR-760	miR-1306-3p	
			miR-532-3p			let-7a-5p	miR-145-5p	
			miR-1296-5p			miR-197-3p	miR-30e-3p	
			miR-361-3p			miR-381-3p	miR-17-3p	
			miR-671-3p			miR-23a-3p	miR-145-3p	
			miR-193a-3p			miR-106b-5p	miR-676-3p	
			miR-331-5p			miR-185-5p	miR-202-3p	
			miR-27b-3p			miR-376c-3p	miR-455-3p	
			miR-221-3p			miR-369-3p	miR-425-3p	
			let-7d-5p			miR-499a-5p	miR-17-5p	
			miR-30a-3p			miR-378a-3p	miR-24-3p	
			miR-331-3p			miR-15b-5p	miR-432-5p	
			miR-376a-3p			miR-485-5p	miR-6782-3p	
			miR-199a-5p			miR-550a-5p	miR-148b-3p	
			miR-425-5p			miR-320b	miR-217	
			miR-30b-5p			miR-210-3p	miR-27b-5p	
						miR-183-5p	miR-339-3p	
						miR-141-3p	miR-345-5p	
						miR-301b-3p	miR-345-3p	
						miR-210-5p	miR-769-5p	
						miR-362-3p	miR-206	
						miR-199a-3p	miR-542-3p	
						miR-29c-3p	miR-708-5p	

						miR-125b-1-3p	miR-148a-3p	
						miR-136-5p	miR-455-5p	
						miR-26a-5p	miR-28-5p	
						miR-18a-3p	miR-296-3p	
						miR-195-5p	miR-885-5p	
						miR-130b-5p	miR-155-5p	
						let-7d-3p	miR-24-1-5p	
						miR-125b-5p	miR-193a-5p	
						miR-1185-1-3p	miR-148b-5p	
						miR-550a-3p	miR-769-3p	
						miR-34a-5p	miR-142-5p	
						miR-598-3p	miR-532-5p	
						miR-505-3p	miR-218-5p	
						let-7e-5p	miR-190b	
						miR-574-3p	miR-184	
						let-7g-5p	miR-542-5p	
						miR-21-5p	miR-490-3p	
						miR-4446-3p	miR-181d-5p	
						miR-342-3p	miR-450b-5p	
						miR-203a-3p	miR-885-3p	
						miR-106b-3p	miR-545-5p	
						miR-301a-3p	miR-199b-5p	
						miR-125b-2-3p	miR-326	
						miR-652-3p	miR-545-3p	
						miR-380-3p	miR-96-5p	
						miR-25-3p	miR-374a-3p	
						miR-92b-3p	miR-374b-3p	
						miR-493-5p	miR-22-5p	
						miR-1260b	miR-139-3p	
						miR-411-5p	miR-140-5p	
						miR-376b-3p	miR-133b	
						miR-193b-3p		
						miR-192-5p		
						miR-10a-5p		
						miR-378g		

						let-7b-3p		
						let-7a-3p		
						miR-377-3p		
						let-7g-3p		
						miR-423-5p		
						miR-493-3p		
						miR-3122		
						miR-487b-3p		
						miR-590-3p		
						miR-16-2-3p		
						miR-654-5p		
						let-7c-5p		
						miR-101-5p		
						miR-214-3p		
						miR-486-5p		
						miR-500a-3p		
						miR-223-3p		
						miR-451a		
						miR-187-3p		
						miR-205-5p		
						miR-181c-5p		
						miR-1180-3p		
						miR-181a-5p		
						miR-93-3p		
						miR-636		
						miR-423-3p		
						miR-484		
						miR-942-5p		
						miR-140-3p		
						miR-101-3p		
						miR-21-3p		
						miR-409-3p		
						miR-485-3p		
						miR-186-5p		
						let-7f-1-3p		

						miR-543		
						miR-30d-3p		
						miR-338-5p		
						miR-433-3p		
						miR-324-3p		
						miR-328-3p		
						miR-146b-3p		
						miR-1247-5p		
						miR-378f		
						miR-1271-3p		
						miR-200a-3p		
						miR-29a-3p		
						miR-874-3p		
						miR-26b-5p		
						miR-182-5p		
						miR-889-3p		
						miR-10a-3p		
